# A Population Genetic Model for the Maintenance of R2 Retrotransposons in rRNA Gene Loci

**DOI:** 10.1371/journal.pgen.1003179

**Published:** 2013-01-10

**Authors:** Jun Zhou, Michael T. Eickbush, Thomas H. Eickbush

**Affiliations:** Department of Biology, University of Rochester, Rochester, New York, United States of America; University of California Davis, United States of America

## Abstract

R2 retrotransposable elements exclusively insert into the tandemly repeated rRNA genes, the rDNA loci, of their animal hosts. R2 elements form stable long-term associations with their host, in which all individuals in a population contain many potentially active copies, but only a fraction of these individuals show active R2 retrotransposition. Previous studies have found that R2 RNA transcripts are processed from a 28S co-transcript and that the likelihood of R2-inserted units being transcribed is dependent upon their distribution within the rDNA locus. Here we analyze the rDNA locus and R2 elements from nearly 100 R2-active and R2-inactive individuals from natural populations of *Drosophila simulans*. Along with previous findings concerning the structure and expression of the rDNA loci, these data were incorporated into computer simulations to model the crossover events that give rise to the concerted evolution of the rRNA genes. The simulations that best reproduce the population data assume that only about 40 rDNA units out of the over 200 total units are actively transcribed and that these transcribed units are clustered in a single region of the locus. In the model, the host establishes this transcription domain at each generation in the region with the fewest R2 insertions. Only if the host cannot avoid R2 insertions within this 40-unit domain are R2 elements active in that generation. The simulations also require that most crossover events in the locus occur in the transcription domain in order to explain the empirical observation that R2 elements are seldom duplicated by crossover events. Thus the key to the long-term stability of R2 elements is the stochastic nature of the crossover events within the rDNA locus, and the inevitable expansions and contractions that introduce and remove R2-inserted units from the transcriptionally active domain.

## Author Summary

Selfish transposable elements survive in eukaryotic genomes despite the elaborate mechanisms developed by the hosts to limit their activity. One accessible system that simplifies the complex interactions between element and host involves the R2 elements, which exclusively insert in the tandemly arranged rRNA genes. R2 exhibits remarkable stability in animal lineages even though each insertion inactivates one rRNA gene. Here we determine the size of the rDNA locus and R2 number in natural isolates of *Drosophila simulans*. Combined with previous data concerning the expression and regulation of R2, we develop a detailed population genetic model for rRNA gene and R2 evolution that duplicates all properties of the rRNA loci in natural populations. Critical components of the model are that only a contiguous 40 unit array of rRNA gene units are needed for transcription, that R2 elements are active only when present in this transcription domain, and that most of the crossovers in the rDNA loci occur in this domain. These results suggest that the key to the long-term survival of R2 is the redistribution of rDNA units in the locus brought about by the crossovers that maintain sequence identity in all rDNA units.

## Introduction

**Figure 1:**
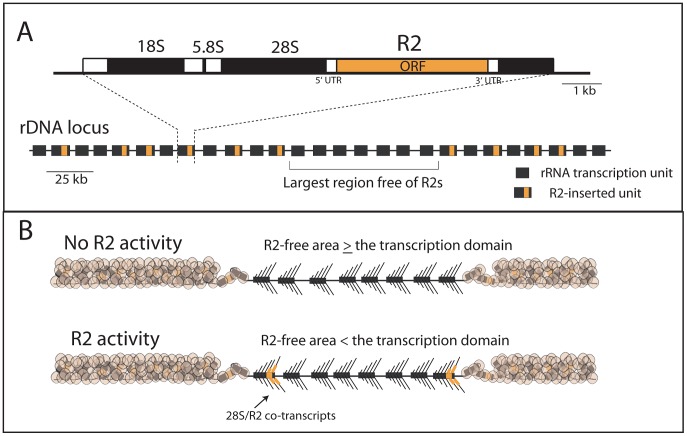
Diagram of the rDNA locus and how the distribution of R2 gives rise to R2-active and R2-inactive individuals. (A) rDNA loci are composed of tandem repeated rRNA genes with some 28S rRNA genes containing an R2 insertion. Each repeat contains one transcription unit with 18S, 5.8S and 28S rRNA genes (black bars) separated by spacer regions (open bars). R2 elements encode a large open reading frame, ORF, (orange bar) with short 5′ and 3′ untranslated regions (UTRs). The largest block of uninserted rDNA units is identified and determines what contiguous block of rDNA units are transcribed, the transcription domain. (B) The transcription domain model for the regulation of R2 activity is based on data suggesting that the host activates for transcription a contiguous block of rDNA units containing the fewest R2-inserted units [18], [20]. The transcription domain is centered on the largest contiguous area of uninserted rDNA units. The remaining rDNA units are packaged into a transcriptionally inactive chromatin form. If the largest area free of R2 insertions is larger than the transcription domain, then no transcription of R2-inserted units occur. If the largest area free of R2 insertions is smaller than the transcription domain, then transcription of R2-inserted units does occur giving rise to retrotransposition events.

Abundant ribosomal RNA (rRNA) is essential for cellular metabolism during all periods of development. The genes encoding these RNAs reside as nearly identical tandemly repeated units with each unit composed of an 18S, 5.8S and 28S rRNA gene (Figure 1A). Surprisingly, these tandem genes, referred to as rDNA loci, serve as a genomic niche for the insertion of various mobile elements [1]. These elements block the production of functional rRNA from inserted units, however, the effects of this potential disruption of rRNA production is minimized because organisms typically contain many more rDNA units than are needed for transcription [2]–[4].

The retrotransposon, R2, is the best understood of the rDNA specific elements. R2 elements are present in many animal phyla [5]–[7] but have been most intensively studied in *Drosophila* [8], [9]. The same lineage of R2 elements is present in most Drosophila groups, and no evidence has been found for horizontal jumps between species [8]. While difficult to establish definitively, this co-evolution of R2 with its host may extend back to the origin of the major animal phyla [6], [7], [10], [11]. Clearly a balance must be maintained between the levels of retrotransposition required to preserve the elements and the number of rDNA units needed to maintain host fitness.

While permitting long term maintenance, the equilibrium between the rDNA loci and R2 elements appears highly dynamic, as the size of the rDNA loci vary greatly between individuals, and individual copies of R2 are rapidly gained and lost from each locus [12], [13]. A critical contributor to this dynamic equilibrium is the frequent unequal crossovers between the tandem repeats of the rDNA loci, which preserve the high levels of sequence identity between rRNA genes (Figure S1). Attempts have been made over the years to model this concerted evolution of the rRNA genes [14]–[16]. Recently we incorporated the presence of transposable elements into standard crossover models of rDNA locus evolution [17]. Varying the rates of crossover, R2 retrotransposition, and the number of rDNA units required for host fitness, stable populations could be simulated with rDNA loci of various sizes and levels of R2 insertion. Unfortunately, because little was known about of the forces that controlled R2 activity, these simulations simply assumed low rates of retrotransposition in all individuals with R2.

Recent studies have now provided a better understanding of the regulation of R2 activity in *Drosophila simulans*. First, regulation of R2 activity appears to be at the level of transcription with control over transcription mapping to the rDNA locus itself [18]. Second, R2 elements do not encode their own promoter but are co-transcribed with the rDNA unit with their mature R2 transcript processed from the co-transcript by a ribozyme encoded at the 5′ end of R2 [19]. Finally, R2 transcription correlates best with the distribution of R2 elements across the rDNA locus rather than the size of the rDNA locus or the number of R2 insertions [20]. Animals with no R2 transcripts contain at least one large region of rDNA units free of R2, while animals with R2 transcripts contain a more uniform distribution of R2 across the rDNA locus and thus no large region free of R2 insertions [18], [20]. Based on these findings, we proposed a “transcription domain” model of R2 regulation in which the host identifies for transcription that region of the rDNA locus with the lowest level of R2 insertions. In this model, individual copies of R2 are transcribed only when the largest contiguous region of the rDNA locus free of R2 insertions is less than the size of the transcription domain (Figure 1B).

In this report we have expanded our study of natural populations of *Drosophila simulans* to obtain better estimates of the range of rDNA locus size and number of R2 in active and inactive individuals. New computer simulations incorporating the transcription domain model for R2 regulation are able to generate stable populations containing rDNA loci with the dynamic properties found in natural populations. Crossover frequency and location, rates of retrotransposition, transcription domain size, and reduction in host fitness are each evaluated for their effects on the final equilibrium between mobile element and host.

## Results

### Range of rDNA locus size and R2 number in natural populations

Correlation of R2 activity with the various properties of an rDNA locus is simplified in *D. simulans* because all rDNA units in this species are located in one locus on the X chromosome [21]. In a previous report [20], R2 transcript levels were determined for 180 lines each containing one rDNA locus from a natural population in San Diego, CA or Atlanta, GA (iso-rDNA lines). Eighteen lines representing the range of R2 transcript levels were then selected to determine the sizes of their rDNA loci and number of R2 copies. No correlation was detected between R2 transcript levels and either rDNA locus size or number of R2 elements. To better define the range of locus size and R2 number in the two populations, these values were determined again for the original 18 lines as well as for an additional 77 randomly chosen lines from the two populations (see Materials and Methods).

**Figure 2:**
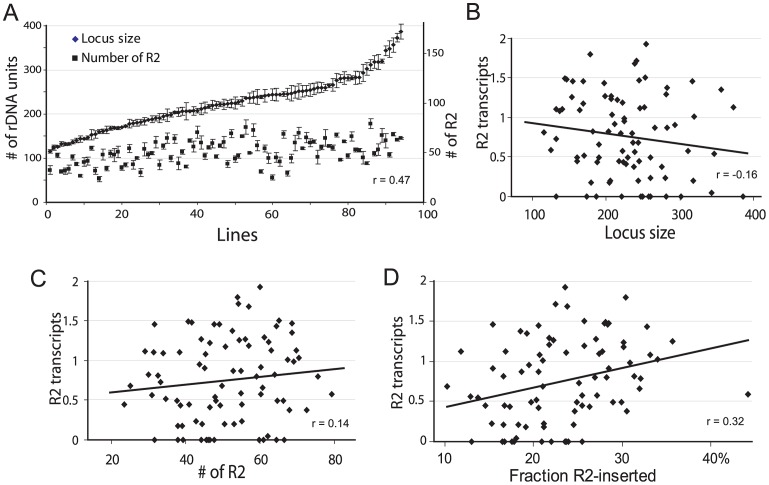
Properties of the rDNA loci derived from natural populations of *D. simulans* and their correlation with the level of R2 transcription. (A) Range of rDNA locus size (diamonds) and R2 number (squares) for 95 iso-rDNA locus lines. The standard errors are shown for the six replicates conducted of each determination (see Materials and Methods). A positive correlation was found between the number of rDNA units (locus size) and the number of R2 (Spearman rank correlation r = 0.47, P = 10^−8^). (B) Using R2 transcript levels previously determined for these same lines [20], no correlation was found between the locus size and the R2 transcript levels (r = −0.16, P = 0.142). (C) No correlation was also found between the number of R2 and the R2 transcript level (r = 0.14, P = 0.187). (D) A small but significant correlation was found between the fraction of the rDNA units inserted with R2 elements and R2 transcript levels (r = 0.32, P = 0.003).

Mean rDNA locus size was found to be 230 units (range 132–373) for the 44 iso-rDNA lines from San Diego and 219 (range 115–386) for the 51 iso-rDNA lines from Atlanta. The mean R2 number was 52 (range 23–70) copies for the San Diego population and 50 (range 31–79) for the Atlanta population. Based on the insignificant difference in the range of values obtained for the two populations (R2 number, P = 0.75; rDNA locus size, P = 0.42, Kolmogorov-Smirnov test) as well as the similar numbers of individuals with detectable levels of R2 transcription in the two populations [20], all subsequent analyses use the combined data sets. The distribution of locus sizes and R2 copy number determined for the 95 iso-rDNA lines are shown in Figure 2A. The number of rDNA units per locus varied over a 3-fold range (115 to 386 units), as did the R2 number (23 to 79 copies). A significant correlation was found between the rDNA locus size and the number of R2 (Spearman rank correlation r = 0.47, P = 10^−8^).

The physical properties of the rDNA locus in the 95 lines were then compared to the level of R2 transcription. A trend towards higher levels of R2 transcripts was associated with smaller rDNA loci (Figure 2B), loci containing more R2 elements (Figure 2C), and loci containing higher fractions of R2-inserted units (Figure 2D). However, there was considerable scatter of transcript levels associated with all ranges of locus size, R2 number and insertion density. These properties of the rDNA locus are thus, not adequate predictors of R2 transcription.

### Frequency of R2 element duplications by recombination

Crossovers between sister chromatids have been suggested to be the major recombinational force at work in the concerted evolution of rDNA loci [1]. In the absence of retrotransposition repeated crossovers in combination with negative selection against inserted units will eventually eliminate R2-inserted units from the rDNA locus [17]. However, in the short term, crossovers can duplicate those R2-inserted units that are located within the offset between the two sister chromatids (Figure S1). It is possible to determine whether individual R2-inserted units have been duplicated by crossovers because many R2s have distinctive 5′ truncations generated during their retrotransposition [12], [13]. Such 5′ truncations are a characteristic property of the target-primed reverse transcription mechanism used by non-LTR retrotransposons [5], [22], [23].

Sensitive PCR assays using one primer upstream of the 28S rDNA insertion site in combination with multiple primers throughout the R2 element have been developed to score all 5′ truncated R2s within individual rDNA loci [12],[13],[20]. By quantifying the signal associated with each PCR band these assays can be used to score whether the individual 5′ truncated elements exist as one, two, three etc. copies in the rDNA locus [20], [24]. Of the 386 R2 5′ truncations present in the 18 original lines representing the range of R2 transcript levels in the *D. simulans* populations [20], 335 (86.8%) were determined to be single-copy, 41 were present in two copies, 9 were present in three copies, and 1 was present in four copies. This infrequent duplication of R2-inserted units has also been found for stocks of *D. melanogaster* and *D. simulans* undergoing long-term propagation in the laboratory [12], [13], [20]. It is also consistent with the low number of trace sequencing reads (equal to the coverage frequency of that genome) for each 5′ truncation found in the genome sequencing projects of *D. simulans* and other Drosophila species [9]. As described in the next section, the infrequent duplication of R2 copies by crossovers represented a critical property that helped to differentiate various models for recombinations within the rDNA locus of *D. simulans*.

### Previous simulation models cannot reproduce the population data

Computer simulations as well as theoretical models have shown that intrachromosomal (between sister chromatids) and interchromosomal (between homologues) crossovers can account for the concerted evolution of tandemly repeated DNA sequences within a locus and between loci in a population [14]–[16]. To aid our studies of the forces that influence the number and stability of R2 we incorporated into these unequal crossover models the presence of active retrotransposons specific to the rDNA loci [17]. The crossovers were located at random uniformly throughout the locus. Because each R2 blocks the function of the inserted rDNA unit, our model assumed individuals with less than a minimum number of uninserted units had reduced fitness (i.e. produced less than the maximum number of offspring), thereby preventing R2 from inserting into all rDNA units. Because little was known of the factors that control R2 activity, our model also assumed that retrotranspositions occurred at a constant low rate in individuals with R2s. Varying the crossover rate, the retrotransposition rate, and the number of uninserted units needed for peak fitness resulted in stable simulated populations with rDNA loci of various mean sizes and levels of inserted units [17].

**Figure 3:**
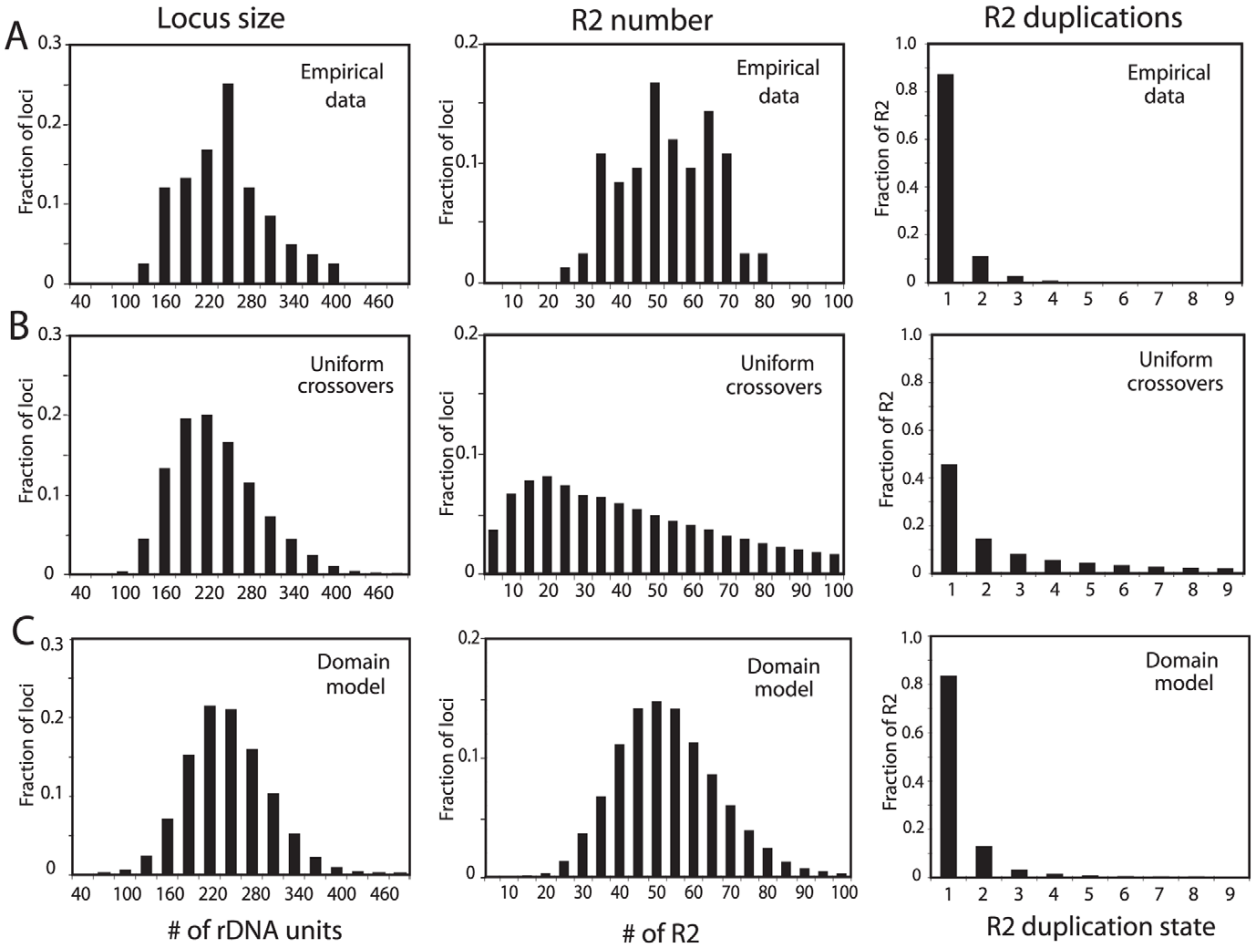
Comparison of the rDNA loci from natural populations with computer simulated loci generated by simple crossover models of concerted evolution. (A) The empirical data determined for rDNA loci from the natural populations in Figure 2 are re-plotted to show the distributions of rDNA locus size (left panel), total R2 number per locus (middle panel), and the number of R2 copies duplicated by crossovers (right panel). The R2 duplication frequency was derived from the approach used in ref. 20 to count the total number of R2 copies in 18 rDNA loci. (B) Simulation data based on the modeling approach described in ref. 17 in which the crossover events are uniformly distributed throughout the rDNA locus. The following parameters were used. Population size = 4000; generations = 10000; replicates = 60; number of uninserted rDNA units required for peak fitness = 100; maximum fecundity = 6; SCE rate = 0.3; ICE rate = 0.0001; crossover offset = 1–8 rDNA units; R2 retrotransposition rate = 0.009 for all loci containing R2 elements; loop deletion rate = 0.00005; deletion size = 1–15 rDNA units. See Materials and Methods for a description of these parameters. How these parameters influence the size of the rDNA locus and number of inserted units can be found in ref. 17. The three panels showing the distributions of locus size, number of R2, and R2 duplication state are shown below the corresponding data from the natural populations. (C) Simulation data based on the transcription domain model for the regulation of R2 elements in a population. The following parameters were used (also described in the Materials and Methods). Population size = 5000; generations = 50000; replicates = 60; transcription domain size = 40; number of uninserted rDNA units in the domain required for peak fitness = 34; maximum fecundity = 6; SCE rate = 0.2 and clustered near the transcription domain with s = 0.05; ICE rate = 0.0001, s = 0.05; crossover offset = 1–11 rDNA units; R2 retrotransposition rate = 0.18 times a square root function of the number of full-length R2 copies in the domain, s = 0.4; loop deletion rate = 0.00007 times the size of the rDNA locus; element induced deletion rate = 0.0065 times the number of full-length R2 copies in the domain; deletion size = 1–30 rDNA units, s = 0.2. The panels containing the distributions of locus size, the number of R2, and R2 duplication state are again shown below the corresponding data from the natural populations.

Simulations using these simple models were extended to allow an analysis of more properties of the loci at equilibrium, in particular the duplication frequency of the R2 elements. Crossover frequencies and retrotransposition rates were readily identified that generated stable populations with mean rDNA locus size (225 units) and R2 number (50) similar to that observed in natural populations of *D. simulans*. However, as shown in Figure 3, while the distribution of locus sizes (number of rDNA units) in these simulations was similar to the empirically derived sizes for the natural populations (Figure 3A and 3B, left panels), two other properties of the simulated loci did not agree with the population data. As shown in the middle panels in Figure 3A and 3B, the simulations generated loci containing less than 20 and over 80 R2-inserted units which are outside the range seen in natural populations. As shown in the right panels of Figure 3A and 3B, the simulated data also did not fit the observed frequencies of R2 duplications for the populations. In the simulations, over 30% of the R2 elements were duplicated by crossovers to a level of four or more copies, while only one example of such high levels of duplications was seen in the natural populations. The narrow range in the number of R2 copies per locus and the infrequent duplications of R2 elements suggested that R2-inserted units are largely excluded from the crossovers within the rDNA loci.

A better fit to the empirical population data could be generated by limiting the locations of the crossovers in the simulations to positions near the center of each rDNA locus (Figure S2). However, what needed to be incorporated into the simulations was a means to regulate R2 activity such that while all individuals in the population contained R2 insertions, only a fraction of the individuals contained transcriptionally active R2 elements. Most important, this R2 activity had to be essentially independent of R2 number or locus size (Figure 2).

### Simulation of an rDNA locus with an active transcription domain

Simulations based on the model that R2 transcription does not occur when there is a large region of the rDNA locus free of R2 insertions, the transcription domain model [18], [20, Figure 1], could reproduce the population data. The incorporation of this model into a simulation program is described in the Materials and Methods and diagrammed in Figure S3. Based on previous estimates of the number of rDNA units needed in *Drosophila* for transcription [4], [25], [26], the size of the transcription domain was varied from 30–70 units. At each generation the middle position of the transcription domain was centered on the largest contiguous block of rDNA units with no R2 insertions. Because R2 transcripts are processed from a 28S rRNA co-transcript [19], in cases where the largest R2-free block was larger than the defined transcription domain, the transcription domain would be “R2 free” and no R2 transcription would occur. However, in cases where the define transcription domain was larger than the largest contiguous R2-free block, the transcription domain would not be R2-free and R2 transcription would occur from those R2 elements within the domain (see Figure 1B). The probability of retrotransposition increased as the number of R2 elements within the domain increased. Because all rDNA units in *D. simulans* are on the X chromosome, the size of each transcription domain on the two X chromosomes of females was set at one half the size of the transcription domain on the single X chromosome of males. Host fitness was determined by the number of uninserted rDNA units available for transcription, as in our previous simulations [17]. In the domain model, however, the number of rDNA units activated for transcription was set at a number somewhat higher than that needed by the host for maximum fitness. Therefore, independent of the total number of inserted units, the presence of only a few inserted units within the activated domain could reduce host fitness. To enable the simulations to duplicate as closely as possible the known properties of R2, half of the retrotransposition events generated 5′ truncated (dead-on-arrival) R2 copies [12], [13]. These truncated copies inactivated rDNA units, played a role in the identification of the transcription domain, and influenced host fitness but could not contribute to the generation of new R2 copies. Finally, because the empirical data suggested that R2 insertions are seldom duplicated by recombination (Figure 3A, right panel), crossovers were localized to various degrees within or near the transcription domain.

Shown in Figure 3C are simulated populations in which the transcription domain was set at 40 units and the crossover rate and R2 retrotransposition frequency were adjusted such that rDNA loci were generated with a mean size of 225 units and a mean number of 50 R2-inserted units. As shown in Figure S4, the final equilibrium was independent of the starting properties of the rDNA loci. The simulated data closely matched the three physical properties of the rDNA loci that were measured in natural populations of *D. simulans*: the distribution of rDNA locus sizes (Kolmogorov-Smirnov test, P = 0.820), the distribution of the number of R2 elements (P = 0.830), and the R2 duplication frequencies (P = 0.497). The clustering of crossovers within the transcription domain would exclude R2 copies from the crossover offset (Figure S1), thus explaining why few R2 copies are duplicated after insertions (Figure 3C, right panel). This minimal participation of R2-inserted units in the crossovers, also explains the more limited range in the number of R2 elements per locus (Figure 3C, middle panel) as the number of R2 elements in each locus was a reflection of the retrotransposition rate and little influenced by crossovers. In these simulations, the region selected for transcription domain, could be anywhere in the rDNA locus, however over time, it was most often located near the middle of the locus (Figure S5A) the region with the fewest R2-insertions (Figure S5B and S5C).

### Additional properties of the rDNA locus consistent with the domain model

**Figure 4:**
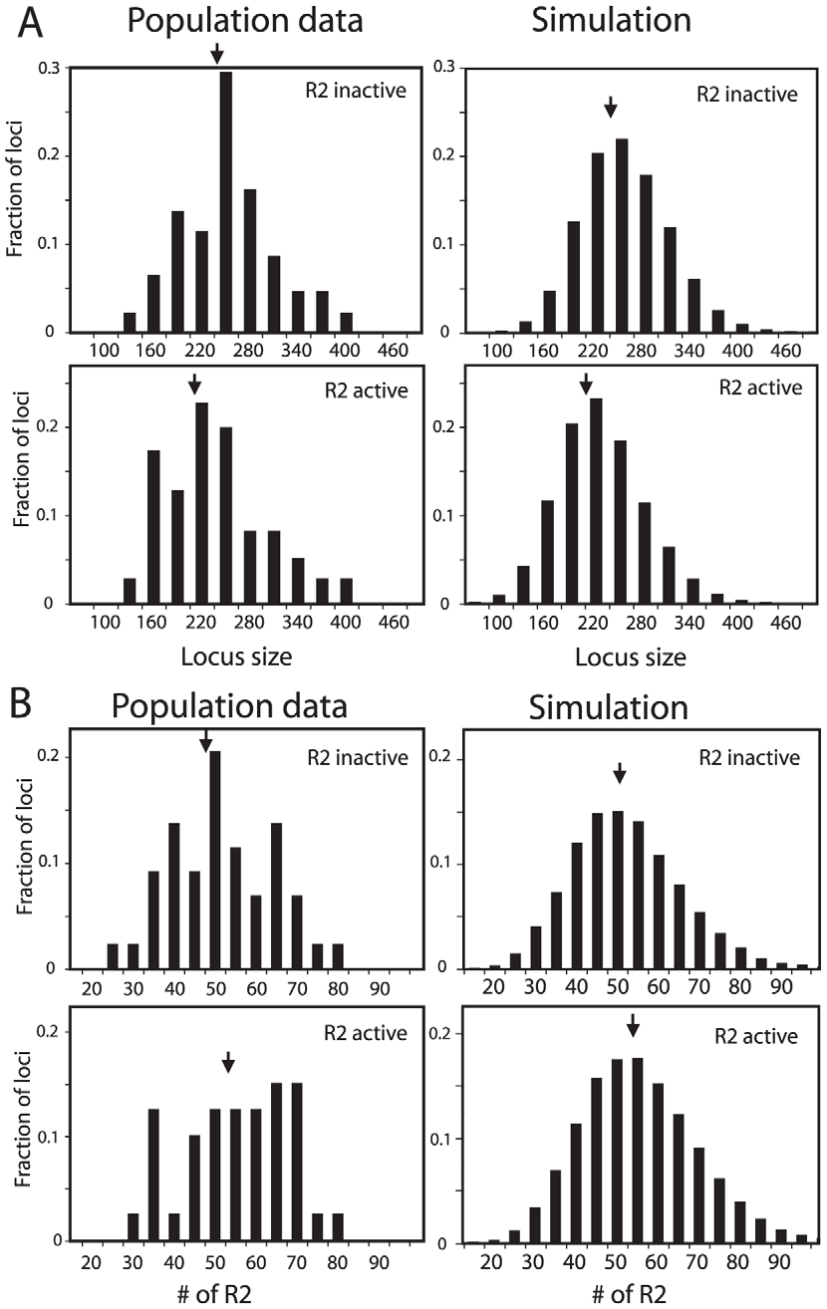
Comparison of the rDNA locus size and R2 number in R2-active and R2-inactive individuals. (A) Distribution of rDNA locus size. Left panels, the *D. simulans* lines shown in Figure 3A were divided into R2-active and R2-inactive pools based on whether full-length R2 transcripts (at least 5 times above background hybridization) had been detected on Northern plots [20]. Right panels, the simulated rDNA loci from Figure 3C were divided into R2-active and R2-inactive pools based on whether a full-length R2 element was present in the transcription domain in the last generation of the simulation. Arrows in all panels indicate mean locus size for the group. The distribution of locus size in the simulated data matched that of the empirical data (Kolmogorov-Smirnov test, P = 0.50 for the R2 active flies, and P = 0.16 for the R2 inactive flies). (B) As in panel A except the distribution of the total number of R2 elements in each locus is plotted for each pool. The distribution of R2 number in the simulated data again closely matched that of the empirical data (K-S test, P = 0.94 for the R2 active flies, and P = 0.99 for the R2 inactive flies).

As an additional means to compare the simulated rDNA loci with the loci from natural populations, R2-active and R2-inactive loci were analyzed separately. About 40% of the lines derived from natural populations of *D. simulans* contained readily detectable levels of R2 transcripts and retrotransposition activity [20]. Plotted in Figure 4A and 4B (left panels) are the distributions of rDNA locus size and R2 number for the R2-active and R2-inactive lines obtained from the populations. As predicted from the trends shown in Figure 2, the R2-active and -inactive flies had nearly the same distributions and mean values (arrows) for both rDNA locus size and number of R2-inserted units. In the domain model simulations used in Figure 3C about 50% of the males and 40% of the females in the populations had at least one full-length R2-inserted rDNA unit in their transcription domain and thus were R2-active. The rDNA locus sizes and R2 numbers for these simulated loci are shown in Figure 4A and 4B, right panels. Paralleling the natural populations, the range in size of the loci and number of R2s were similar for the two groups. Also like the natural populations the simulated R2-active loci were on average slightly smaller and contained a few more R2-inserted units than the R2-inactive loci. The shape of the distribution of R2-inserted units within the locus were similar in the R2-active and inactive loci, but the R2-active loci had on average more R2-inserted units located across the middle of the locus (Figure S5B and S5C).

**Figure 5:**
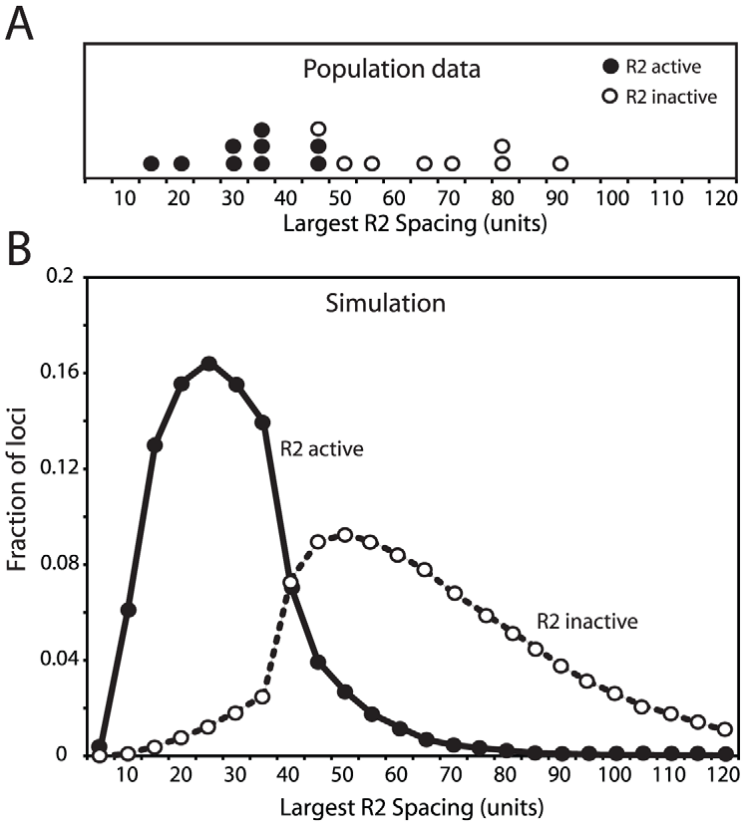
Comparison of the largest region of the rDNA locus free of R2-inserted units in the R2-active and R2-inactive individuals. (A) The empirical data for 17 *D. simulans* lines was determined by pulsed-field gel electrophoresis of *Not*I-digested high molecular weight DNA [18], [20]. The restriction enzyme, *Not*I, cleaves a site in the R2 element but no sites are located within the uninserted rDNA units. An uninserted rDNA unit in *D. simulans* is about 11 kb in length [9]. (B) The largest region of each rDNA locus generated by the domain model simulations is shown. The parameters used in the simulations were identical to those in Figure 3C and Figure 4. In both panel A and B R2-active lines (filled circles) and the R2-inactive lines (open circles) were defined as in Figure 4.

The physical property of the rDNA loci that originally suggested the transcription domain model was the spacing of R2-inserted units within the rDNA locus [18], [20]. This spacing was determined by digesting high molecular weight genomic DNA with the *Not*I restriction enzyme, which cleaves the R2 element but not the rDNA repeat, separating the digested DNA on pulsed-field gels, and probing the gel with a fragment of the rDNA unit. As shown in Figure 5A, this approach revealed the largest contiguous block of uninserted units in nine R2-active lines ranged from 15 to 45 units, while the largest contiguous block of uninserted units in eight R2-inactive lines ranged from 45 to 90 units. Shown in Figure 5B is a plot of the largest blocks of rDNA units free of R2 insertions in the active and inactive rDNA loci from the simulated populations used in Figure 3C and Figure 4. The transcription domain size in these simulations was set at 40 units, explaining the rapid shift from R2-activity to inactivity in those loci with uninserted segments near this size. It should be noted that this boundary is not absolute because of the 5′ truncated R2 elements present in the loci, which cannot give rise to retrotransposition events but can be cleaved by *Not*I, and because R2 activity in females is determined by two rDNA loci. In summary, by localizing both the transcription of the rDNA units and the crossover events to that region of the loci with the lowest level of R2-inserted units, stable populations could be simulated containing rDNA loci with properties consistent with those observed in natural populations of *D. simulans*.

### Effects of individual parameters on rDNA locus dynamics

The transcription domain simulations described to this point were conducted with one set of parameters that reproduced the population data. It should be noted that by making compensating changes other combinations of parameter values were also able to reproduce the population data. To evaluate the effects of each parameter on the structure of the resulting rDNA loci, simulations were conducted in which one parameter of the model was varied while all other parameters were held constant. Four key properties of the loci were recorded at the end of each simulation: 1) the mean rDNA locus size, 2) the mean number of R2 elements, 3) the fraction of the R2 elements that were single copy (i.e. not duplicated by a crossover event), and 4) the fraction of individuals in the population with active R2 elements.

**Figure 6:**
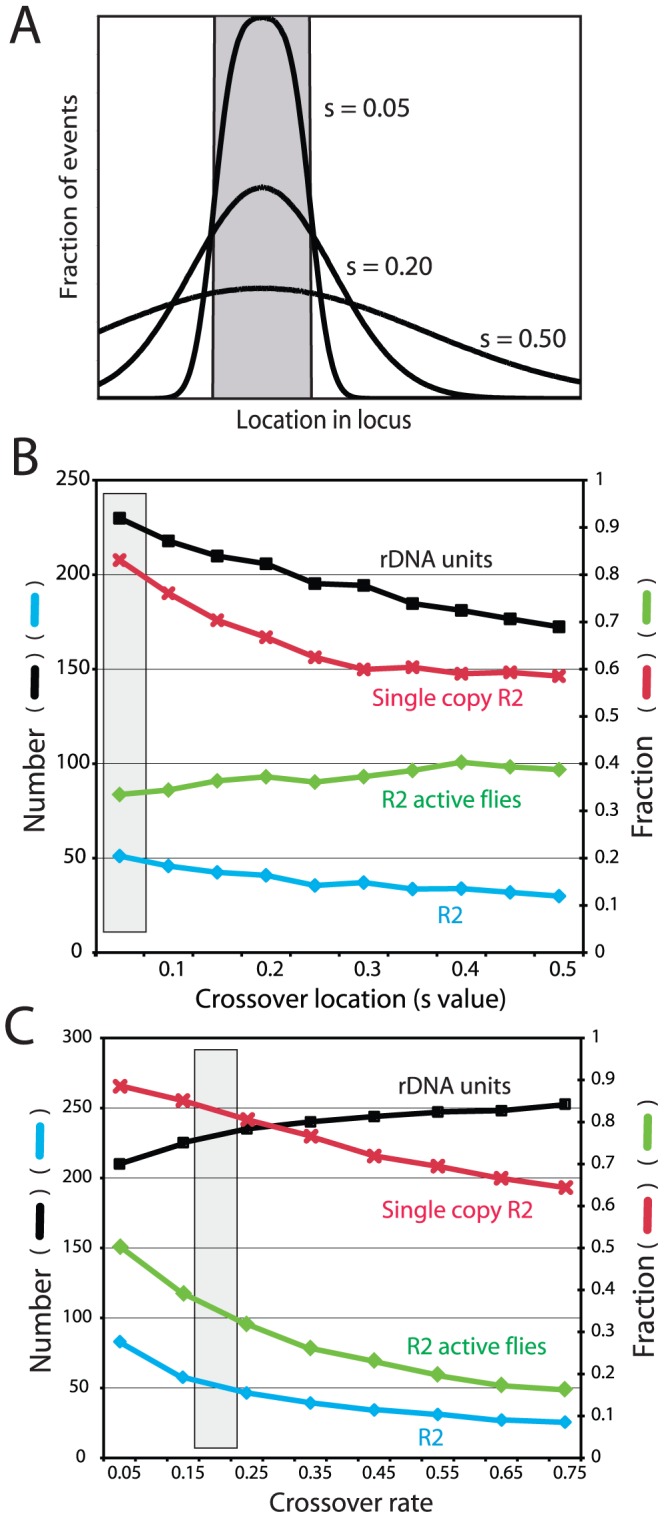
Effects of varying crossover location and crossover frequency on the properties of the simulated rDNA loci. (A) Diagram of how crossovers were localized in the rDNA loci. The gray box represents the transcription domain. For each locus this domain was centered on the region with the fewest R2-inserted units (see Figure 5S for the distribution of domains within the loci). For each simulation crossovers were assigned a standard deviation of location from the domain, S value, ranging from mostly within the transcription domain to more broadly throughout the locus. (B) Simulations in which the distribution of crossover location (S value) was varied while all other parameters were held constant. Four properties of the loci were recorded at the end of each simulation: black symbols, the mean rDNA locus size; blue symbols, the mean number of R2 elements; red symbols, the fraction of the R2 elements that were single copy (i.e. not duplicated by a crossover event); and green symbols, the fraction of individuals in the population with active R2 elements. The gray box represents the parameters used for the simulations in Figure 3, Figure 4 and Figure 5. (C) Simulations in which the frequency of sister chromatid crossovers events was varied. Units are crossovers/loci/generation. All symbols and the gray box are as described in B.

*Crossover location:* As described above, a key property of the rDNA loci assayed from natural populations is that 87% of the R2 elements are single copy (Figure 3A). This property of the loci was replicated in the simulations by clustering the crossover events within the transcription domain. Figure 6A and 6B show in greater detail the effects of varying the crossover location. In each simulation the crossovers were given a normal distribution centered on the transcription domain with the standard deviation of their locations, S, varied from 0.05, in which all crossovers occurred within or near the domain, to 0.5, in which the events were widely distributed across the loci (Figure 6A). As shown in Figure 6B, only limiting most of the crossovers within the transcription domain (low S values) reproduced the infrequent involvement of R2-inserted units in crossover events. For example, with an S value of 0.05, approximately 85% of the R2 insertions remain single copy at the end of the simulation, matching the population data. Higher S values duplicated more R2 elements, but interestingly the total number of R2 insertions decreased, as the more frequent involvement of R2-inserted units in the crossover events increased their rate of elimination from the locus.

#### Crossover frequency and offset size

Two types of crossovers occur in the rDNA locus: between sister chromatids (intrachromosomal) and between chromosomes (interchromosomal). Sister chromatid exchanges have been suggested to be at least two orders of magnitude more frequent than interchromosomal events, and thus the more dominant force in the concerted evolution of the locus [17], [27], [28]. Therefore for our simulations, the interchromosomal rate was held constant at 0.0001 events/chromosome/generation and the intrachromosomal rate was varied from 0.05 to 0.75. As shown in Figure 6C, as the crossover rate increased R2 elements were more rapidly eliminated from the locus, and thus the number of R2 elements decreased. In contrast to the R2 insertions, the higher rate of crossovers increased the total size of the rDNA locus as has been previously shown in previous simulations of tandemly repeated sequences [30]. The mean size of the rDNA locus increases with the crossover frequency, because a wider range of locus size is generated in the population and more smaller loci are eliminated by selection [see Figure 2 in ref 17]. The mean size of the offset between the two chromosomes before the crossover was also varied in the simulations (data not shown). As would be expected, the effect of larger offsets was essentially the same as increasing the crossover frequency. Unfortunately, no empirical estimates of the rate of sister chromatid exchange in the rDNA locus or the size of the offset is available for *Drosophila*.

#### Size of the transcription domain

**Figure 7:**
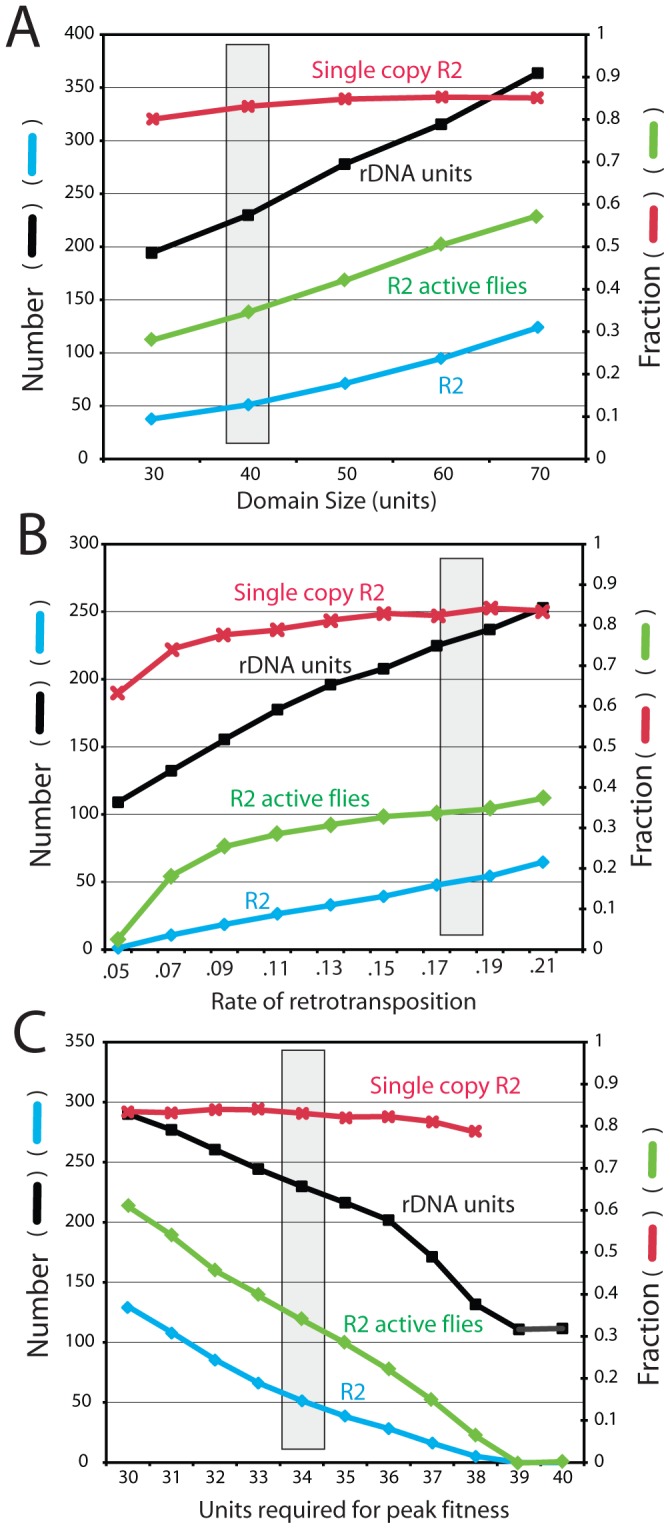
Effects of varying the transcription domain size, retrotransposition rate, and selection against inserted units. The four properties of the rDNA loci are as described in Figure 6. The gray box represents the parameters used for the simulations in Figure 3, Figure 4 and Figure 5. (A) Simulations in which the size of the transcription domain (number of rDNA units transcribed in each individual) was varied. (B) Simulations varying the rate of R2 retrotransposition. The retrotransposition rate was determined for each individual in the population at each generation. This rate was dependent on the square root function of the number of full-length R2 elements present within the transcription domain multiplied by the probabilities shown. Probabilities below 0.05 events per element within the domain per generation could not maintain R2 elements within the populations. (C) Simulations measuring the consequences of varying the number of uninserted units needed within a 40-unit transcription domain to obtain peak fitness. See Materials and Methods for how the extent of fitness reduction was calculated. A requirement of 39 and 40 uninserted units for peak fitness eliminated all R2 elements from the rDNA loci of the populations.

Figure 7A shows the effects of varying the size of the transcription domain from 30 to 70 units. As the domain size increased, the total locus size increased proportionally and remained at five to six times that of the transcription domain. The larger domains gave rise to more R2-active flies in the population and a corresponding increase in the number of R2 elements. Unlike most other parameters in the simulations, where changing the value of one parameter could be counterbalanced by modifications to other parameters, only domain sizes near 40 units could reproduced the size of the largest R2-free regions detected on pulsed field gels (Figure 5). Forty rDNA units are near the minimum values estimated as needed for transcription in *Drosophila* [25], [26].

#### Frequency of retrotransposition

Figure 7B shows the effects of varying the efficiency of the retrotransposition events (i.e. the frequency of a successful R2 retrotransposition when a full-length R2 was present in the transcription domain). As expected, the number of R2-inserted units increased with more efficient retrotranspositions. While the size of the rDNA locus also increased, higher retrotransposition frequencies gave rise to a greater fraction of the units being inserted. Because the rate of R2 insertions in the total population is the product of the probability of retrotransposition times the number of elements in the domain times the fraction of R2-active individuals in the population, the range over which this transposition frequency could be varied was narrow (4 fold). With a measured mean of 50 R2 elements in a locus of 225 units, or ∼22% of the units inserted, the *D. simulans* populations are at the high end of the possible range. Greater frequencies of retrotranspositions produced large loci with most flies containing many active R2s. At the lowest retrotransposition frequencies that could maintain R2, loci were generated with ∼6% inserted rDNA units. Below that value the number of R2-active flies in the population dramatically dropped and R2 was eliminated.

#### Fitness effects

A key assumption in the modeling of R2 maintenance is that each insertion has no effect on host fitness, until a point is reached when there is insufficient number of uninserted rDNA units in the active domain to maintain the appropriate levels of rRNA. As shown in Figure 7C varying the fitness cost associated with R2 insertions had profound effects on the number of R2 and their stability over time. To demonstrate this effect, the simulations were conducted with a domain size of 40 units, and the number of uninserted units needed for peak fitness varied from 40 (i.e. even one inserted unit in the domain resulted in reduced fitness) to 30 units (i.e. 10 inserted units in the domain with no effect on fitness). Fitness reduction was quantified as the number of uninserted units in the transcription domain divided by the number of uninserted units needed for peak fitness. The simulations suggested that only with a transcription domain significantly larger than that needed for peak fitness was the R2 element able to maintain itself in the population. For example, R2 elements were eliminated from the population if one R2 insertion in the domain had no affect, but all subsequent insertions reduced fitness by ∼2.5% (i.e. 39 units were needed for peak fitness). While other means of defining fitness reduction could be devised, these simulations clearly suggest that any significant reduction in fitness associated with a small number of active R2 elements will eliminate the elements. This suggestion of a low fitness effect associated with R2 activity is consistent with our empirical evidence that nearly 40% of the loci found in natural populations support low to moderate levels of R2 activity [20].

While we have modeled the minimal fitness effects of R2 insertion within the transcription domain by assuming the domain is larger than that needed for peak fitness, alternative scenarios are possible. For example, under conditions of suboptimal rRNA levels the cell could increase the size of the transcription domain, or increase the transcription rate of each active unit. Minimal fitness consequences associated with low levels of active R2 elements also suggest that the 28S rRNA fragments after R2 self-cleavage, and/or the potential imbalance in rRNA ratios due to the production of 18S but not 28S rRNA do not cause significant disruption of cellular metabolism.

## Discussion

The long-term relationship between a transposable element and its host involves exceedingly complex interactions that are challenging to address [29], [30]. Indeed, the best understood elements are the DNA transposons that remain active in a species only temporarily and depend upon horizontal transfers to propagate over long periods of time [31], [32]. R2 elements, on the other hand, appear to have co-evolved with their hosts since the origin of most animal taxa [6], [7], [10], [11]. The absolute specificity of R2 for a unique site in the 28S rRNA genes has greatly aided attempts to define three key parameters needed to build a population genetic model for the stability of a mobile element: i) the regulation of element expression, ii) the number of elements and their rates of turnover, and iii) the potential affects of each insertion on the host. In the case of the latter parameter, each insertion blocks the production of intact 28S rRNA from one rDNA unit. R2 element evolution suggests they are simply selfish elements, and are not preserved by the host to aide the regulation or the evolution of the rDNA locus. Many species contain multiple subfamilies of R2, as well as members of other mobile element families inserting into the rRNA genes (e.g. R1 elements). This proliferation of element families and subfamilies is consistent with the selfish propagation of parasites to fill a niche, rather than their maintenance for a useful function [6], [33]–[35]. Even the arguments that mobile elements might provide useful genetic diversity [36], [37] have little application to R2. The sequence of the 28S gene around the insertion site is nearly identical in all eukaryotes, and the expression of the rRNA genes follows a similar pattern in all animals.

In this report we have attempted to integrate into a simple population genetic model all previous findings concerning the structure, regulation and turnover of both R2 elements and the rRNA genes. The critical findings were that *D. simulans* populations could be divided into R2-active and R2-inactive individuals [13], that genetic control over R2 activity mapped to the structure of the rDNA locus itself [18], that the key regulatory step in R2 activity was at the level of transcription [18], and that R2 transcripts are generated by self-cleavage from a 28S rRNA co-transcript [19]. Furthermore, studies to determine why some rDNA loci in natural populations supported R2 transcription while others did not suggested that the size of the rDNA locus and the number of R2 insertions only weakly correlated with R2 activity [20, Figure 2]. Instead, the property of the rDNA locus that best predicted R2 activity was the size of the largest contiguous block of rDNA units free of R2 insertions [18], [20]. These studies of R2 expression could be readily integrated with two previous findings concerning the expression of the rRNA genes themselves. First, that only a small fraction of the rRNA genes are transcribed, an estimated 35 to 50 rDNA units in *Drosophila* [25], [26]. Second, that the rDNA units activated for transcription are contiguous, not individual units distributed throughout the locus [25], [38], [39]. These findings concerning R2 and the rRNA genes gave rise to the transcription domain model, which we have incorporated into computer simulations to model the long-term stability of R2 elements.

Critical to reproducing the population data was the assumption that most crossover events within the rDNA loci were localized within or near the transcription domain. Only then did most R2-inserted units remain single copy (i.e. not duplicated by recombination) consistent with the population data (Figure 3, right panel). This clustering also generated populations with all animals containing a relative narrow range in numbers of R2 (Figure 3, middle panel). The clustering of crossover events in the region with the lowest number of R2-inserted units (the transcription domain) predicts that over many generations the number of uninserted rDNA units within a locus would change more rapidly than the number of R2-inserted units. This is precisely what was observed in our study of the Harwich mutation accumulation lines of *D. melanogaster* [12]. The Harwich lines, originally derived from one inbred stock [40], had been maintained as separate sublines for over 400 generations. During the 400 generations, the size of the rDNA locus on the X chromosomes of the 19 lines changed dramatically shrinking to a low of 140 units in some lines and expanding to over 300 units in others [24]. The vast majority of this variation in number of rDNA units was associated with the uninserted units, with less than 1% of the variation associated with the number of R2-inserted units. There were also no instances within the 400 generations in which an R2-inserted unit was duplicated by recombination [24]. These findings provide strong empirical data to support the model that most crossovers within the rDNA locus occur in regions free of R2 insertions.

The clustering of crossovers within the transcription domain of the rDNA locus could be a result of two non-mutually exclusive mechanisms. First, active RNA transcription may be inducing the crossover events. RNA transcription, or more broadly chromatin structure, has long been associated with various types of genome instability including recombination [41]–[43]. Indeed, one of the first experiments suggesting this connection involved transcription of the rRNA genes [44]. Second, clustering of recombination in the transcription domain may result from the more efficient pairing of this region between chromosomes. The presence of an R2 insertion within a rDNA unit will disrupt its ability to completely align with an uninserted unit. Therefore, if crossovers involve the precise alignment of DNA sequences spanning multiple rDNA units, then those regions most likely to undergo a crossover would be the regions free of insertions. We have conducted computer simulations in which the locations of the crossover events were influenced by the composition of the surrounding rDNA units [45]. When crossovers were permitted only when four contiguous units matched between chromosomes or chromatids (i.e. uninserted matched uninserted unit, and inserted units with inserted units), regions of the locus free of insertions were quickly generated.

It should also be noted that the localization of crossovers to the transcription domain, and thus to regions typically near the center of the locus (Figure S5) does not prevent the concerted evolution of the rDNA genes. Simulations of the rDNA locus involving millions of generations and the addition of low rates of nucleotide substitutions demonstrated that concerted evolution of rDNA units were efficient whether the crossovers were distributed throughout the locus or restricted to the middle of the locus, (Eickbush, M. and Eickbush, T., unpublished). The only aspect of the concerted evolution process that differs between the two models was that only mutations in units near the center of the rDNA locus became fixed when recombinations were restricted to the center of the locus, while variants in units from throughout the locus could become fixed under conditions of uniform crossovers.

An unusual aspect of our population genetic model for the propagation of R2 elements is that R2 activity does not depend upon periodic failure in the host regulatory systems or on low levels of R2 activity that escape host control. The driving force that maintains R2 elements within a population is the recombinations that result in the concerted evolution of the rDNA locus. Because of the stochastic nature of these crossovers, rDNA loci that contain a large region free of R2 insertions and thus have not supported R2 activity for many generations will occasionally undergo crossover events that reduce the R2-free regions to the point that R2-inserted units are transcribed. Loci with active R2 elements will subsequently increase the number of R2 insertions within the same X chromosome, and in females also on the paired X chromosome. Over time, loci with the most active R2 elements will be eliminated from the population by selection. This build-up of R2 elements has been detected in laboratory stocks of *D. simulans* with active R2 elements [13]. While the fitness of stocks with low levels of R2 transcription are similar to that of stocks with no R2 transcription, the fecundity of lab stocks with very high levels of R2 transcription are significantly reduced [D. Eickbush, unpublished data]. The activation of R2 activity by stochastic recombinational forces within the rDNA locus, instead of a reliance on overcoming the host regulatory machinery, may explain why R2 elements are so stable in most lineages of Drosophila, while most mobile elements that insert throughout the genome show a patchy species distribution and extensive variation in abundance [32].

Another possible factor contributing to the long-term stability of R2 is that these elements may be less susceptible to control by the small RNA pathways, notably the *piwi* pathway, which are known to regulate mobile elements [46]. Properties of R2 elements which may contribute to their greater resistance to *piwi* regulation include: a) their strict specificity to the 28S rRNA target means they are unlikely to become part of the piRNA clusters of Drosophila [47], b) their strict orientation within the rDNA unit means antisense RNAs are unlikely to be generated, and c) they are co-transcribed with the highly abundant 28S rRNA, a transcript that simply can not be shut down in any cell type. However, even given these unusual properties of the R2 element, it is interesting to speculate that small RNA pathways are still playing a critical role in R2 regulation by determining what region of the rDNA loci is transcribed. A likely model is that heterochromatin formation induced by small RNA occurs initially on R2 sequences, spreads to the entire rDNA unit, and then into flanking units. As a result the largest region of the rDNA locus free of R2 insertions would be the most likely to remain active for transcription.

Finally, additional support for our model of R2 propagation as well as estimates of the variable parameters used in our simulation could be derived from two sources. First, electron microscopic observations of actively transcribing rDNA loci are needed for *D. simulans*. Previous studies have only been conducted on *D. melanogaster* and more distant species [38], [39]. Direct examination in *D. simulans* would provide better estimates of the number of units within the transcription domain, and in particular whether there is flexibility in the size of this domain in flies where R2 transcripts are detected. Second, more data is needed concerning the frequency of sister chromatid exchanges and the size of the offset. Experiments to estimate these critical parameters are currently underway using pulsed field gels to monitor changes in the spacing of R2 insertion in rDNA locus over time. Finally, more data is needed on the fitness consequences of low levels of R2 expression.

## Materials and Methods

### Fly sources

Ninety-five *D. simulans* lines were chosen from among those initially characterized for R2 transcript levels [20]. Each line contained a single rDNA locus isolated from natural populations in the San Diego and Atlanta areas. These included 18 lines in which the rDNA locus had been characterized in the original study, as well as another 77 randomly chosen lines. Stock Dm2057 was obtained from the Bloomington Stock Center.

### Slot-blot analysis and DNA hybridization

Genomic DNA was extracted from 30 adult females of each line. Approximately 10 µg of genomic DNA was denatured for 10 min in 0.25 M NaOH, 0.5 M NaCl and diluted on ice to 600 µl in 0.1× SSC, 0.125 M NaOH. The DNA samples were loaded in triplicate onto a nylon membrane (PerkinElmer GeneScreen plus), presoaked in 0.4 M Tris-HCl, pH 7.5 using a Slot-blot apparatus (Schleicher and Schuell). The DNA was drawn onto the membrane with a gentle vacuum for 30 seconds, the membrane was removed and neutralized in a solution of 0.5 M NaCl, 0.5 M Tris-HCl pH 7.5, air dried, and baked at 80°C in a vacuum for 2 hours. A 5.5 kb *Xba*I fragment from the alcohol dehydrogenase (*Adh*) gene was gel purified from plasmid pXba [48], ^32^P-labeled by random priming and hybridized to the slot-blot membranes under conditions previously described [18]. After hybridization the signals were monitored in a Bio-Rad Personal Molecular Imager and the level of hybridization quantified by Quantity-One software (Bio-Rad). The *Adh* probe was striped from the membrane by boiling for 30 min in a solution of 0.015 M sodium chloride, 0.0015 M sodium citrate, 1% SDS. The membrane was then reprobed with a 630 bp DNA fragment of 18S rRNA gene synthesized by PCR with a forward primer located at position 280 of the 18S gene, 5′-GTCTTGTACCGACGACAGATC-3′ and a reverse primer located at position 910, 5′-CAGAACAGAGGTCTTATTTC-3′. Signals were quantified as above and the membranes stripped of probe. A 300 bp R2 probe corresponding to the 3′ end of the element [20] was used to determine R2 number in each line.

On each membrane, control stocks A179 (*D. simulans*) and Dm2057 (*D. melanogaster*) were blotted in triplicate as quantization references. The locus size and R2 numbers for A179 had been previously determined [20]. Dm2057 is the sequenced *D. melanogaster* reference strain [49]. Southern blotting, PCR analysis, and the analysis of the original trace sequences were previously used to determine the composition of the rDNA locus and R2 elements in this stock [9, J. Zhou and W. Burke, unpublished data]. To estimate the rDNA locus size and R2 number, the ratios of hybridization signal from the18S and R2 probes to the *Adh* probe for the two control lines were used to standardize the ratio from each of the unknown lines. The values presented in Figure 2A represent the mean and standard errors of six slots. To determine the accuracy of this approach we compared the values determined by this hybridization approach with that used for 18 lines in our original study [20]. The number of R2 copies estimated by the current hybridization approach differed by less than 9% from the previous determinations, while the total number of rDNA units differed by less than 10%. In addition, the relative ranks among the 18 lines determined by the two methods agreed except for minor shifting of lines with similar sized loci.

### Computer simulations of the rDNA locus

A new version of the computer program described in reference 17 was implemented again in C to specifically duplicate the R2 and rDNA dynamics in populations, like that of *D. simulans*, where all rDNA units are located on the X chromosome. A flow chart summarizing each step of the simulations can be found in Figure S3 and the complete program in Text S1. The final equilibrium in the population was not dependent upon the initial starting conditions (Figure S4), thus each simulation was begun with a population of rDNA loci that were 250 units in length with 20% of the units selected at random to contain an R2 element. As shown in Figure S6 the final equilibrium was also not dependent upon the size of the simulated population over the range of 2,000 to 1,000,000 individuals. To reduce computer time, all simulations were conducted with populations of 5,000 individuals for 50,000 generations. Most simulations came to equilibrium after 5000 generations for all parameters discussed in this report, but the simulations were extended to 50,000 generations to insure stability. Because the simulations were based on the stochastic process of recombination and the selection of progeny for the next generation, the populations experienced significant genetic drift. Therefore, unless otherwise indicated for each set of parameters the simulations were repeated 50 times with the results averaged. Descriptions of the individual parameters are as follows.

#### Location of the transcription domain

The number of rDNA units activated for transcription, the transcription domain, was the same for all individuals in the populations and was varied in the simulations from 30 to 70 units (Figure 7A). The number of rDNA units required to supply rRNA in *Drosophila* has been estimated to be 35–50 units [4], [25], [26]. In each locus at each generation the middle position of the transcription domain was centered in the largest contiguous block of rDNA units with no R2 insertions. In individuals where the largest R2-free block was larger than the defined transcription domain, the transcription domain would be “R2 free” and no R2 transcription would occur. However, in cases where the define transcription domain was larger than the largest contiguous R2-free block, the transcription domain would contain R2 inserted units resulting in R2 transcription and retrotransposition. Because females can utilize the rDNA loci on both their X chromosomes [18], the size of the transcription domain on each chromosome in females was one-half that of the single chromosome in males. At equilibrium most transcription domains were located near the center of the rDNA locus (Figure S5).

#### Individual fitness

Fitness was applied as the number of chromosomes (gametes) contributed to the next generation. Because it is not known how or whether rRNA transcripts levels can be adjusted or compensated for, we simply set fitness as a linear function of the total number of units needed. In other words, the number of gametes generated by each individual was calculated as the number of uninserted units in the transcription domain divided by the number needed for peak fitness times the number of gametes produced at peak fitness. Initial simulations with individuals containing peak fitness contributing 6 or 18 gametes to the pool of chromosomes available for the next generation gave nearly identical results (data not shown). Again to reduce computational time, for all experiments reported here, peak fitness resulted in 6 chromosomes. The number of uninserted units needed for peak fitness ranged from 0–10 units fewer than the total domain size. From the pool of available chromosomes at each generation (approaching 30,000 chromosomes), 7,500 chromosomes were randomly selected to generate the 2,500 females and 2,500 males present in the next generation.

#### Recombination

To simulate crossover events two rDNA arrays were aligned at their transcription domains, shifted relative to each other by an offset randomly determined between one unit and a maximum number, which was varied over the range 8 to 30. The two arrays were then cut at a random location in the overlap region and the ends combined. Crossovers were of two types. Sister chromatid exchanges (SCE or intrachromosomal) involved two identical copies of the rDNA locus (simulating a post-DNA replication stage). Interchromosomal exchanges (ICE) involved exchanges between the two X rDNA loci in females. The ICE rate has been estimated at 0.0001 events per chromosome per generation [17], [27], [28] and was not varied. The SCE rate has been suggested to be much higher than the ICE rate and was varied over the range 0.05 to 0.75 events per chromosome per generation.

#### Retrotransposition

In Drosophila, nucleotide sequence identities among R2 copies are greater than 99.5%, with virtually all full-length copies containing an intact open reading frame [8], [9], [50]. Thus the simulations assumed that all full-length R2 elements within the transcription domain could give rise to new insertions (i.e. autonomous elements). However, because in *D. simulans* about 50% of the R2 elements undergo 5′ truncations during their insertion and are not functional [13], [20], 50% of the simulated retrotransposition events generated “dead-on-arrival” copies. These dead copies were included in the determination of the location of the transcription domain and estimates of fitness reduction, but not in the determination of the retrotransposition rates. The rate of R2 retrotransposition was determined for each individual in the population at each generation. The rate was determined as the square root function of the number of full-length elements in the transcription domain multiplied by a probability that was varied. Each newly generated R2 in the simulations was labeled to allow determination at the end of the simulation as to whether it had been duplicated by crossovers.

#### Loop-deletion

Without a mechanism to select against individuals with large loci, the size of the rDNA locus slowly increases during any simulation of crossovers within the rDNA locus [16], [17]. As in previous simulations, to prevent this increase, low levels of loop-deletions (crossovers between two locations on the same chromosome) were included. The rate of loop-deletion was varied from 0.00005–0.00007 events/chromosome/generation times the number of rDNA units in the locus. Because the rate of R2 deletions in *D. simulans* has been shown to increase with R2 activity [51], an additional deletion rate adjustment (0.0065) was based on a linear function of the number of full-length elements within the transcription domain. The deletion size varied linearly between 1 and 30 units.

#### Recombination location

The location within the rDNA locus of the crossovers events, new R2 insertions and loop deletions were distributed to various degrees with respect to the transcription domain. This distribution was varied over the range from mostly within the domain (standard deviation of the location, S = 0.05), to more widely distributed throughout the loci (S = 0.5) (see Figure 6A). The supporting biological arguments and the importance of clustering crossover events in the transcription domain are described in the text. Because comparable arguments cannot be made for loop deletions and retrotransposition events, and the effects of their clustering was less pronounced, the following values where used for all simulations reported here; loop-deletions, S = 0.2; retrotranspositions, S = 0.4. Localizing these events to greater degrees within the transcription domain increased the number of R2 elements within the rDNA loci, because new R2-active chromosomes were more rapidly produced in the population.

## Supporting Information

Figure S1 Diagram showing how crossovers within the rDNA loci can change the size of the locus and eliminate/duplicate specific copies of R2 elements. A small region of an rDNA locus is diagramed with individual R2 elements indentified (orange boxes) within rDNA units (black boxes). Two identical rDNA loci (i.e. sister chromatids) are aligned but offset by three rDNA units. A crossover between uninserted rDNA units located to either side of element c (dotted lines) results in recombinants in which one locus is three units shorter and missing element c, and one locus is three units longer with two copies of element c.
(EPS)

Figure S2 Comparison of rDNA loci from natural populations with simulated loci generated by limiting crossovers to the center of the rDNA locus. (A) Empirical data determined for rDNA loci from the populations in Figure 2 are re-plotted to show the distributions of rDNA locus size (left panel), total R2 number per locus (middle panel), and the number of instances R2 copies had been duplicated by crossovers (right panel). These plots are identical to those in Figure 3A. (B) Simulation data based on the modeling approach described in ref. 17, and utilize the same parameters as in Figure 3B, except that all crossover events are clustered at the center of the rDNA locus. The three panels showing the distributions of locus size, number of R2, and R2 duplication state are shown below the corresponding data from the natural populations.
(EPS)

Figure S3 A flow chart of the computer program used for the transcription domain model simulations described in this report.
(EPS)

Figure S4 Data showing that the final equilibrium of rDNA structures reached for the simulated populations was independent of the starting properties of the rDNA loci. The mean rDNA locus size (top three lines) and R2-inserted units (bottom three lines) for the populations in the first 6000 generations of the simulation are shown for different starting conditions: blue traces (large initial loci with many R2s), red traces (smaller loci with fewer R2s) and green traces (loci with only one R2 insertion). R2-inserted units were randomly distributed in the starting rDNA loci. Because individual simulations show large stochastic changes, the traces represent the mean values of 50 replicates. All parameter values used in these simulations are the same as those used in Figure 3C, Figure 4 and Figure 5. Lower final mean values for the simulations initiated with only one R2 copy per locus (green) resulted from the loss of all R2 elements from the population in 11% of the simulations. Those populations that retained R2 had the same mean values for locus size and R2 number as in the other two simulations.
(EPS)

Figure S5 Location of the rDNA transcription domain and distribution of R2 elements in the simulations involving the transcription domain model. The simulation parameters are the same as that shown in Figure 3C, Figure 4 and Figure 5. A. Location of the transcription domain. At the end of the simulations each rDNA loci was divided into 20 equal-sized segments, and the fraction of the transcription domains in the population that were located in each segment was plotted. B. Distribution of R2 elements across the rDNA locus in the R2-inactive loci. The mean fraction of the rDNA units inserted with R2 for each of the 20 equal-sized segments of the rDNA loci has been plotted. C. Distribution of R2 elements across the locus in the R2-active loci. The data plotted is as in B. The central one-third of the R2-active loci have about a 60% higher mean frequency of R2 insertions than the R2-inactive loci.
(EPS)

Figure S6 Data showing that the final equilibrium reached in the simulations was not highly sensitive to the size of the populations. The simulation parameters are the same as that shown in Figure 3C, Figure 4 and Figure 5. The same four properties of the loci that were shown in Figure 6 and Figure 7 are also shown here: black symbols, the mean rDNA locus size; blue symbols, the mean number of R2 elements; red symbols, the fraction of the R2 elements that were single copy (i.e. not duplicated by a crossover event); and green symbols, the fraction of individuals in the population with active R2 elements. For most population sizes the average values and standard errors presented were based on 50 simulations. In the case of 2,000 individual population, 100 simulations were conducted. Because of the time associated with each simulation, in the case of the 200,000 individual and 1,000,000 individual populations only 15 simulations and 2 simulations, respectively, were conducted. The standard error among the simulations was associated with random genetic drift and therefore decreased dramatically with population size.
(EPS)

Text S1 A text file of the computer program, written in C, that was used in this report to simulate the R2 elements and the rDNA loci in populations of *D. simulans*. The random number generator available at http://fmg-www.cs.ucla.edu/geoff/mtwist.html was used in all simulations.
(DOCX)
