# Supplementary material for: A Population Genetic Model for the Maintenance of R2 Retrotransposons in rRNA Gene Loci
Source: PLoS Genet. 2013 Jan 10;9(1):e1003179. doi: 10.1371/journal.pgen.1003179 (PMC3542110; doi:10.1371/journal.pgen.1003179)
Supplement: Text S1 — A text file of the computer program, written in C, that was used in this report to simulate the R2 elements and the rDNA loci in populations of D. simulans. The random number generator available at http://fmg-www.cs.ucla.edu/geoff/mtwist.html was used in all simulations. (DOCX) [file pgen.1003179.s007.docx]

#include <stdio.h>

#include <time.h>

#include <stdlib.h>

#include <string.h>

#include <math.h>

#include "mtwist.c"

#include "randistrs.c"

// Simulation length variables

#define Npop 1000000 // fly population size, i.e. # of flies (half females, half males)

const int Ngen = 50000; // number of generations to run for

const int Nreplicate = 1; // number of times to repeat the simulation (all data averaged/collected in additional seperate files

// Initial locus variables

#define initialLocusSize_X 250 // initial size of all X loci

const int Y_present = 0; // flag for whether there is a Y locus

#define initialLocusSize_Y 211 // initial size of all Y loci

const double initialR2level_X = 0.2; // fraction of X locus units initially inserted with R2

const double initialR1level_X = 0.1; // fraction of X locus units initially inserted with R1

const double initialR2level_Y = 0.0; // fraction of Y locus units initially inserted with R2

const double initialR1level_Y = 0.0; // fraction of Y locus units initially inserted with R1

const int single_ancester = 0; // flag for whether all ancestral loci are the same (different X and Y loci)

const int ancester_random = 0; // flag for whether ancestral loci have element insertions in a random distribution or a user defined distribution using the following two arrays

const float R1dist[20]={0.190,0.087,0.059,0.045,0.035,0.026,0.018,0.015,0.013,0.012,0.012,0.013,0.015,0.018,0.026,0.035,0.045,0.059,0.087,0.190};

const float R2dist[20]={0.190,0.087,0.059,0.045,0.035,0.026,0.018,0.015,0.013,0.012,0.012,0.013,0.015,0.018,0.026,0.035,0.045,0.059,0.087,0.190};

// Fly viability variables

const int transcription_flag = 1; // flag for flies transcribing a small region of the locus (if 0, then all units are transcribed)

const int transcription_size = 40; // number of total units transcribed in a fly if transcription_flag is 1 (half from each locus)

const int transcription_stoc = 0; // flag for whether stochastic processes occur during the determination of the transcribed position

const float trStoc_normal_s = 0.2; // the distribution of transcribed region starting positions around the original position selected (1.5 = random)

const int Y_dominance = 0; // in males, flag for whether the Y locus is dominant (-1=Y-recessive, 0=codominance, 1=Y-dominant)

const int nuc_dominance_flag = 0; // flag for nuclear dominance (only used in males if Y_dominance is 0)

const int flag_selection = 1; // flag for natural selection (if 0, all flies produce the maximum number of offspring)

const int w1_f = 10; // minimum number of uninserted transcribed units needed in females for the chance to produce offspring

const int w2_f = 34; // minimum number of uninserted transcribed units needed in females to guarantee the maximum number of offspring

const int w1_m = 10; // minimum number of uninserted transcribed units needed in males for the chance to produce offspring

const int w2_m = 34; // minimum number of uninserted transcribed units needed in males to guarantee the maximum number of offspring

const double act_fitPen = 0.0; // fitness penalty if fly has an active element

#define fecundity 6 // the maximum number of offspring loci each individual fly can produce

// Fly element 'memory' variables

const int memory = 10; // the number of generations a locus can recognize an element

const int memDelay = 0; // the number of generations a locus can't see an element beyond the initial generation

const double retro_multiplier = 1.0; // the increase in retrotransposition rate in the initial generation a locus can no longer see an element

// R2-retrotransposition variables

const int R2_present = 1; // flag for the presense of R2-elements in the population

const int transcribed_R2_retro = 1; // flag for R2-retrotransposition occuring exclusively in the transcribed region

const int R2_retro_stoc = 1; // flag for whether there are stochastic processes during R2-retrotransposition

const float R2_retro_stoc_normal_s = 0.4; // the level the locus is off due to stochastic processes during R2-retrotransposition (1.5 = random)

const float trR2_retro_rate_f_X = 0.18; // R2-retrotransposition rate for transcribed elements in X loci in female flies

const float trR2_retro_rate_m_X = 0.18; // R2-retrotransposition rate for transcribed elements in X loci in male flies

const float trR2_retro_rate_Y = 0.0; // R2-retrotransposition rate for transcribed elements in Y loci

const float R2_retro_rate_f_X = 0.0; // R2-retrotransposition rate in X loci in female flies

const float R2_retro_rate_m_X = 0.0; // R2-retrotransposition rate in X loci in male flies

const float R2_retro_rate_Y = 0.0; // R2-retrotransposition rate in Y loci

const double trR2_retro_func = 0.5; // function used to base R2-retrotransposition rate on the number of transcribed R2-inserted units (0=constant rate, 1=linear rate, 2=quadratic rate, 3=cubic rate, etc.)

const double R2_retro_func = 0.5; // function used to base R2-retrotransposition rate on the number of R2-inserted units (0=constant rate, 1=linear rate, 2=quadratic rate, 3=cubic rate, etc.)

const double retro_without_R2 = 0.0; // R2-retrotransposition rate when no elements are present

const double R2_loop_rate = 0.0065; // rate at which R2 transcription causes loop deletion

const double R2_loop_func = 1; // function used to determine rate of loop deletion caused by R2 transcription

const int R2_picky = 0; // flag for whether R2-elements can insert into R1-inserted units

const float R2_trunc_prob = 0.5; // rate in which new R2-insertions result in a truncated element

const int R2_trunc_num = 360; // number of different truncated R2-elements

const double R2_trunc_cut_frac = 0.5; // fraction of truncated R2-elements that can still be cut

#define last_R2_cut (int)(R2_trunc_num*R2_trunc_cut_frac) // last R2 truncation number that can still cut (all smaller numbers will cut)

// R1-retrotransposition variables

const int R1_present = 0; // flag for the presense of R1-elements in the population

const int transcribed_R1_retro = 1; // flag for R1-retrotransposition occuring exclusively in the transcribed region

const int R1_retro_stoc = 1; // flag for whether there are stochastic processes during R1-retrotransposition

const float R1_retro_stoc_normal_s = 0.2; // the level the locus is off due to stochastic processes during R1-retrotransposition (1.5 = random)

const float trR1_retro_rate_f_X = 0.09; // R1-retrotransposition rate for transcribed elements in X loci in female flies

const float trR1_retro_rate_m_X = 0.09; // R1-retrotransposition rate for transcribed elements in X loci in male flies

const float trR1_retro_rate_Y = 0.0; // R1-retrotransposition rate for transcribed elements in Y loci

const float R1_retro_rate_f_X = 0.0; // R1-retrotransposition rate in X loci in female flies

const float R1_retro_rate_m_X = 0.0; // R1-retrotransposition rate in X loci in male flies

const float R1_retro_rate_Y = 0.0; // R1-retrotransposition rate in Y loci

const double trR1_retro_func = 0.5; // function used to base R1-retrotransposition rate on the number of transcribed R1-inserted units (0=constant rate, 1=linear rate, 2=quadratic rate, 3=cubic rate, etc.)

const double R1_retro_func = 0.5; // function used to base R1-retrotransposition rate on the number of R1-inserted units (0=constant rate, 1=linear rate, 2=quadratic rate, 3=cubic rate, etc.)

const double retro_without_R1 = 0.0; // R1-retrotransposition rate when no elements are present

const double R1_loop_rate = 0.0065; // rate at which R1 transcription causes loop deletion

const double R1_loop_func = 1; // function used to determine rate of loop deletion caused by R1 transcription

const int R1_picky = 0; // flag for whether R1-elements can insert into R2-inserted units

const float R1_trunc_prob = 0.5; // rate in which new R1-insertions result in a truncated element

const int R1_trunc_num = 500; // number of different truncated R1-elements

const double R1_trunc_cut_frac = 0.5; // fraction of truncated R1-elements that can still be cut

#define last_R1_cut (int)(R1_trunc_num*R1_trunc_cut_frac) // last R1 truncation number that can still cut (all smaller numbers will cut)

// Loop-deletion variables

const int transcribed_loop = 1; // flag for loop-deletion occuring exclusively in the transcribed region

const int loop_stoc = 1; // flag for whether there are stochastic processes during loop-deletion

const float loop_stoc_normal_s = 0.25; // the level the locus is off due to stochastic processes during loop-deletion (1.5 = random)

const double loop_rate = 0.00007; // rate at which loop-deletion occurs (if transcribed_loop is 1 then the rate is constant)

const double loop_func = 1; // function used to base loop-deletion rate off of locus size, not used if transcribed_loop is 1 (0=constant rate, 1=linear rate, 2=quadratic rate, 3=cubic rate, etc.)

const int loop_size = 30; // maximum size that can be deleted (the actual range of values is 1-loop_size)

// SCE-recombination variables

const int transcribed_SCE_recomb = 1; // flag for SCE occuring exclusively in the transcribed region

const int SCE_stoc = 1; // flag for whether there are stochastic processes during SCE

const float SCE_stoc_normal_s = 0.05; // the level the locus is off due to stochastic processes during SCE (1.5 = random)

const int matching_SCE_recomb = 0; // flag for SCE occuring only in positions where the loci match each other (currently not implemented)

const int required_matching_SCE = 0; // number of units that need to match to qualify the position for SCE (currently not implemented)

const float SCE_rate_f_X = 0.2; // SCE rate in X loci in female flies

const float SCE_rate_m_X = 0.2; // SCE rate in X loci in male flies

const float SCE_rate_Y = 0.0; // SCE rate in Y loci

const int SCE_random = 1; // flag to pick the recombination location using a random distribution, if 1, or a normal (clustered) distribution, if 0

const float SCE_normal_s = 0.1; // the level of clustering used in the normal distribution (lower numbers are more clustered)

const int SCE_stagger = 11; // the maximum number of units the loci are staggered before recombination (the actual range of values is 1-SCE_stagger)

// ICE-recombination variables

const int transcribed_ICE_recomb = 1; // flag for ICE occuring exclusively in the transcribed region

const int ICE_stoc = 1; // flag for whether there are stochastic processes during ICE

const float ICE_stoc_normal_s = 0.05; // the level the locus is off due to stochastic processes during ICE (1.5 = random)

const int matching_ICE_recomb = 0; // flag for ICE occuring only in positions where the loci match each other (currently not implemented)

const int required_matching_ICE = 0; // number of units that need to match to qualify the position for ICE (currently not implemented)

const float ICE_rate_XX = 0.0001; // ICE rate between the X loci in female flies

const float ICE_rate_XY = 0.0; // ICE rate between the X and Y loci in male flies

const int ICE_random = 1; // flag to pick the recombination location using a random distribution, if 1, or a normal (clustered) distribution, if 0

const float ICE_normal_s = 0.1; // the level of clustering used in the normal distribution (lower numbers are more clustered)

const int ICE_stagger = 11; // the maximum number of units the loci are staggered before recombination (the actual range of values is 1-ICE_stagger)

// Other

#define size_max 2000 // maximum number of units in a locus

#define unitSize (1+(R2_present*2)+(R1_present*2)) // the number of characters used to represent a single unit

const float normal_s = 0.1;

char alphabet[] = "ABCDEFGHIJKLMNOPQRSTUVWXYZ"; // char used to label different trauncated elements

// Data starage

int R1sldwin_X[20], R2sldwin_X[20], UNsldwin_X[20], TRsldwin_X[20];

int R1sldwin_X_act[20], R2sldwin_X_act[20], UNsldwin_X_act[20], TRsldwin_X_act[20];

int R1sldwin_X_inact[20], R2sldwin_X_inact[20], UNsldwin_X_inact[20], TRsldwin_X_inact[20];

int R1sldwin_Y[20], R2sldwin_Y[20], UNsldwin_Y[20], TRsldwin_Y[20];

int R1sldwin_Y_act[20], R2sldwin_Y_act[20], UNsldwin_Y_act[20], TRsldwin_Y_act[20];

int R1sldwin_Y_inact[20], R2sldwin_Y_inact[20], UNsldwin_Y_inact[20], TRsldwin_Y_inact[20];

int R1_trunc_max_X=0, R2_trunc_max_X=0;

int R1_trunc_copy_X[size_max+1];

int R2_trunc_copy_X[size_max+1];

int R1_trunc_copy_X_act[size_max+1];

int R2_trunc_copy_X_act[size_max+1];

int R1_trunc_copy_X_inact[size_max+1];

int R2_trunc_copy_X_inact[size_max+1];

int R1_trunc_max_Y=0, R2_trunc_max_Y=0;

int R1_trunc_copy_Y[size_max+1];

int R2_trunc_copy_Y[size_max+1];

int R1_trunc_copy_Y_act[size_max+1];

int R2_trunc_copy_Y_act[size_max+1];

int R1_trunc_copy_Y_inact[size_max+1];

int R2_trunc_copy_Y_inact[size_max+1];

int canceledR2retro, canceledR1retro, canceledSCE, canceledICE;

int total_active_X, total_R2_active_X, total_R2_active_X_m, total_R1_active_X, total_R1_active_X_m;

int total_inactive_X, total_R2_inactive_X, total_R2_inactive_X_m, total_R1_inactive_X, total_R1_inactive_X_m;

int total_active_Y, total_R2_active_Y, total_R1_active_Y;

int total_inactive_Y, total_R2_inactive_Y, total_R1_inactive_Y;

int R2_frag_size[5], R1_frag_size[5];

// Function declarations

void newLocus(char*,int,double,double); // generates a new locus based on the given varaiables

int countUn(char*, int); // counts the number of uninserted units in a locus/transcribed region

int countR2(char*, int, int); // counts the total number of R2-inserted units in a locus/transcribed region (from both single and double inserted units)

int countR1(char*, int, int); // counts the total number of R2-inserted units in a locus/transcribed region (from both single and double inserted units)

int countDouble(char*, int); // counts the total number of double-inserted units in a locus/transcribed region

int R2transposition(char*); // performs an R2-retrotransposition event on the given locus

int R1transposition(char*); // performs an R1-retrotransposition event on the given locus

void loopDeletion(char*); // performs an loop-deletion event on the given locus

void sischr(char*); // performs an SCE recombination event on the given locus

void interce(char*,char*); // performs an ICe recombination between the given loci

void transcribeNone(char*); // makes it so none of the units in the given locus are transcribed

void transcribeAll(char*); // makes it so all of the units in the given locus are transcribed

void transcribeArea(char*,int,int,int); // makes it so a limited region of given size in the given locus is transcribed

void nuclearDominance(char*,char*); // adjusts the size of the transcribed region in the given loci to minimize the number of transcribed elements

int transcriptionStart(char*,int); // finds the start of the transcribed region in the given loci

int transcriptionLength(char*,int); // finds the length of the transcribed region in the given loci

int uninsertedUnit(char*,int); // determines if given unit is uninserted

int R2insertedUnit(char*,int,int); // determines if given unit is R2-inserted

int R1insertedUnit(char*,int,int); // determines if given unit is R1-inserted

int doubleInsertedUnit(char*,int); // determines if given unit is double-inserted

int numOffspringF(int,int,int); // determines the number of offspring loci from a female fly

int numOffspringM(int,int,int); // determines the number of offspring loci from a male fly

void sldwin(char*,char,int,int); // perform sliding window analysis on the given locus

void trunc_abundance(char*,char,int,int); // determines truncated element abundance in the given locus

void fragment_size(char*); // determines fragment size between elements

double prob(void); // returns a random double in the range 0≤X<1

double Normal(double); // returns a random double in the range 0≤X<1 biased towards 0.5

int rStagger(int); // determines the number of units to stagger the loci during recombination

int trunc_num(char, char); // determines the truncation profile number based on the identifying characters

int main(void){

time_t t1, t2, t3;

int time_diff;

t1 = time(NULL);

printf("\nSimulation started at %s", ctime(&t1)); fflush(stdout);

mt_seed();

int c = 0;

char ancester[unitSize*size_max]; //ancestral locus for Harwich

int *offspring_flag = (int *) malloc((Npop*fecundity/2) * sizeof(int));

char **offspring_f_X_f = (char **) malloc((fecundity*Npop/4) * sizeof(char *)); // offspring pool for X loci in females from female

char **offspring_f_X_m = (char **) malloc((fecundity*Npop/4) * sizeof(char *)); // offspring pool for X loci in females from male

char **offspring_m_X = (char **) malloc((fecundity*Npop/4) * sizeof(char *)); // offspring pool for X loci in males

char **offspring_Y = (char **) malloc((fecundity*Npop/4) * sizeof(char *)); // offspring pool for Y loci

int *offspring_f_R2memory = (int *) malloc((fecundity*Npop/4) * sizeof(int)); // offspring memory in females of the R2 element

int *offspring_f_R1memory = (int *) malloc((fecundity*Npop/4) * sizeof(int)); // offspring memory in females of the R1 element

int *offspring_m_R2memory = (int *) malloc((fecundity*Npop/4) * sizeof(int)); // offspring memory in males of the R2 element

int *offspring_m_R1memory = (int *) malloc((fecundity*Npop/4) * sizeof(int)); // offspring memory in males of the R1 element

for(c=0;c<fecundity*Npop/4;c++){

offspring_f_X_f[c] = (char *) malloc(unitSize*size_max*sizeof(char));

offspring_f_X_m[c] = (char *) malloc(unitSize*size_max*sizeof(char));

offspring_m_X[c] = (char *) malloc(unitSize*size_max*sizeof(char));

offspring_Y[c] = (char *) malloc(unitSize*size_max*sizeof(char));

}

char **locus_f_X_f = (char **) malloc((Npop/2) * sizeof(char *)); //allocate locus pool(current generation) for X loci in females from female

char **locus_f_X_m = (char **) malloc((Npop/2) * sizeof(char *)); //allocate locus pool(current generation) for X loci in females from male

char **locus_m_X = (char **) malloc((Npop/2) * sizeof(char *)); //allocate locus pool(current generation) for X loci in males

char **locus_Y = (char **) malloc((Npop/2) * sizeof(char *)); //allocate locus pool(current generation) for Y loci

int *locus_f_R2memory = (int *) malloc((Npop/2) * sizeof(int)); // memory in females of the R2 element

int *locus_f_R1memory = (int *) malloc((Npop/2) * sizeof(int)); // memory in females of the R1 element

int *locus_m_R2memory = (int *) malloc((Npop/2) * sizeof(int)); // memory in males of the R2 element

int *locus_m_R1memory = (int *) malloc((Npop/2) * sizeof(int)); // memory in males of the R1 element

for(c=0;c<Npop/2;c++){

locus_f_X_f[c] = (char *) malloc(unitSize*size_max*sizeof(char));

locus_f_X_m[c] = (char *) malloc(unitSize*size_max*sizeof(char));

locus_m_X[c] = (char *) malloc(unitSize*size_max*sizeof(char));

locus_Y[c] = (char *) malloc(unitSize*size_max*sizeof(char));

}

char* child[fecundity]; //allocate child pool, size fecundity

for(c=0;c<fecundity;c++){

child[c]=malloc(unitSize*size_max*sizeof(char));

}

FILE *ftrend_X=fopen("trend_X.txt", "w"), *ftrend_Y=fopen("trend_Y.txt", "w");

FILE *fpopulation_X=fopen("population_X.txt", "w"), *fpopulation_Y=fopen("population_Y.txt", "w");

FILE *fpopulation_X_act=fopen("population_X_act.txt", "w"), *fpopulation_Y_act=fopen("population_Y_act.txt", "w");

FILE *fpopulation_X_inact=fopen("population_X_inact.txt", "w"), *fpopulation_Y_inact=fopen("population_Y_inact.txt", "w");

FILE *fR1trunc_X=fopen("R1trunc_X.txt","w"), *fR1trunc_Y=fopen("R1trunc_Y.txt","w");

FILE *fR2trunc_X=fopen("R2trunc_X.txt","w"), *fR2trunc_Y=fopen("R2trunc_Y.txt","w");

FILE *fR1trunc_X_act=fopen("R1trunc_X_act.txt","w"), *fR1trunc_Y_act=fopen("R1trunc_Y_act.txt","w");

FILE *fR2trunc_X_act=fopen("R2trunc_X_act.txt","w"), *fR2trunc_Y_act=fopen("R2trunc_Y_act.txt","w");

FILE *fR1trunc_X_inact=fopen("R1trunc_X_inact.txt","w"), *fR1trunc_Y_inact=fopen("R1trunc_Y_inact.txt","w");

FILE *fR2trunc_X_inact=fopen("R2trunc_X_inact.txt","w"), *fR2trunc_Y_inact=fopen("R2trunc_Y_inact.txt","w");

FILE *fR1sldwin_X=fopen("R1sldwin_X.txt","w"), *fR1sldwin_Y=fopen("R1sldwin_Y.txt","w");

FILE *fR2sldwin_X=fopen("R2sldwin_X.txt","w"), *fR2sldwin_Y=fopen("R2sldwin_Y.txt","w");

FILE *fUNsldwin_X=fopen("UNsldwin_X.txt","w"), *fUNsldwin_Y=fopen("UNsldwin_Y.txt","w");

FILE *fTRsldwin_X=fopen("TRsldwin_X.txt","w"), *fTRsldwin_Y=fopen("TRsldwin_Y.txt","w");

FILE *fR1sldwin_X_act=fopen("R1sldwin_X_act.txt","w"), *fR1sldwin_Y_act=fopen("R1sldwin_Y_act.txt","w");

FILE *fR2sldwin_X_act=fopen("R2sldwin_X_act.txt","w"), *fR2sldwin_Y_act=fopen("R2sldwin_Y_act.txt","w");

FILE *fUNsldwin_X_act=fopen("UNsldwin_X_act.txt","w"), *fUNsldwin_Y_act=fopen("UNsldwin_Y_act.txt","w");

FILE *fTRsldwin_X_act=fopen("TRsldwin_X_act.txt","w"), *fTRsldwin_Y_act=fopen("TRsldwin_Y_act.txt","w");

FILE *fR1sldwin_X_inact=fopen("R1sldwin_X_inact.txt","w"), *fR1sldwin_Y_inact=fopen("R1sldwin_Y_inact.txt","w");

FILE *fR2sldwin_X_inact=fopen("R2sldwin_X_inact.txt","w"), *fR2sldwin_Y_inact=fopen("R2sldwin_Y_inact.txt","w");

FILE *fUNsldwin_X_inact=fopen("UNsldwin_X_inact.txt","w"), *fUNsldwin_Y_inact=fopen("UNsldwin_Y_inact.txt","w");

FILE *fTRsldwin_X_inact=fopen("TRsldwin_X_inact.txt","w"), *fTRsldwin_Y_inact=fopen("TRsldwin_Y_inact.txt","w");

FILE *fR2frag_X=fopen("R2frag_X.txt","w"), *fR2frag_X_m=fopen("R2frag_X_m.txt","w"), *fR2frag_Y=fopen("R2frag_Y.txt","w");

FILE *fR2frag_X_act=fopen("R2frag_X_act.txt","w"), *fR2frag_X_act_m=fopen("R2frag_X_act_m.txt","w"), *fR2frag_Y_act=fopen("R2frag_Y_act.txt","w");

FILE *fR2frag_X_inact=fopen("R2frag_X_inact.txt","w"), *fR2frag_X_inact_m=fopen("R2frag_X_inact_m.txt","w"), *fR2frag_Y_inact=fopen("R2frag_Y_inact.txt","w");

FILE *fR1frag_X=fopen("R1frag_X.txt","w"), *fR1frag_X_m=fopen("R1frag_X_m.txt","w"), *fR1frag_Y=fopen("R1frag_Y.txt","w");

FILE *fR1frag_X_act=fopen("R1frag_X_act.txt","w"), *fR1frag_X_act_m=fopen("R1frag_X_act_m.txt","w"), *fR1frag_Y_act=fopen("R1frag_Y_act.txt","w");

FILE *fR1frag_X_inact=fopen("R1frag_X_inact.txt","w"), *fR1frag_X_inact_m=fopen("R1frag_X_inact_m.txt","w"), *fR1frag_Y_inact=fopen("R1frag_Y_inact.txt","w");

FILE *fendlocus_X=fopen("endlocus_X.txt","w"), *fendlocus_Y=fopen("endlocus_Y.txt","w");

FILE *fendlocus_X_act=fopen("endlocus_X_act.txt","w"), *fendlocus_Y_act=fopen("endlocus_Y_act.txt","w");

FILE *fendlocus_X_inact=fopen("endlocus_X_inact.txt","w"), *fendlocus_Y_inact=fopen("endlocus_Y_inact.txt","w");

FILE *fendtranscribe_f=fopen("endtranscribe_f.txt","w"), *fendtranscribe_m=fopen("endtranscribe_m.txt","w");

FILE *ftranscribe_f=fopen("transcribe_f.txt","w"), *ftranscribe_m=fopen("transcribe_m.txt","w");

FILE *fpopulation_transcribe_f=fopen("population_transcribe_f.txt", "w"), *fpopulation_transcribe_m=fopen("population_transcribe_m.txt", "w");

for(c=0;c<size_max+1;c++){

R1_trunc_copy_X[c] = 0;

R2_trunc_copy_X[c] = 0;

R1_trunc_copy_X_act[c] = 0;

R2_trunc_copy_X_act[c] = 0;

R1_trunc_copy_X_inact[c] = 0;

R2_trunc_copy_X_inact[c] = 0;

R1_trunc_copy_Y[c] = 0;

R2_trunc_copy_Y[c] = 0;

R1_trunc_copy_Y_act[c] = 0;

R2_trunc_copy_Y_act[c] = 0;

R1_trunc_copy_Y_inact[c] = 0;

R2_trunc_copy_Y_inact[c] = 0;

}

R1_trunc_max_X=0, R2_trunc_max_X=0;

R1_trunc_max_Y=0, R2_trunc_max_Y=0;

total_active_X=0, total_R2_active_X=0, total_R2_active_X_m=0, total_R1_active_X=0, total_R1_active_X_m=0;

total_inactive_X=0, total_R2_inactive_X=0, total_R2_inactive_X_m=0, total_R1_inactive_X=0, total_R1_inactive_X_m=0;

total_active_Y=0, total_R2_active_Y=0, total_R1_active_Y=0;

total_inactive_Y=0, total_R2_inactive_Y=0, total_R1_inactive_Y=0;

int c1,child1,child2;

int Un_X, R1_X, R2_X, db_X;

int Un_Y, R1_Y, R2_Y, db_Y;

int Un_X_act, R1_X_act, R2_X_act, db_X_act;

int Un_Y_act, R1_Y_act, R2_Y_act, db_Y_act;

int Un_X_inact, R1_X_inact, R2_X_inact, db_X_inact;

int Un_Y_inact, R1_Y_inact, R2_Y_inact, db_Y_inact;

int UnCurr0,R1Curr0,R2Curr0,dbCurr0;

int UnCurr1,R1Curr1,R2Curr1,dbCurr1;

int flR1Curr0,flR2Curr0;

int flR1Curr1,flR2Curr1;

int i,j;

int c_pop;

int Noffspring_f_X_f, Noffspring_f_X_m, Noffspring_m_X, Noffspring_Y;

int Nchild, mid;

int TranscribeUnCurr0, TranscribeUnCurr1;

int TranscribeR1Curr0, TranscribeR1Curr1;

int TranscribeR2Curr0, TranscribeR2Curr1;

int TranscribedbCurr0, TranscribedbCurr1;

int transcribedR1, transcribedR2;

int TranscribeUn_f, TranscribeR1_f, TranscribeR2_f, Transcribedb_f;

int TranscribeUn_m, TranscribeR1_m, TranscribeR2_m, Transcribedb_m;

int Nsick_f, Nsick_m;

int NR1active_m, NR2active_m;

int NR1active_f, NR2active_f;

int Nactive_X, Nactive_Y;

int Ninactive_X, Ninactive_Y;

int NnoR1_X, NnoR2_X;

int NnoR1_Y, NnoR2_Y;

int dead_population_flag;

int TRtotal;

int c_rep;

int c_gen;

for(c_rep=0;c_rep<Nreplicate;c_rep++){

t2 = time(NULL);

printf(" Run %d started at %s", c_rep+1, ctime(&t2));

printf(" ");

Noffspring_f_X_f=Npop/2;

Noffspring_f_X_m=Npop/2;

Noffspring_m_X=Npop/2;

Noffspring_Y=Npop/2;

if(single_ancester){

newLocus(ancester, initialLocusSize_X, initialR2level_X, initialR1level_X);

for(c_pop=0;c_pop<Noffspring_f_X_f;c_pop++){

strcpy(offspring_f_X_f[c_pop],ancester);

locus_f_R2memory[c_pop]=0;

locus_f_R1memory[c_pop]=0;

}

for(c_pop=0;c_pop<Noffspring_f_X_m;c_pop++){ strcpy(offspring_f_X_m[c_pop],ancester); }

for(c_pop=0;c_pop<Noffspring_m_X;c_pop++){

strcpy(offspring_m_X[c_pop],ancester);

locus_m_R2memory[c_pop]=0;

locus_m_R1memory[c_pop]=0;

}

if(!Y_present){ strcpy(ancester,"\0"); }

else{ newLocus(ancester,initialLocusSize_Y,initialR2level_Y,initialR1level_Y); }

for(c_pop=0;c_pop<Noffspring_Y;c_pop++){

strcpy(offspring_Y[c_pop], ancester);

}

}

else{

for(c_pop=0;c_pop<Noffspring_f_X_f;c_pop++){

newLocus(offspring_f_X_f[c_pop],initialLocusSize_X,initialR2level_X,initialR1level_X);

locus_f_R2memory[c_pop]=0;

locus_f_R1memory[c_pop]=0;

}

for(c_pop=0;c_pop<Noffspring_f_X_m;c_pop++){ newLocus(offspring_f_X_m[c_pop],initialLocusSize_X,initialR2level_X,initialR1level_X); }

for(c_pop=0;c_pop<Noffspring_m_X;c_pop++){

newLocus(offspring_m_X[c_pop],initialLocusSize_X,initialR2level_X,initialR1level_X);

locus_m_R2memory[c_pop]=0;

locus_m_R1memory[c_pop]=0;

}

if(!Y_present){

strcpy(ancester,"\0");

for(c_pop=0;c_pop<Noffspring_Y;c_pop++){

strcpy(offspring_Y[c_pop],ancester);

strcpy(locus_Y[c_pop], ancester);

}

}

else{ for(c_pop=0;c_pop<Noffspring_Y;c_pop++){ newLocus(offspring_Y[c_pop],initialLocusSize_Y,initialR2level_Y,initialR1level_Y); } }

}

dead_population_flag = 0;

canceledR2retro = 0, canceledR1retro = 0, canceledSCE = 0, canceledICE = 0;

for(c_gen=0;c_gen<Ngen;c_gen++){

if((c_gen+1)%(Ngen/4)==0){

printf("%d%%", ((c_gen+1)/(Ngen/4))*25);

if(canceledR2retro > 0){ printf(" [R2]x%d", canceledR2retro); }

if(canceledR1retro > 0){ printf(" [R1]x%d", canceledR1retro); }

if(canceledSCE > 0){ printf(" [SCE]x%d", canceledSCE); }

if(canceledICE > 0){ printf(" [ICE]x%d", canceledICE); }

printf("\n ");

canceledR2retro = 0, canceledR1retro = 0, canceledSCE = 0, canceledICE = 0;

}

else if((c_gen+1)%(Ngen/100)==0){ printf("* "); fflush(stdout); }

Un_X=0, R1_X=0, R2_X=0, db_X=0;

Un_Y=0, R1_Y=0, R2_Y=0, db_Y=0;

TranscribeUn_f=0, TranscribeR1_f=0, TranscribeR2_f=0, Transcribedb_f=0;

TranscribeUn_m=0, TranscribeR1_m=0, TranscribeR2_m=0, Transcribedb_m=0;

Nsick_f=0, Nsick_m=0;

NR1active_m=0, NR2active_m=0;

NR1active_f=0, NR2active_f=0;

NnoR1_X=0, NnoR2_X=0;

NnoR1_Y=0, NnoR2_Y=0;

if(!dead_population_flag && (Noffspring_f_X_f<Npop/2 || Noffspring_f_X_m<Npop/2 || Noffspring_m_X<Npop/2 || (Noffspring_Y<Npop/2 && Y_present))){

printf(" *** Population Died ***\n");

dead_population_flag=1;

}

if(!dead_population_flag){

//printf("%d--%d\n",c_rep,c_gen);

for(c_pop=0;c_pop<Npop*fecundity/2;c_pop++){

offspring_flag[c_pop]=0;

}

for (c_pop=0;c_pop<Npop/2;c_pop++){

do{ i=(int)(prob()*Noffspring_f_X_f); }while(i>=Noffspring_f_X_f || offspring_flag[i]);

strcpy(locus_f_X_f[c_pop], offspring_f_X_f[i]);

locus_f_R2memory[c_pop] = offspring_f_R2memory[i];

locus_f_R1memory[c_pop] = offspring_f_R1memory[i];

offspring_flag[i]=1;

}

for(c_pop=0;c_pop<Npop*fecundity/2;c_pop++){

offspring_flag[c_pop]=0;

}

for (c_pop=0;c_pop<Npop/2;c_pop++){

do{ i=(int)(prob()*Noffspring_f_X_m); }while(i>=Noffspring_f_X_m || offspring_flag[i]);

strcpy(locus_f_X_m[c_pop], offspring_f_X_m[i]);

offspring_flag[i]=1;

}

for(c_pop=0;c_pop<Npop*fecundity/2;c_pop++){

offspring_flag[c_pop]=0;

}

for (c_pop=0;c_pop<Npop/2;c_pop++){

do{ i=(int)(prob()*Noffspring_m_X); }while(i>=Noffspring_m_X || offspring_flag[i]);

strcpy(locus_m_X[c_pop], offspring_m_X[i]);

locus_m_R2memory[c_pop] = offspring_m_R2memory[i];

locus_m_R1memory[c_pop] = offspring_m_R1memory[i];

offspring_flag[i]=1;

}

if(Y_present){

for(c_pop=0;c_pop<Npop*fecundity/2;c_pop++){

offspring_flag[c_pop]=0;

}

for (c_pop=0;c_pop<Npop/2;c_pop++){

do{ i=(int)(prob()*Noffspring_Y); }while(i>=Noffspring_Y || offspring_flag[i]);

strcpy(locus_Y[c_pop], offspring_Y[i]);

offspring_flag[i]=1;

}

}

Noffspring_f_X_f = 0;

Noffspring_f_X_m = 0;

Noffspring_m_X = 0;

Noffspring_Y = 0;

for(c_pop=0;c_pop<Npop/2;c_pop++){ // Female flies

UnCurr0 = countUn(locus_f_X_f[c_pop], 0); //count the first chr.

R2Curr0 = countR2(locus_f_X_f[c_pop], 0, 0);

flR2Curr0 = countR2(locus_f_X_f[c_pop], 0, 2);

R1Curr0 = countR1(locus_f_X_f[c_pop], 0, 0);

flR1Curr0 = countR1(locus_f_X_f[c_pop], 0, 2);

dbCurr0 = countDouble(locus_f_X_f[c_pop], 0);

if(R2Curr0==0) {NnoR2_X++;}

if(R1Curr0==0) {NnoR1_X++;}

Un_X+=UnCurr0;

R2_X+=R2Curr0;

R1_X+=R1Curr0;

db_X+=dbCurr0;

UnCurr1 = countUn(locus_f_X_m[c_pop], 0); //count the second chr.

R2Curr1 = countR2(locus_f_X_m[c_pop], 0, 0);

flR2Curr1 = countR2(locus_f_X_m[c_pop], 0, 2);

R1Curr1 = countR1(locus_f_X_m[c_pop], 0, 0);

flR1Curr1 = countR1(locus_f_X_m[c_pop], 0, 2);

dbCurr1 = countDouble(locus_f_X_m[c_pop], 0);

if(R2Curr1==0) {NnoR2_X++;}

if(R1Curr1==0) {NnoR1_X++;}

Un_X+=UnCurr1;

R2_X+=R2Curr1;

R1_X+=R1Curr1;

db_X+=dbCurr1;

if(transcription_flag){

transcribeArea(locus_f_X_f[c_pop], transcription_size/2, locus_f_R2memory[c_pop], locus_f_R1memory[c_pop]);

transcribeArea(locus_f_X_m[c_pop], transcription_size/2, locus_f_R2memory[c_pop], locus_f_R1memory[c_pop]);

}

else{

transcribeAll(locus_f_X_f[c_pop]);

transcribeAll(locus_f_X_m[c_pop]);

}

if(nuc_dominance_flag){

nuclearDominance(locus_f_X_f[c_pop], locus_f_X_m[c_pop]);

}

TranscribeUnCurr0 = countUn(locus_f_X_f[c_pop], 1);

TranscribeR2Curr0 = countR2(locus_f_X_f[c_pop], 1, 2);

TranscribeR1Curr0 = countR1(locus_f_X_f[c_pop], 1, 2);

TranscribedbCurr0 = countDouble(locus_f_X_f[c_pop], 1);

TranscribeUnCurr1 = countUn(locus_f_X_m[c_pop], 1);

TranscribeR2Curr1 = countR2(locus_f_X_m[c_pop], 1, 2);

TranscribeR1Curr1 = countR1(locus_f_X_m[c_pop], 1, 2);

TranscribedbCurr1 = countDouble(locus_f_X_m[c_pop], 1);

TranscribeUn_f+=TranscribeUnCurr0;

TranscribeR2_f+=TranscribeR2Curr0;

TranscribeR1_f+=TranscribeR1Curr0;

Transcribedb_f+=TranscribedbCurr0;

TranscribeUn_f+=TranscribeUnCurr1;

TranscribeR2_f+=TranscribeR2Curr1;

TranscribeR1_f+=TranscribeR1Curr1;

Transcribedb_f+=TranscribedbCurr1;

if(TranscribeR2Curr0+TranscribeR2Curr1 > 0){ NR2active_f++; }

if(TranscribeR1Curr0+TranscribeR1Curr1 > 0){ NR1active_f++; }

Nchild = numOffspringF(TranscribeUnCurr0, TranscribeUnCurr1, TranscribeR2Curr0+TranscribeR2Curr1+TranscribeR1Curr0+TranscribeR1Curr1);

if(Nchild < fecundity){ Nsick_f++; }

mid = Nchild/2;

if(Nchild%2==1 && prob()<0.5){ mid++; }

for(child1=0;child1<mid;child1++){ strcpy(child[child1], locus_f_X_f[c_pop]); }

for(child2=mid;child2<Nchild;child2++){ strcpy(child[child2], locus_f_X_m[c_pop]); }

for(c1=0;c1<Nchild;c1++){

if(prob() < SCE_rate_f_X){ sischr(child[c1]); }

if(prob() < ((loop_rate * pow(strlen(child[c1])/unitSize, loop_func)) +

(R2_loop_rate * pow(TranscribeR2Curr0+TranscribeR2Curr1, R2_loop_func) * (TranscribeR2Curr0+TranscribeR2Curr1 !=0)) +

(R1_loop_rate * pow(TranscribeR1Curr0+TranscribeR1Curr1, R1_loop_func) * (TranscribeR1Curr0+TranscribeR1Curr1 !=0)))){ loopDeletion(child[c1]); }

if(prob() < 0.5){

if(prob() < ((trR2_retro_rate_f_X * pow(TranscribeR2Curr0+TranscribeR2Curr1, trR2_retro_func) * (TranscribeR2Curr0+TranscribeR2Curr1 !=0)) +

(R2_retro_rate_f_X * pow(flR2Curr0+flR2Curr1, R2_retro_func) * (flR2Curr0+flR2Curr1 !=0)) +

retro_without_R2) * ((double)(locus_f_R2memory[c_pop]==0) * retro_multiplier + (double)(locus_f_R2memory[c_pop]!=0))){ R2transposition(child[c1]); }

if(prob() < ((trR1_retro_rate_f_X * pow(TranscribeR1Curr0+TranscribeR1Curr1, trR1_retro_func) * (TranscribeR1Curr0+TranscribeR1Curr1 !=0)) +

(R1_retro_rate_f_X * pow(flR1Curr0+flR1Curr1, R1_retro_func) * (flR1Curr0+flR1Curr1 !=0)) +

retro_without_R1) * ((double)(locus_f_R1memory[c_pop]==0) * retro_multiplier + (double)(locus_f_R1memory[c_pop]!=0))){ R1transposition(child[c1]); }

}

else{

if(prob() < ((trR1_retro_rate_f_X * pow(TranscribeR1Curr0+TranscribeR1Curr1, trR1_retro_func) * (TranscribeR1Curr0+TranscribeR1Curr1 !=0)) +

(R1_retro_rate_f_X * pow(flR1Curr0+flR1Curr1, R1_retro_func) * (flR1Curr0+flR1Curr1 !=0)) +

retro_without_R1) * ((double)(locus_f_R1memory[c_pop]==0) * retro_multiplier + (double)(locus_f_R1memory[c_pop]!=0))){ R1transposition(child[c1]); }

if(prob() < ((trR2_retro_rate_f_X * pow(TranscribeR2Curr0+TranscribeR2Curr1, trR2_retro_func) * (TranscribeR2Curr0+TranscribeR2Curr1 !=0)) +

(R2_retro_rate_f_X * pow(flR2Curr0+flR2Curr1, R2_retro_func) * (flR2Curr0+flR2Curr1 !=0)) +

retro_without_R2) * ((double)(locus_f_R2memory[c_pop]==0) * retro_multiplier + (double)(locus_f_R2memory[c_pop]!=0))){ R2transposition(child[c1]); }

}

}

for(c1=0;c1+mid<Nchild;c1++){

if(prob() < ICE_rate_XX*2){ interce(child[c1],child[c1+mid]); }

}

for(c1=0;c1<Nchild;c1++){

if(Noffspring_f_X_f <= Noffspring_m_X){

strcpy(offspring_f_X_f[Noffspring_f_X_f], child[c1]);

if(locus_f_R2memory[c_pop] + memDelay <= 0){

offspring_f_R2memory[Noffspring_f_X_f] = memory;

}

else if(locus_f_R2memory[c_pop] > 0 && TranscribeR2Curr0+TranscribeR2Curr1 > 0){

offspring_f_R2memory[Noffspring_f_X_f] = memory;

}

else if(locus_f_R2memory[c_pop] == 0 && TranscribeR2Curr0+TranscribeR2Curr1 == 0){

offspring_f_R2memory[Noffspring_f_X_f] = locus_f_R2memory[c_pop];

}

else{

offspring_f_R2memory[Noffspring_f_X_f] = locus_f_R2memory[c_pop] - 1;

}

if(locus_f_R1memory[c_pop] + memDelay <= 0){

offspring_f_R1memory[Noffspring_f_X_f] = memory;

}

else if(locus_f_R1memory[c_pop] > 0 && TranscribeR1Curr0+TranscribeR1Curr1 > 0){

offspring_f_R1memory[Noffspring_f_X_f] = memory;

}

else if(locus_f_R1memory[c_pop] == 0 && TranscribeR1Curr0+TranscribeR1Curr1 == 0){

offspring_f_R1memory[Noffspring_f_X_f] = locus_f_R1memory[c_pop];

}

else{

offspring_f_R1memory[Noffspring_f_X_f] = locus_f_R1memory[c_pop] - 1;

}

Noffspring_f_X_f++;

}

else{

strcpy(offspring_m_X[Noffspring_m_X], child[c1]);

offspring_m_R2memory[Noffspring_m_X] = locus_m_R2memory[c_pop];

offspring_m_R1memory[Noffspring_m_X] = locus_m_R1memory[c_pop];

if(locus_m_R2memory[c_pop] + memDelay <= 0){

offspring_m_R2memory[Noffspring_m_X] = memory;

}

else if(locus_m_R2memory[c_pop] > 0 && TranscribeR2Curr0+TranscribeR2Curr1 > 0){

offspring_m_R2memory[Noffspring_m_X] = memory;

}

else if(locus_m_R2memory[c_pop] == 0 && TranscribeR2Curr0+TranscribeR2Curr1 == 0){

offspring_m_R2memory[Noffspring_m_X] = locus_m_R2memory[c_pop];

}

else{

offspring_m_R2memory[Noffspring_m_X] = locus_m_R2memory[c_pop] - 1;

}

if(locus_m_R1memory[c_pop] + memDelay <= 0){

offspring_m_R1memory[Noffspring_m_X] = memory;

}

else if(locus_m_R1memory[c_pop] > 0 && TranscribeR1Curr0+TranscribeR1Curr1 > 0){

offspring_m_R1memory[Noffspring_m_X] = memory;

}

else if(locus_m_R1memory[c_pop] == 0 && TranscribeR1Curr0+TranscribeR1Curr1 == 0){

offspring_m_R1memory[Noffspring_m_X] = locus_m_R1memory[c_pop];

}

else{

offspring_m_R1memory[Noffspring_m_X] = locus_m_R1memory[c_pop] - 1;

}

Noffspring_m_X++;

}

}

}

for(c_pop=0;c_pop<Npop/2;c_pop++){ // Male flies

UnCurr0 = countUn(locus_m_X[c_pop], 0); //count the first chr.

R2Curr0 = countR2(locus_m_X[c_pop], 0, 0);

flR2Curr0 = countR2(locus_m_X[c_pop], 0, 2);

R1Curr0 = countR1(locus_m_X[c_pop], 0, 0);

flR1Curr0 = countR1(locus_m_X[c_pop], 0, 2);

dbCurr0 = countDouble(locus_m_X[c_pop], 0);

if(R2Curr0==0) {NnoR2_X++;}

if(R1Curr0==0) {NnoR1_X++;}

Un_X+=UnCurr0;

R2_X+=R2Curr0;

R1_X+=R1Curr0;

db_X+=dbCurr0;

UnCurr1 = countUn(locus_Y[c_pop], 0); //count the second chr.

R2Curr1 = countR2(locus_Y[c_pop], 0, 0);

flR2Curr1 = countR2(locus_Y[c_pop], 0, 2);

R1Curr1 = countR1(locus_Y[c_pop], 0, 0);

flR1Curr1 = countR1(locus_Y[c_pop], 0, 2);

dbCurr1 = countDouble(locus_Y[c_pop], 0);

if(R2Curr1==0) {NnoR2_Y++;}

if(R1Curr1==0) {NnoR1_Y++;}

Un_Y+=UnCurr1;

R2_Y+=R2Curr1;

R1_Y+=R1Curr1;

db_Y+=dbCurr1;

if(transcription_flag){

if(Y_dominance==-1 || !Y_present){

transcribeArea(locus_m_X[c_pop], transcription_size, locus_m_R2memory[c_pop], locus_m_R1memory[c_pop]);

transcribeNone(locus_Y[c_pop]);

}

else if(Y_dominance==1){

transcribeNone(locus_m_X[c_pop]);

transcribeArea(locus_Y[c_pop], transcription_size, locus_m_R2memory[c_pop], locus_m_R1memory[c_pop]);

}

else{

transcribeArea(locus_m_X[c_pop], transcription_size/2, locus_m_R2memory[c_pop], locus_m_R1memory[c_pop]);

transcribeArea(locus_Y[c_pop], transcription_size/2, locus_m_R2memory[c_pop], locus_m_R1memory[c_pop]);

}

}

else{

if(Y_dominance==-1 || !Y_present){

transcribeAll(locus_m_X[c_pop]);

transcribeNone(locus_Y[c_pop]);

}

else if(Y_dominance==1){

transcribeNone(locus_m_X[c_pop]);

transcribeAll(locus_Y[c_pop]);

}

else{

transcribeAll(locus_m_X[c_pop]);

transcribeAll(locus_Y[c_pop]);

}

}

if(nuc_dominance_flag && Y_present && Y_dominance==0){

if(Y_dominance==0){ nuclearDominance(locus_m_X[c_pop], locus_Y[c_pop]); }

}

TranscribeUnCurr0 = countUn(locus_m_X[c_pop], 1);

TranscribeR2Curr0 = countR2(locus_m_X[c_pop], 1, 2);

TranscribeR1Curr0 = countR1(locus_m_X[c_pop], 1, 2);

TranscribedbCurr0 = countDouble(locus_m_X[c_pop], 1);

TranscribeUnCurr1 = countUn(locus_Y[c_pop], 1);

TranscribeR2Curr1 = countR2(locus_Y[c_pop], 1, 2);

TranscribeR1Curr1 = countR1(locus_Y[c_pop], 1, 2);

TranscribedbCurr1 = countDouble(locus_Y[c_pop], 1);

TranscribeUn_m+=TranscribeUnCurr0;

TranscribeR2_m+=TranscribeR2Curr0;

TranscribeR1_m+=TranscribeR1Curr0;

Transcribedb_m+=TranscribedbCurr0;

TranscribeUn_m+=TranscribeUnCurr1;

TranscribeR2_m+=TranscribeR2Curr1;

TranscribeR1_m+=TranscribeR1Curr1;

Transcribedb_m+=TranscribedbCurr1;

if(TranscribeR2Curr0+TranscribeR2Curr1 > 0){ NR2active_m++; }

if(TranscribeR1Curr0+TranscribeR1Curr1 > 0){ NR1active_m++; }

Nchild = numOffspringM(TranscribeUnCurr0, TranscribeUnCurr1, TranscribeR2Curr0+TranscribeR2Curr1+TranscribeR1Curr0+TranscribeR1Curr1);

if(Nchild < fecundity){ Nsick_m++; }

mid = Nchild/2;

if(Nchild%2==1 && prob()<0.5){ mid++; }

for(child1=0;child1<mid;child1++){ strcpy(child[child1], locus_m_X[c_pop]); }

for(c1=0;c1<mid;c1++){

if(prob() < SCE_rate_m_X){ sischr(child[c1]); }

if(prob() < ((loop_rate * pow(strlen(child[c1])/unitSize, loop_func)) +

(R2_loop_rate * pow(TranscribeR2Curr0+TranscribeR2Curr1, R2_loop_func) * (TranscribeR2Curr0+TranscribeR2Curr1 !=0)) +

(R1_loop_rate * pow(TranscribeR1Curr0+TranscribeR1Curr1, R1_loop_func) * (TranscribeR1Curr0+TranscribeR1Curr1 !=0)))){ loopDeletion(child[c1]); }

if(prob() < 0.5){

if(prob() < ((trR2_retro_rate_m_X * pow(TranscribeR2Curr0+TranscribeR2Curr1, trR2_retro_func) * (TranscribeR2Curr0+TranscribeR2Curr1 !=0)) +

(R2_retro_rate_m_X * pow(flR2Curr0+flR2Curr1, R2_retro_func) * (flR2Curr0+flR2Curr1 !=0)) +

retro_without_R2) * ((double)(locus_m_R2memory[c_pop]==0) * retro_multiplier + (double)(locus_m_R2memory[c_pop]!=0))){ R2transposition(child[c1]); }

if(prob() < ((trR1_retro_rate_m_X * pow(TranscribeR1Curr0+TranscribeR1Curr1, trR1_retro_func) * (TranscribeR1Curr0+TranscribeR1Curr1 !=0)) +

(R1_retro_rate_m_X * pow(flR1Curr0+flR1Curr1, R1_retro_func) * (flR1Curr0+flR1Curr1 !=0)) +

retro_without_R1) * ((double)(locus_m_R1memory[c_pop]==0) * retro_multiplier + (double)(locus_m_R1memory[c_pop]!=0))){ R1transposition(child[c1]); }

}

else{

if(prob() < ((trR1_retro_rate_m_X * pow(TranscribeR1Curr0+TranscribeR1Curr1, trR1_retro_func) * (TranscribeR1Curr0+TranscribeR1Curr1 !=0)) +

(R1_retro_rate_m_X * pow(flR1Curr0+flR1Curr1, R1_retro_func) * (flR1Curr0+flR1Curr1 !=0)) +

retro_without_R1) * ((double)(locus_m_R1memory[c_pop]==0) * retro_multiplier + (double)(locus_m_R1memory[c_pop]!=0))){ R1transposition(child[c1]); }

if(prob() < ((trR2_retro_rate_m_X * pow(TranscribeR2Curr0+TranscribeR2Curr1, trR2_retro_func) * (TranscribeR2Curr0+TranscribeR2Curr1 !=0)) +

(R2_retro_rate_m_X * pow(flR2Curr0+flR2Curr1, R2_retro_func) * (flR2Curr0+flR2Curr1 !=0)) +

retro_without_R2) * ((double)(locus_m_R2memory[c_pop]==0) * retro_multiplier + (double)(locus_m_R2memory[c_pop]!=0))){ R2transposition(child[c1]); }

}

}

if(Y_present){

for(child2=mid;child2<Nchild;child2++){ strcpy(child[child2], locus_Y[c_pop]); }

for(c1=mid;c1<Nchild;c1++){

if(prob() < SCE_rate_Y){ sischr(child[c1]); }

if(prob() < ((loop_rate * pow(strlen(child[c1])/unitSize, loop_func)) +

(R2_loop_rate * pow(TranscribeR2Curr0+TranscribeR2Curr1, R2_loop_func) * (TranscribeR2Curr0+TranscribeR2Curr1 !=0)) +

(R1_loop_rate * pow(TranscribeR1Curr0+TranscribeR1Curr1, R1_loop_func) * (TranscribeR1Curr0+TranscribeR1Curr1 !=0)))){ loopDeletion(child[c1]); }

if(prob() < 0.5){

if(prob() < ((trR2_retro_rate_m_X * pow(TranscribeR2Curr0+TranscribeR2Curr1, trR2_retro_func) * (TranscribeR2Curr0+TranscribeR2Curr1 !=0)) +

(R2_retro_rate_m_X * pow(flR2Curr0+flR2Curr1, R2_retro_func) * (flR2Curr0+flR2Curr1 !=0)) +

retro_without_R2) * ((double)(locus_m_R2memory[c_pop]==0) * retro_multiplier + (double)(locus_m_R2memory[c_pop]!=0))){ R2transposition(child[c1]); }

if(prob() < ((trR1_retro_rate_m_X * pow(TranscribeR1Curr0+TranscribeR1Curr1, trR1_retro_func) * (TranscribeR1Curr0+TranscribeR1Curr1 !=0)) +

(R1_retro_rate_m_X * pow(flR1Curr0+flR1Curr1, R1_retro_func) * (flR1Curr0+flR1Curr1 !=0)) +

retro_without_R1) * ((double)(locus_m_R1memory[c_pop]==0) * retro_multiplier + (double)(locus_m_R1memory[c_pop]!=0))){ R1transposition(child[c1]); }

}

else{

if(prob() < ((trR1_retro_rate_m_X * pow(TranscribeR1Curr0+TranscribeR1Curr1, trR1_retro_func) * (TranscribeR1Curr0+TranscribeR1Curr1 !=0)) +

(R1_retro_rate_m_X * pow(flR1Curr0+flR1Curr1, R1_retro_func) * (flR1Curr0+flR1Curr1 !=0)) +

retro_without_R1) * ((double)(locus_m_R1memory[c_pop]==0) * retro_multiplier + (double)(locus_m_R1memory[c_pop]!=0))){ R1transposition(child[c1]); }

if(prob() < ((trR2_retro_rate_m_X * pow(TranscribeR2Curr0+TranscribeR2Curr1, trR2_retro_func) * (TranscribeR2Curr0+TranscribeR2Curr1 !=0)) +

(R2_retro_rate_m_X * pow(flR2Curr0+flR2Curr1, R2_retro_func) * (flR2Curr0+flR2Curr1 !=0)) +

retro_without_R2) * ((double)(locus_m_R2memory[c_pop]==0) * retro_multiplier + (double)(locus_m_R2memory[c_pop]!=0))){ R2transposition(child[c1]); }

}

}

for(c1=0;c1<Nchild/2;c1++){

if(prob() < ICE_rate_XY*2){ interce(child[c1],child[c1+mid]); }

}

for(c1=0;c1<mid;c1++){

strcpy(offspring_f_X_m[Noffspring_f_X_m], child[c1]);

Noffspring_f_X_m++;

}

for(c1=mid;c1<Nchild;c1++){

strcpy(offspring_Y[Noffspring_Y], child[c1]);

Noffspring_Y++;

}

}

else{

for(c1=0;c1<mid;c1++){

strcpy(offspring_f_X_m[Noffspring_f_X_m], child[c1]);

Noffspring_f_X_m++;

}

}

}

}

fprintf(ftrend_X, "%.3f\t%.3f\t%.3f\t%.3f\t%.3f\t%.3f\t%.3f\n", (float)(Un_X+R2_X+R1_X-db_X)/(Npop*3/2), (float)Un_X/(Npop*3/2), (float)R2_X/(Npop*3/2), (float)R1_X/(Npop*3/2), (float)db_X/(Npop*3/2), (float)NnoR2_X/(Npop*3/2), (float)NnoR1_X/(Npop*3/2));

if(Y_present){

fprintf(ftrend_Y, "%.3f\t%.3f\t%.3f\t%.3f\t%.3f\t%.3f\t%.3f\n", (float)(Un_Y+R2_Y+R1_Y-db_Y)/(Npop/2), (float)Un_Y/(Npop/2), (float)R2_Y/(Npop/2), (float)R1_Y/(Npop/2), (float)db_Y/(Npop/2), (float)NnoR2_Y/(Npop/2), (float)NnoR1_Y/(Npop/2));

}

fprintf(ftranscribe_f, "%.3f\t%.3f\t%.3f\t%.3f\t%.3f\t%.3f\t%.3f\n", (float)TranscribeUn_f/(Npop/2), (float)TranscribeR2_f/(Npop/2), (float)TranscribeR1_f/(Npop/2), (float)Transcribedb_f/(Npop/2), (float)NR2active_f/(Npop/2), (float)NR1active_f/(Npop/2), (float)Nsick_f/(Npop/2));

fprintf(ftranscribe_m, "%.3f\t%.3f\t%.3f\t%.3f\t%.3f\t%.3f\t%.3f\n", (float)TranscribeUn_m/(Npop/2), (float)TranscribeR2_m/(Npop/2), (float)TranscribeR1_m/(Npop/2), (float)Transcribedb_m/(Npop/2), (float)NR2active_m/(Npop/2), (float)NR1active_m/(Npop/2), (float)Nsick_m/(Npop/2));

} // End of generation

// Report final generation

Un_X=0, R1_X=0, R2_X=0, db_X=0;

Un_Y=0, R1_Y=0, R2_Y=0, db_Y=0;

Un_X_act=0, R1_X_act=0, R2_X_act=0, db_X_act=0;

Un_Y_act=0, R1_Y_act=0, R2_Y_act=0, db_Y_act=0;

Un_X_inact=0, R1_X_inact=0, R2_X_inact=0, db_X_inact=0;

Un_Y_inact=0, R1_Y_inact=0, R2_Y_inact=0, db_Y_inact=0;

TranscribeUn_f=0, TranscribeR1_f=0, TranscribeR2_f=0, Transcribedb_f=0;

TranscribeUn_m=0, TranscribeR1_m=0, TranscribeR2_m=0, Transcribedb_m=0;

Nsick_f=0, Nsick_m=0;

NR1active_m=0, NR2active_m=0;

NR1active_f=0, NR2active_f=0;

Nactive_X=0, Nactive_Y=0;

Ninactive_X=0, Ninactive_Y=0;

NnoR1_X=0, NnoR2_X=0;

NnoR1_Y=0, NnoR2_Y=0;

for(c_pop=0;c_pop<20;c_pop++){

R1sldwin_X[c_pop] = 0;

R2sldwin_X[c_pop] = 0;

UNsldwin_X[c_pop] = 0;

TRsldwin_X[c_pop] = 0;

R1sldwin_X_act[c_pop] = 0;

R2sldwin_X_act[c_pop] = 0;

UNsldwin_X_act[c_pop] = 0;

TRsldwin_X_act[c_pop] = 0;

R1sldwin_X_inact[c_pop] = 0;

R2sldwin_X_inact[c_pop] = 0;

UNsldwin_X_inact[c_pop] = 0;

TRsldwin_X_inact[c_pop] = 0;

R1sldwin_Y[c_pop] = 0;

R2sldwin_Y[c_pop] = 0;

UNsldwin_Y[c_pop] = 0;

TRsldwin_Y[c_pop] = 0;

R1sldwin_Y_act[c_pop] = 0;

R2sldwin_Y_act[c_pop] = 0;

UNsldwin_Y_act[c_pop] = 0;

TRsldwin_Y_act[c_pop] = 0;

R1sldwin_Y_inact[c_pop] = 0;

R2sldwin_Y_inact[c_pop] = 0;

UNsldwin_Y_inact[c_pop] = 0;

TRsldwin_Y_inact[c_pop] = 0;

}

if(!dead_population_flag && (Noffspring_f_X_f<Npop/2 || Noffspring_f_X_m<Npop/2 || Noffspring_m_X<Npop/2 || (Noffspring_Y<Npop/2 && Y_present))){

printf(" *** Population Died ***\n");

dead_population_flag=1;

}

if(!dead_population_flag){

for(c_pop=0;c_pop<Npop*fecundity/2;c_pop++){

offspring_flag[c_pop]=0;

}

for (c_pop=0;c_pop<Npop/2;c_pop++){

do{ i=(int)(prob()*Noffspring_f_X_f); }while(i>=Noffspring_f_X_f || offspring_flag[i]);

strcpy(locus_f_X_f[c_pop], offspring_f_X_f[i]);

locus_f_R2memory[c_pop] = offspring_f_R2memory[i];

locus_f_R1memory[c_pop] = offspring_f_R1memory[i];

offspring_flag[i]=1;

}

for(c_pop=0;c_pop<Npop*fecundity/2;c_pop++){

offspring_flag[c_pop]=0;

}

for (c_pop=0;c_pop<Npop/2;c_pop++){

do{ i=(int)(prob()*Noffspring_f_X_m); }while(i>=Noffspring_f_X_m || offspring_flag[i]);

strcpy(locus_f_X_m[c_pop], offspring_f_X_m[i]);

offspring_flag[i]=1;

}

for(c_pop=0;c_pop<Npop*fecundity/2;c_pop++){

offspring_flag[c_pop]=0;

}

for (c_pop=0;c_pop<Npop/2;c_pop++){

do{ i=(int)(prob()*Noffspring_m_X); }while(i>=Noffspring_m_X || offspring_flag[i]);

strcpy(locus_m_X[c_pop], offspring_m_X[i]);

locus_m_R2memory[c_pop] = offspring_m_R2memory[i];

locus_m_R1memory[c_pop] = offspring_m_R1memory[i];

offspring_flag[i]=1;

}

if(Y_present){

for(c_pop=0;c_pop<Npop*fecundity/2;c_pop++){

offspring_flag[c_pop]=0;

}

for (c_pop=0;c_pop<Npop/2;c_pop++){

do{ i=(int)(prob()*Noffspring_Y); }while(i>=Noffspring_Y || offspring_flag[i]);

strcpy(locus_Y[c_pop], offspring_Y[i]);

offspring_flag[i]=1;

}

}

for(c_pop=0;c_pop<Npop/2;c_pop++){ // Female flies

if(transcription_flag){

transcribeArea(locus_f_X_f[c_pop], transcription_size/2, locus_f_R2memory[c_pop], locus_f_R1memory[c_pop]);

transcribeArea(locus_f_X_m[c_pop], transcription_size/2, locus_f_R2memory[c_pop], locus_f_R1memory[c_pop]);

}

else{

transcribeAll(locus_f_X_f[c_pop]);

transcribeAll(locus_f_X_m[c_pop]);

}

if(nuc_dominance_flag){

nuclearDominance(locus_f_X_f[c_pop], locus_f_X_m[c_pop]);

}

TranscribeUnCurr0 = countUn(locus_f_X_f[c_pop], 1);

TranscribeR2Curr0 = countR2(locus_f_X_f[c_pop], 1, 2);

TranscribeR1Curr0 = countR1(locus_f_X_f[c_pop], 1, 2);

TranscribedbCurr0 = countDouble(locus_f_X_f[c_pop], 1);

TranscribeUnCurr1 = countUn(locus_f_X_m[c_pop], 1);

TranscribeR2Curr1 = countR2(locus_f_X_m[c_pop], 1, 2);

TranscribeR1Curr1 = countR1(locus_f_X_m[c_pop], 1, 2);

TranscribedbCurr1 = countDouble(locus_f_X_m[c_pop], 1);

TranscribeUn_f+=TranscribeUnCurr0;

TranscribeR2_f+=TranscribeR2Curr0;

TranscribeR1_f+=TranscribeR1Curr0;

Transcribedb_f+=TranscribedbCurr0;

TranscribeUn_f+=TranscribeUnCurr1;

TranscribeR2_f+=TranscribeR2Curr1;

TranscribeR1_f+=TranscribeR1Curr1;

Transcribedb_f+=TranscribedbCurr1;

if(TranscribeR2Curr0+TranscribeR2Curr1 > 0){ NR2active_f++; }

if(TranscribeR1Curr0+TranscribeR1Curr1 > 0){ NR1active_f++; }

UnCurr0 = countUn(locus_f_X_f[c_pop], 0); //count the first chr.

R2Curr0 = countR2(locus_f_X_f[c_pop], 0, 0);

R1Curr0 = countR1(locus_f_X_f[c_pop], 0, 0);

dbCurr0 = countDouble(locus_f_X_f[c_pop], 0);

if(R2Curr0==0) {NnoR2_X++;}

if(R1Curr0==0) {NnoR1_X++;}

Un_X+=UnCurr0;

R2_X+=R2Curr0;

R1_X+=R1Curr0;

db_X+=dbCurr0;

fragment_size(locus_f_X_f[c_pop]);

fprintf(fpopulation_X, "%d\t%d\t%d\t%d\t%d\n", UnCurr0+R2Curr0+R1Curr0-dbCurr0, UnCurr0, R2Curr0, R1Curr0, dbCurr0);

fprintf(fR2frag_X, "%d\t%d\t%d\t%d\t%d\n", R2_frag_size[0], R2_frag_size[1], R2_frag_size[2], R2_frag_size[3], R2_frag_size[4]);

fprintf(fR1frag_X, "%d\t%d\t%d\t%d\t%d\n", R1_frag_size[0], R1_frag_size[1], R1_frag_size[2], R1_frag_size[3], R1_frag_size[4]);

if(TranscribeR2Curr0+TranscribeR2Curr1>0 || TranscribeR1Curr0+TranscribeR1Curr1>0){

fprintf(fpopulation_X_act, "%d\t%d\t%d\t%d\t%d\n", UnCurr0+R2Curr0+R1Curr0-dbCurr0, UnCurr0, R2Curr0, R1Curr0, dbCurr0);

total_active_X++;

}

else{

fprintf(fpopulation_X_inact, "%d\t%d\t%d\t%d\t%d\n", UnCurr0+R2Curr0+R1Curr0-dbCurr0, UnCurr0, R2Curr0, R1Curr0, dbCurr0);

total_inactive_X++;

}

if(TranscribeR2Curr0+TranscribeR2Curr1>0){

fprintf(fR2frag_X_act, "%d\t%d\t%d\t%d\t%d\n", R2_frag_size[0], R2_frag_size[1], R2_frag_size[2], R2_frag_size[3], R2_frag_size[4]);

total_R2_active_X++;

}

else{

fprintf(fR2frag_X_inact, "%d\t%d\t%d\t%d\t%d\n", R2_frag_size[0], R2_frag_size[1], R2_frag_size[2], R2_frag_size[3], R2_frag_size[4]);

total_R2_inactive_X++;

}

if(TranscribeR1Curr0+TranscribeR1Curr1>0){

fprintf(fR1frag_X_act, "%d\t%d\t%d\t%d\t%d\n", R1_frag_size[0], R1_frag_size[1], R1_frag_size[2], R1_frag_size[3], R1_frag_size[4]);

total_R1_active_X++;

}

else{

fprintf(fR1frag_X_inact, "%d\t%d\t%d\t%d\t%d\n", R1_frag_size[0], R1_frag_size[1], R1_frag_size[2], R1_frag_size[3], R1_frag_size[4]);

total_R1_inactive_X++;

}

sldwin(locus_f_X_f[c_pop], 'X', TranscribeR2Curr0+TranscribeR2Curr1, TranscribeR1Curr0+TranscribeR1Curr1);

trunc_abundance(locus_f_X_f[c_pop], 'X', TranscribeR2Curr0+TranscribeR2Curr1, TranscribeR1Curr0+TranscribeR1Curr1);

UnCurr1 = countUn(locus_f_X_m[c_pop], 0); //count the second chr.

R2Curr1 = countR2(locus_f_X_m[c_pop], 0, 0);

R1Curr1 = countR1(locus_f_X_m[c_pop], 0, 0);

dbCurr1 = countDouble(locus_f_X_m[c_pop], 0);

if(R2Curr1==0) {NnoR2_X++;}

if(R1Curr1==0) {NnoR1_X++;}

Un_X+=UnCurr1;

R2_X+=R2Curr1;

R1_X+=R1Curr1;

db_X+=dbCurr1;

fragment_size(locus_f_X_m[c_pop]);

fprintf(fpopulation_X, "%d\t%d\t%d\t%d\t%d\n", UnCurr1+R2Curr1+R1Curr1-dbCurr1, UnCurr1, R2Curr1, R1Curr1, dbCurr1);

fprintf(fR2frag_X, "%d\t%d\t%d\t%d\t%d\n", R2_frag_size[0], R2_frag_size[1], R2_frag_size[2], R2_frag_size[3], R2_frag_size[4]);

fprintf(fR1frag_X, "%d\t%d\t%d\t%d\t%d\n", R1_frag_size[0], R1_frag_size[1], R1_frag_size[2], R1_frag_size[3], R1_frag_size[4]);

if(TranscribeR2Curr0+TranscribeR2Curr1>0 || TranscribeR1Curr0+TranscribeR1Curr1>0){

fprintf(fpopulation_X_act, "%d\t%d\t%d\t%d\t%d\n", UnCurr1+R2Curr1+R1Curr1-dbCurr1, UnCurr1, R2Curr1, R1Curr1, dbCurr1);

total_active_X++;

}

else{

fprintf(fpopulation_X_inact, "%d\t%d\t%d\t%d\t%d\n", UnCurr1+R2Curr1+R1Curr1-dbCurr1, UnCurr1, R2Curr1, R1Curr1, dbCurr1);

total_inactive_X++;

}

if(TranscribeR2Curr0+TranscribeR2Curr1>0){

fprintf(fR2frag_X_act, "%d\t%d\t%d\t%d\t%d\n", R2_frag_size[0], R2_frag_size[1], R2_frag_size[2], R2_frag_size[3], R2_frag_size[4]);

total_R2_active_X++;

}

else{

fprintf(fR2frag_X_inact, "%d\t%d\t%d\t%d\t%d\n", R2_frag_size[0], R2_frag_size[1], R2_frag_size[2], R2_frag_size[3], R2_frag_size[4]);

total_R2_inactive_X++;

}

if(TranscribeR1Curr0+TranscribeR1Curr1>0){

fprintf(fR1frag_X_act, "%d\t%d\t%d\t%d\t%d\n", R1_frag_size[0], R1_frag_size[1], R1_frag_size[2], R1_frag_size[3], R1_frag_size[4]);

total_R1_active_X++;

}

else{

fprintf(fR1frag_X_inact, "%d\t%d\t%d\t%d\t%d\n", R1_frag_size[0], R1_frag_size[1], R1_frag_size[2], R1_frag_size[3], R1_frag_size[4]);

total_R1_inactive_X++;

}

sldwin(locus_f_X_m[c_pop], 'X', TranscribeR2Curr0+TranscribeR2Curr1, TranscribeR1Curr0+TranscribeR1Curr1);

trunc_abundance(locus_f_X_m[c_pop], 'X', TranscribeR2Curr0+TranscribeR2Curr1, TranscribeR1Curr0+TranscribeR1Curr1);

if(TranscribeR2Curr0+TranscribeR2Curr1 > 0 || TranscribeR1Curr0+TranscribeR1Curr1 > 0){

Un_X_act+=UnCurr0;

R2_X_act+=R2Curr0;

R1_X_act+=R1Curr0;

db_X_act+=dbCurr0;

Un_X_act+=UnCurr1;

R2_X_act+=R2Curr1;

R1_X_act+=R1Curr1;

db_X_act+=dbCurr1;

Nactive_X+=2;

}

else{

Un_X_inact+=UnCurr0;

R2_X_inact+=R2Curr0;

R1_X_inact+=R1Curr0;

db_X_inact+=dbCurr0;

Un_X_inact+=UnCurr1;

R2_X_inact+=R2Curr1;

R1_X_inact+=R1Curr1;

db_X_inact+=dbCurr1;

Ninactive_X+=2;

}

fprintf(fpopulation_transcribe_f, "%d\t%d\t%d\t%d\n", TranscribeUnCurr0+TranscribeUnCurr1, TranscribeR2Curr0+TranscribeR2Curr1, TranscribeR1Curr0+TranscribeR1Curr1, TranscribedbCurr0+TranscribedbCurr1);

Nchild = numOffspringF(TranscribeUnCurr0, TranscribeUnCurr1, TranscribeR2Curr0+TranscribeR2Curr1+TranscribeR1Curr0+TranscribeR1Curr1);

if(Nchild < fecundity){ Nsick_f++; }

}

for(c_pop=0;c_pop<Npop/2;c_pop++){ // Male flies

if(transcription_flag){

if(Y_dominance==-1 || !Y_present){

transcribeArea(locus_m_X[c_pop], transcription_size, locus_m_R2memory[c_pop], locus_m_R1memory[c_pop]);

transcribeNone(locus_Y[c_pop]);

}

else if(Y_dominance==1){

transcribeNone(locus_m_X[c_pop]);

transcribeArea(locus_Y[c_pop], transcription_size, locus_m_R2memory[c_pop], locus_m_R1memory[c_pop]);

}

else{

transcribeArea(locus_m_X[c_pop], transcription_size/2, locus_m_R2memory[c_pop], locus_m_R1memory[c_pop]);

transcribeArea(locus_Y[c_pop], transcription_size/2, locus_m_R2memory[c_pop], locus_m_R1memory[c_pop]);

}

}

else{

if(Y_dominance==-1 || !Y_present){

transcribeAll(locus_m_X[c_pop]);

transcribeNone(locus_Y[c_pop]);

}

else if(Y_dominance==1){

transcribeNone(locus_m_X[c_pop]);

transcribeAll(locus_Y[c_pop]);

}

else{

transcribeAll(locus_m_X[c_pop]);

transcribeAll(locus_Y[c_pop]);

}

}

if(nuc_dominance_flag && Y_present && Y_dominance==0){

if(Y_dominance==0){ nuclearDominance(locus_m_X[c_pop], locus_Y[c_pop]); }

}

TranscribeUnCurr0 = countUn(locus_m_X[c_pop], 1);

TranscribeR2Curr0 = countR2(locus_m_X[c_pop], 1, 2);

TranscribeR1Curr0 = countR1(locus_m_X[c_pop], 1, 2);

TranscribedbCurr0 = countDouble(locus_m_X[c_pop], 1);

TranscribeUnCurr1 = countUn(locus_Y[c_pop], 1);

TranscribeR2Curr1 = countR2(locus_Y[c_pop], 1, 2);

TranscribeR1Curr1 = countR1(locus_Y[c_pop], 1, 2);

TranscribedbCurr1 = countDouble(locus_Y[c_pop], 1);

TranscribeUn_m+=TranscribeUnCurr0;

TranscribeR2_m+=TranscribeR2Curr0;

TranscribeR1_m+=TranscribeR1Curr0;

Transcribedb_m+=TranscribedbCurr0;

TranscribeUn_m+=TranscribeUnCurr1;

TranscribeR2_m+=TranscribeR2Curr1;

TranscribeR1_m+=TranscribeR1Curr1;

Transcribedb_m+=TranscribedbCurr1;

if(TranscribeR2Curr0+TranscribeR2Curr1 > 0){ NR2active_m++; }

if(TranscribeR1Curr0+TranscribeR1Curr1 > 0){ NR1active_m++; }

UnCurr0 = countUn(locus_m_X[c_pop], 0); //count the first chr.

R2Curr0 = countR2(locus_m_X[c_pop], 0, 0);

R1Curr0 = countR1(locus_m_X[c_pop], 0, 0);

dbCurr0 = countDouble(locus_m_X[c_pop], 0);

if(R2Curr0==0) {NnoR2_X++;}

if(R1Curr0==0) {NnoR1_X++;}

Un_X+=UnCurr0;

R2_X+=R2Curr0;

R1_X+=R1Curr0;

db_X+=dbCurr0;

fragment_size(locus_m_X[c_pop]);

fprintf(fpopulation_X, "%d\t%d\t%d\t%d\t%d\n", UnCurr0+R2Curr0+R1Curr0-dbCurr0, UnCurr0, R2Curr0, R1Curr0, dbCurr0);

fprintf(fR2frag_X, "%d\t%d\t%d\t%d\t%d\n", R2_frag_size[0], R2_frag_size[1], R2_frag_size[2], R2_frag_size[3], R2_frag_size[4]);

fprintf(fR2frag_X_m, "%d\t%d\t%d\t%d\t%d\n", R2_frag_size[0], R2_frag_size[1], R2_frag_size[2], R2_frag_size[3], R2_frag_size[4]);

fprintf(fR1frag_X, "%d\t%d\t%d\t%d\t%d\n", R1_frag_size[0], R1_frag_size[1], R1_frag_size[2], R1_frag_size[3], R1_frag_size[4]);

fprintf(fR1frag_X_m, "%d\t%d\t%d\t%d\t%d\n", R1_frag_size[0], R1_frag_size[1], R1_frag_size[2], R1_frag_size[3], R1_frag_size[4]);

if(TranscribeR2Curr0+TranscribeR2Curr1>0 || TranscribeR1Curr0+TranscribeR1Curr1>0){

fprintf(fpopulation_X_act, "%d\t%d\t%d\t%d\t%d\n", UnCurr0+R2Curr0+R1Curr0-dbCurr0, UnCurr0, R2Curr0, R1Curr0, dbCurr0);

total_active_X++;

}

else{

fprintf(fpopulation_X_inact, "%d\t%d\t%d\t%d\t%d\n", UnCurr0+R2Curr0+R1Curr0-dbCurr0, UnCurr0, R2Curr0, R1Curr0, dbCurr0);

total_inactive_X++;

}

if(TranscribeR2Curr0+TranscribeR2Curr1>0){

fprintf(fR2frag_X_act, "%d\t%d\t%d\t%d\t%d\n", R2_frag_size[0], R2_frag_size[1], R2_frag_size[2], R2_frag_size[3], R2_frag_size[4]);

fprintf(fR2frag_X_act_m, "%d\t%d\t%d\t%d\t%d\n", R2_frag_size[0], R2_frag_size[1], R2_frag_size[2], R2_frag_size[3], R2_frag_size[4]);

total_R2_active_X++;

total_R2_active_X_m++;

}

else{

fprintf(fR2frag_X_inact, "%d\t%d\t%d\t%d\t%d\n", R2_frag_size[0], R2_frag_size[1], R2_frag_size[2], R2_frag_size[3], R2_frag_size[4]);

fprintf(fR2frag_X_inact_m, "%d\t%d\t%d\t%d\t%d\n", R2_frag_size[0], R2_frag_size[1], R2_frag_size[2], R2_frag_size[3], R2_frag_size[4]);

total_R2_inactive_X++;

total_R2_inactive_X_m++;

}

if(TranscribeR1Curr0+TranscribeR1Curr1>0){

fprintf(fR1frag_X_act, "%d\t%d\t%d\t%d\t%d\n", R1_frag_size[0], R1_frag_size[1], R1_frag_size[2], R1_frag_size[3], R1_frag_size[4]);

fprintf(fR1frag_X_act_m, "%d\t%d\t%d\t%d\t%d\n", R1_frag_size[0], R1_frag_size[1], R1_frag_size[2], R1_frag_size[3], R1_frag_size[4]);

total_R1_active_X++;

total_R1_active_X_m++;

}

else{

fprintf(fR1frag_X_inact, "%d\t%d\t%d\t%d\t%d\n", R1_frag_size[0], R1_frag_size[1], R1_frag_size[2], R1_frag_size[3], R1_frag_size[4]);

fprintf(fR1frag_X_inact_m, "%d\t%d\t%d\t%d\t%d\n", R1_frag_size[0], R1_frag_size[1], R1_frag_size[2], R1_frag_size[3], R1_frag_size[4]);

total_R1_inactive_X++;

total_R1_inactive_X_m++;

}

sldwin(locus_m_X[c_pop], 'X', TranscribeR2Curr0+TranscribeR2Curr1, TranscribeR1Curr0+TranscribeR1Curr1);

trunc_abundance(locus_m_X[c_pop], 'X', TranscribeR2Curr0+TranscribeR2Curr1, TranscribeR1Curr0+TranscribeR1Curr1);

UnCurr1 = countUn(locus_Y[c_pop], 0); //count the second chr.

R2Curr1 = countR2(locus_Y[c_pop], 0, 0);

R1Curr1 = countR1(locus_Y[c_pop], 0, 0);

dbCurr1 = countDouble(locus_Y[c_pop], 0);

if(R2Curr1==0) {NnoR2_Y++;}

if(R1Curr1==0) {NnoR1_Y++;}

Un_Y+=UnCurr1;

R2_Y+=R2Curr1;

R1_Y+=R1Curr1;

db_Y+=dbCurr1;

if(Y_present){

fragment_size(locus_Y[c_pop]);

fprintf(fpopulation_Y, "%d\t%d\t%d\t%d\t%d\n", UnCurr1+R2Curr1+R1Curr1-dbCurr1, UnCurr1, R2Curr1, R1Curr1, dbCurr1);

fprintf(fR2frag_Y, "%d\t%d\t%d\t%d\t%d\n", R2_frag_size[0], R2_frag_size[1], R2_frag_size[2], R2_frag_size[3], R2_frag_size[4]);

fprintf(fR1frag_Y, "%d\t%d\t%d\t%d\t%d\n", R1_frag_size[0], R1_frag_size[1], R1_frag_size[2], R1_frag_size[3], R1_frag_size[4]);

if(TranscribeR2Curr0+TranscribeR2Curr1>0 || TranscribeR1Curr0+TranscribeR1Curr1>0){

fprintf(fpopulation_Y_act, "%d\t%d\t%d\t%d\t%d\n", UnCurr1+R2Curr1+R1Curr1-dbCurr1, UnCurr1, R2Curr1, R1Curr1, dbCurr1);

total_active_Y++;

}

else{

fprintf(fpopulation_Y_inact, "%d\t%d\t%d\t%d\t%d\n", UnCurr1+R2Curr1+R1Curr1-dbCurr1, UnCurr1, R2Curr1, R1Curr1, dbCurr1);

total_inactive_Y++;

}

if(TranscribeR2Curr0+TranscribeR2Curr1>0){

fprintf(fR2frag_Y_act, "%d\t%d\t%d\t%d\t%d\n", R2_frag_size[0], R2_frag_size[1], R2_frag_size[2], R2_frag_size[3], R2_frag_size[4]);

total_R2_active_Y++;

}

else{

fprintf(fR2frag_Y_inact, "%d\t%d\t%d\t%d\t%d\n", R2_frag_size[0], R2_frag_size[1], R2_frag_size[2], R2_frag_size[3], R2_frag_size[4]);

total_R2_inactive_Y++;

}

if(TranscribeR1Curr0+TranscribeR1Curr1>0){

fprintf(fR1frag_Y_act, "%d\t%d\t%d\t%d\t%d\n", R1_frag_size[0], R1_frag_size[1], R1_frag_size[2], R1_frag_size[3], R1_frag_size[4]);

total_R1_active_Y++;

}

else{

fprintf(fR1frag_Y_inact, "%d\t%d\t%d\t%d\t%d\n", R1_frag_size[0], R1_frag_size[1], R1_frag_size[2], R1_frag_size[3], R1_frag_size[4]);

total_R1_inactive_Y++;

}

}

sldwin(locus_Y[c_pop], 'Y', TranscribeR2Curr0+TranscribeR2Curr1, TranscribeR1Curr0+TranscribeR1Curr1);

trunc_abundance(locus_Y[c_pop], 'Y', TranscribeR2Curr0+TranscribeR2Curr1, TranscribeR1Curr0+TranscribeR1Curr1);

if(TranscribeR2Curr0+TranscribeR2Curr1 > 0 || TranscribeR1Curr0+TranscribeR1Curr1 > 0){

Un_X_act+=UnCurr0;

R2_X_act+=R2Curr0;

R1_X_act+=R1Curr0;

db_X_act+=dbCurr0;

Un_Y_act+=UnCurr1;

R2_Y_act+=R2Curr1;

R1_Y_act+=R1Curr1;

db_Y_act+=dbCurr1;

Nactive_X++;

Nactive_Y++;

}

else{

Un_X_inact+=UnCurr0;

R2_X_inact+=R2Curr0;

R1_X_inact+=R1Curr0;

db_X_inact+=dbCurr0;

Un_X_inact+=UnCurr1;

R2_X_inact+=R2Curr1;

R1_X_inact+=R1Curr1;

db_X_inact+=dbCurr1;

Ninactive_X++;

Ninactive_Y++;

}

fprintf(fpopulation_transcribe_m, "%d\t%d\t%d\t%d\n", TranscribeUnCurr0+TranscribeUnCurr1, TranscribeR2Curr0+TranscribeR2Curr1, TranscribeR1Curr0+TranscribeR1Curr1, TranscribedbCurr0+TranscribedbCurr1);

Nchild = numOffspringM(TranscribeUnCurr0, TranscribeUnCurr1, TranscribeR2Curr0+TranscribeR2Curr1+TranscribeR1Curr0+TranscribeR1Curr1);

if(Nchild < fecundity){ Nsick_m++; }

}

}

fprintf(fendlocus_X, "%.3f\t%.3f\t%.3f\t%.3f\t%.3f\t%.3f\t%.3f\n", (float)(Un_X+R2_X+R1_X-db_X)/(Npop*3/2), (float)Un_X/(Npop*3/2), (float)R2_X/(Npop*3/2), (float)R1_X/(Npop*3/2), (float)db_X/(Npop*3/2), (float)NnoR2_X/(Npop*3/2), (float)NnoR1_X/(Npop*3/2));

fprintf(fendlocus_X_act, "%.3f\t%.3f\t%.3f\t%.3f\t%.3f\n", (float)(Un_X_act+R2_X_act+R1_X_act-db_X_act)/Nactive_X, (float)Un_X_act/Nactive_X, (float)R2_X_act/Nactive_X, (float)R1_X_act/Nactive_X, (float)db_X_act/Nactive_X);

fprintf(fendlocus_X_inact, "%.3f\t%.3f\t%.3f\t%.3f\t%.3f\n", (float)(Un_X_inact+R2_X_inact+R1_X_inact-db_X_inact)/Ninactive_X, (float)Un_X_inact/Ninactive_X, (float)R2_X_inact/Ninactive_X, (float)R1_X_inact/Ninactive_X, (float)db_X_inact/Ninactive_X);

if(Y_present){

fprintf(fendlocus_Y, "%.3f\t%.3f\t%.3f\t%.3f\t%.3f\t%.3f\t%.3f\n", (float)(Un_Y+R2_Y+R1_Y-db_Y)/(Npop/2), (float)Un_Y/(Npop/2), (float)R2_Y/(Npop/2), (float)R1_Y/(Npop/2), (float)db_Y/(Npop/2), (float)NnoR2_Y/(Npop/2), (float)NnoR1_Y/(Npop/2));

fprintf(fendlocus_Y_act, "%.3f\t%.3f\t%.3f\t%.3f\t%.3f\n", (float)(Un_Y_act+R2_Y_act+R1_Y_act-db_Y_act)/Nactive_Y, (float)Un_Y_act/Nactive_Y, (float)R2_Y_act/Nactive_Y, (float)R1_Y_act/Nactive_Y, (float)db_Y_act/Nactive_Y);

fprintf(fendlocus_Y_inact, "%.3f\t%.3f\t%.3f\t%.3f\t%.3f\n", (float)(Un_Y_inact+R2_Y_inact+R1_Y_inact-db_Y_inact)/Ninactive_Y, (float)Un_Y_inact/Ninactive_Y, (float)R2_Y_inact/Ninactive_Y, (float)R1_Y_inact/Ninactive_Y, (float)db_Y_inact/Ninactive_Y);

}

fprintf(fendtranscribe_f, "%.3f\t%.3f\t%.3f\t%.3f\t%.3f\t%.3f\t%.3f\n", (float)TranscribeUn_f/(Npop/2), (float)TranscribeR2_f/(Npop/2), (float)TranscribeR1_f/(Npop/2), (float)Transcribedb_f/(Npop/2), (float)NR2active_f/(Npop/2), (float)NR1active_f/(Npop/2), (float)Nsick_f/(Npop/2));

fprintf(fendtranscribe_m, "%.3f\t%.3f\t%.3f\t%.3f\t%.3f\t%.3f\t%.3f\n", (float)TranscribeUn_m/(Npop/2), (float)TranscribeR2_m/(Npop/2), (float)TranscribeR1_m/(Npop/2), (float)Transcribedb_m/(Npop/2), (float)NR2active_m/(Npop/2), (float)NR1active_m/(Npop/2), (float)Nsick_m/(Npop/2));

for(c_pop=0;c_pop<20;c_pop++){

fprintf(fUNsldwin_X, "%.3f\t", (float)UNsldwin_X[c_pop]/Un_X);

fprintf(fR2sldwin_X, "%.3f\t", (float)R2sldwin_X[c_pop]/R2_X);

fprintf(fR1sldwin_X, "%.3f\t", (float)R1sldwin_X[c_pop]/R1_X);

fprintf(fUNsldwin_X_act, "%.3f\t", (float)UNsldwin_X_act[c_pop]/Un_X);

fprintf(fR2sldwin_X_act, "%.3f\t", (float)R2sldwin_X_act[c_pop]/R2_X);

fprintf(fR1sldwin_X_act, "%.3f\t", (float)R1sldwin_X_act[c_pop]/R1_X);

fprintf(fUNsldwin_X_inact, "%.3f\t", (float)UNsldwin_X_inact[c_pop]/Un_X);

fprintf(fR2sldwin_X_inact, "%.3f\t", (float)R2sldwin_X_inact[c_pop]/R2_X);

fprintf(fR1sldwin_X_inact, "%.3f\t", (float)R1sldwin_X_inact[c_pop]/R1_X);

fprintf(fUNsldwin_Y, "%.3f\t", (float)UNsldwin_Y[c_pop]/Un_Y);

fprintf(fR2sldwin_Y, "%.3f\t", (float)R2sldwin_Y[c_pop]/R2_Y);

fprintf(fR1sldwin_Y, "%.3f\t", (float)R1sldwin_Y[c_pop]/R1_Y);

fprintf(fUNsldwin_Y_act, "%.3f\t", (float)UNsldwin_Y_act[c_pop]/Un_Y);

fprintf(fR2sldwin_Y_act, "%.3f\t", (float)R2sldwin_Y_act[c_pop]/R2_Y);

fprintf(fR1sldwin_Y_act, "%.3f\t", (float)R1sldwin_Y_act[c_pop]/R1_Y);

fprintf(fUNsldwin_Y_inact, "%.3f\t", (float)UNsldwin_Y_inact[c_pop]/Un_Y);

fprintf(fR2sldwin_Y_inact, "%.3f\t", (float)R2sldwin_Y_inact[c_pop]/R2_Y);

fprintf(fR1sldwin_Y_inact, "%.3f\t", (float)R1sldwin_Y_inact[c_pop]/R1_Y);

}

fprintf(fUNsldwin_X, "\n");

fprintf(fR2sldwin_X, "\n");

fprintf(fR1sldwin_X, "\n");

fprintf(fUNsldwin_X_act, "\n");

fprintf(fR2sldwin_X_act, "\n");

fprintf(fR1sldwin_X_act, "\n");

fprintf(fUNsldwin_X_inact, "\n");

fprintf(fR2sldwin_X_inact, "\n");

fprintf(fR1sldwin_X_inact, "\n");

fprintf(fUNsldwin_Y, "\n");

fprintf(fR2sldwin_Y, "\n");

fprintf(fR1sldwin_Y, "\n");

fprintf(fUNsldwin_Y_act, "\n");

fprintf(fR2sldwin_Y_act, "\n");

fprintf(fR1sldwin_Y_act, "\n");

fprintf(fUNsldwin_Y_inact, "\n");

fprintf(fR2sldwin_Y_inact, "\n");

fprintf(fR1sldwin_Y_inact, "\n");

TRtotal = 0;

for(c_pop=0;c_pop<20;c_pop++){ TRtotal+=TRsldwin_X[c_pop]; }

for(c_pop=0;c_pop<20;c_pop++){ fprintf(fTRsldwin_X, "%.3f\t", (float)TRsldwin_X[c_pop]/TRtotal); }

fprintf(fTRsldwin_X, "\n");

TRtotal = 0;

if(Y_present){

for(c_pop=0;c_pop<20;c_pop++){ TRtotal+=TRsldwin_Y[c_pop]; }

for(c_pop=0;c_pop<20;c_pop++){ fprintf(fTRsldwin_Y, "%.3f\t", (float)TRsldwin_Y[c_pop]/TRtotal); }

fprintf(fTRsldwin_Y, "\n");

}

t3 = time(NULL);

printf("Run %d finished at %s", c_rep+1, ctime(&t3));

time_diff = (int)(difftime(t3, t2));

t2 = time(NULL);

printf(" Run time: ");

if(time_diff/3600 > 0){

printf(" %dhr",time_diff/3600);

}

printf(" %dmin %dsec\n", (time_diff%3600)/60, time_diff%60);

} // End of replicate

int R2_trunc_total_X=0, R1_trunc_total_X=0, R2_trunc_total_Y=0, R1_trunc_total_Y=0;

for(c=1;c<=R1_trunc_max_X;c++){

R1_trunc_total_X+=R1_trunc_copy_X[c];

}

for(c=1;c<=R1_trunc_max_X;c++){

fprintf(fR1trunc_X, "%.6f\n", (float)R1_trunc_copy_X[c]/R1_trunc_total_X);

fprintf(fR1trunc_X_act, "%.6f\n", (float)R1_trunc_copy_X_act[c]/R1_trunc_total_X);

fprintf(fR1trunc_X_inact, "%.6f\n", (float)R1_trunc_copy_X_inact[c]/R1_trunc_total_X);

}

for(c=1;c<=R2_trunc_max_X;c++){

R2_trunc_total_X+=R2_trunc_copy_X[c];

}

for(c=1;c<=R2_trunc_max_X;c++){

fprintf(fR2trunc_X, "%.6f\n", (float)R2_trunc_copy_X[c]/R2_trunc_total_X);

fprintf(fR2trunc_X_act, "%.6f\n", (float)R2_trunc_copy_X_act[c]/R2_trunc_total_X);

fprintf(fR2trunc_X_inact, "%.6f\n", (float)R2_trunc_copy_X_inact[c]/R2_trunc_total_X);

}

for(c=1;c<=R1_trunc_max_Y;c++){

R1_trunc_total_Y+=R1_trunc_copy_Y[c];

}

for(c=1;c<=R1_trunc_max_Y;c++){

fprintf(fR1trunc_Y, "%.6f\n", (float)R1_trunc_copy_Y[c]/R1_trunc_total_Y);

fprintf(fR1trunc_Y_act, "%.6f\n", (float)R1_trunc_copy_Y_act[c]/R1_trunc_total_Y);

fprintf(fR1trunc_Y_inact, "%.6f\n", (float)R1_trunc_copy_Y_inact[c]/R1_trunc_total_Y);

}

for(c=1;c<=R2_trunc_max_Y;c++){

R2_trunc_total_Y+=R2_trunc_copy_Y[c];

}

for(c=1;c<=R2_trunc_max_Y;c++){

fprintf(fR2trunc_Y, "%.6f\n", (float)R2_trunc_copy_Y[c]/R2_trunc_total_Y);

fprintf(fR2trunc_Y_act, "%.6f\n", (float)R2_trunc_copy_Y_act[c]/R2_trunc_total_Y);

fprintf(fR2trunc_Y_inact, "%.6f\n", (float)R2_trunc_copy_Y_inact[c]/R2_trunc_total_Y);

}

for(c=0;c<fecundity*Npop/4;c++){

free(offspring_f_X_f[c]);

free(offspring_f_X_m[c]);

free(offspring_m_X[c]);

free(offspring_Y[c]);

}

for(c=0;c<Npop/2;c++){

free(locus_f_X_f[c]);

free(locus_f_X_m[c]);

free(locus_m_X[c]);

free(locus_Y[c]);

}

for(c=0;c<fecundity;c++){

free(child[c]);

}

free(offspring_flag);

free(offspring_f_R2memory);

free(offspring_f_R1memory);

free(offspring_m_R2memory);

free(offspring_m_R1memory);

free(locus_f_R2memory);

free(locus_f_R1memory);

free(locus_m_R2memory);

free(locus_m_R1memory);

fclose(ftrend_X); fclose(ftrend_Y);

fclose(fpopulation_X); fclose(fpopulation_Y);

fclose(fpopulation_X_act); fclose(fpopulation_Y_act);

fclose(fpopulation_X_inact); fclose(fpopulation_Y_inact);

fclose(fR1trunc_X); fclose(fR1trunc_Y);

fclose(fR2trunc_X); fclose(fR2trunc_Y);

fclose(fR1trunc_X_act); fclose(fR1trunc_Y_act);

fclose(fR2trunc_X_act); fclose(fR2trunc_Y_act);

fclose(fR1trunc_X_inact); fclose(fR1trunc_Y_inact);

fclose(fR2trunc_X_inact); fclose(fR2trunc_Y_inact);

fclose(fR1sldwin_X); fclose(fR1sldwin_Y);

fclose(fR2sldwin_X); fclose(fR2sldwin_Y);

fclose(fUNsldwin_X); fclose(fUNsldwin_Y);

fclose(fTRsldwin_X); fclose(fTRsldwin_Y);

fclose(fR1sldwin_X_act); fclose(fR1sldwin_Y_act);

fclose(fR2sldwin_X_act); fclose(fR2sldwin_Y_act);

fclose(fUNsldwin_X_act); fclose(fUNsldwin_Y_act);

fclose(fTRsldwin_X_act); fclose(fTRsldwin_Y_act);

fclose(fR1sldwin_X_inact); fclose(fR1sldwin_Y_inact);

fclose(fR2sldwin_X_inact); fclose(fR2sldwin_Y_inact);

fclose(fUNsldwin_X_inact); fclose(fUNsldwin_Y_inact);

fclose(fTRsldwin_X_inact); fclose(fTRsldwin_Y_inact);

fclose(fR2frag_X); fclose(fR2frag_X_m); fclose(fR2frag_Y);

fclose(fR2frag_X_act); fclose(fR2frag_X_act_m); fclose(fR2frag_Y_act);

fclose(fR2frag_X_inact); fclose(fR2frag_X_inact_m); fclose(fR2frag_Y_inact);

fclose(fR1frag_X); fclose(fR1frag_Y);

fclose(fR1frag_X_act); fclose(fR1frag_Y_act);

fclose(fR1frag_X_inact); fclose(fR1frag_Y_inact);

fclose(fendlocus_X); fclose(fendlocus_Y);

fclose(fendtranscribe_f); fclose(fendtranscribe_m);

fclose(ftranscribe_f); fclose(ftranscribe_m);

fclose(fpopulation_transcribe_f); fclose(fpopulation_transcribe_m);

// Sort data files

printf(" Sorting data"); fflush(stdout);

int ii,jj;

float* Trendls[Nreplicate];

float* TrendUn[Nreplicate];

float* TrendR2[Nreplicate];

float* TrendR1[Nreplicate];

float* Trenddb[Nreplicate];

float* TrendNoR2[Nreplicate];

float* TrendNoR1[Nreplicate];

float* TrendActiveR2[Nreplicate];

float* TrendActiveR1[Nreplicate];

float* Trendsick[Nreplicate];

float Sumls=0, SumUn=0, SumR2=0, SumR1=0, Sumdb=0, SumNoR2=0, SumNoR1=0, SumActiveR2=0, SumActiveR1=0, Sumsick=0;

for(ii=0;ii<Nreplicate;ii++){

Trendls[ii] = malloc(Ngen*sizeof(float));

TrendUn[ii] = malloc(Ngen*sizeof(float));

TrendR2[ii] = malloc(Ngen*sizeof(float));

TrendR1[ii] = malloc(Ngen*sizeof(float));

Trenddb[ii] = malloc(Ngen*sizeof(float));

TrendNoR2[ii] = malloc(Ngen*sizeof(float));

TrendNoR1[ii] = malloc(Ngen*sizeof(float));

TrendActiveR2[ii] = malloc(Ngen*sizeof(float));

TrendActiveR1[ii] = malloc(Ngen*sizeof(float));

Trendsick[ii] = malloc(Ngen*sizeof(float));

}

int locus_size_count=0, locus_un_count=0, locus_r1_count=0, locus_r2_count=0, locus_db_count=0;

int dist_ls[size_max+1];

int dist_un[size_max+1];

int dist_r1[size_max+1];

int dist_r2[size_max+1];

int dist_db[size_max+1];

int dist_r1_frac[1001];

int dist_r2_frac[1001];

int kk;

int dist_fragment[size_max+1][5];

// Sort trend_X.txt

FILE *fsort_trend_X=fopen("trend_X.txt","r");

FILE *ftrendsorted_X=fopen("trendsorted_X.txt","w");

for(ii=0;ii<Nreplicate;ii++){

for(jj=0;jj<Ngen;jj++){

fscanf(fsort_trend_X,"%f %f %f %f %f %f %f", &Trendls[ii][jj], &TrendUn[ii][jj], &TrendR2[ii][jj], &TrendR1[ii][jj], &Trenddb[ii][jj], &TrendNoR2[ii][jj], &TrendNoR1[ii][jj]);

}

}

for(jj=0;jj<Ngen;jj++){

Sumls=0, SumUn=0, SumR2=0, SumR1=0, Sumdb=0, SumNoR2=0, SumNoR1=0;

for(ii=0;ii<Nreplicate;ii++){

Sumls+=Trendls[ii][jj];

SumUn+=TrendUn[ii][jj];

SumR2+=TrendR2[ii][jj];

SumR1+=TrendR1[ii][jj];

Sumdb+=Trenddb[ii][jj];

SumNoR2+=TrendNoR2[ii][jj];

SumNoR1+=TrendNoR1[ii][jj];

}

fprintf(ftrendsorted_X,"%.3f\t%.3f\t%.3f\t%.3f\t%.3f\t%.3f\t%.3f\n", Sumls/Nreplicate, SumUn/Nreplicate, SumR2/Nreplicate, SumR1/Nreplicate, Sumdb/Nreplicate, SumNoR2/Nreplicate, SumNoR1/Nreplicate);

}

fclose(fsort_trend_X); fclose(ftrendsorted_X);

printf("."); fflush(stdout);

// Sort trend_Y.txt

FILE *fsort_trend_Y=fopen("trend_Y.txt","r");

FILE *ftrendsorted_Y=fopen("trendsorted_Y.txt","w");

if(Y_present){

for(ii=0;ii<Nreplicate;ii++){

for(jj=0;jj<Ngen;jj++){

fscanf(fsort_trend_X,"%f %f %f %f %f %f %f", &Trendls[ii][jj], &TrendUn[ii][jj], &TrendR2[ii][jj], &TrendR1[ii][jj], &Trenddb[ii][jj], &TrendNoR2[ii][jj], &TrendNoR1[ii][jj]);

}

}

for(jj=0;jj<Ngen;jj++){

Sumls=0, SumUn=0, SumR2=0, SumR1=0, Sumdb=0, SumNoR2=0, SumNoR1=0;

for(ii=0;ii<Nreplicate;ii++){

Sumls+=Trendls[ii][jj];

SumUn+=TrendUn[ii][jj];

SumR2+=TrendR2[ii][jj];

SumR1+=TrendR1[ii][jj];

Sumdb+=Trenddb[ii][jj];

SumNoR2+=TrendNoR2[ii][jj];

SumNoR1+=TrendNoR1[ii][jj];

}

fprintf(ftrendsorted_Y,"%.3f\t%.3f\t%.3f\t%.3f\t%.3f\t%.3f\t%.3f\n", Sumls/Nreplicate, SumUn/Nreplicate, SumR2/Nreplicate, SumR1/Nreplicate, Sumdb/Nreplicate, SumNoR2/Nreplicate, SumNoR1/Nreplicate);

}

}

fclose(fsort_trend_Y); fclose(ftrendsorted_Y);

printf("."); fflush(stdout);

// Sort transcribe_f.txt

FILE *fsort_transcribe_f=fopen("transcribe_f.txt","r");

FILE *ftranscribesorted_f=fopen("transcribesorted_f.txt","w");

for(ii=0;ii<Nreplicate;ii++){

for(jj=0;jj<Ngen;jj++){

fscanf(fsort_transcribe_f,"%f %f %f %f %f %f %f", &TrendUn[ii][jj], &TrendR2[ii][jj], &TrendR1[ii][jj], &Trenddb[ii][jj], &TrendActiveR2[ii][jj], &TrendActiveR1[ii][jj], &Trendsick[ii][jj]);

}

}

for(jj=0;jj<Ngen;jj++){

SumUn=0, SumR2=0, SumR1=0, Sumdb=0, SumActiveR2=0, SumActiveR1=0, Sumsick=0;

for(ii=0;ii<Nreplicate;ii++){

SumUn+=TrendUn[ii][jj];

SumR2+=TrendR2[ii][jj];

SumR1+=TrendR1[ii][jj];

Sumdb+=Trenddb[ii][jj];

SumActiveR2+=TrendActiveR2[ii][jj];

SumActiveR1+=TrendActiveR1[ii][jj];

Sumsick+=Trendsick[ii][jj];

}

fprintf(ftranscribesorted_f,"%.3f\t%.3f\t%.3f\t%.3f\t%.3f\t%.3f\t%.3f\n", SumUn/Nreplicate, SumR2/Nreplicate, SumR1/Nreplicate, Sumdb/Nreplicate, SumActiveR2/Nreplicate, SumActiveR1/Nreplicate, Sumsick/Nreplicate);

}

fclose(fsort_transcribe_f); fclose(ftranscribesorted_f);

printf("."); fflush(stdout);

// Sort transcribe_m.txt

FILE *fsort_transcribe_m=fopen("transcribe_m.txt","r");

FILE *ftranscribesorted_m=fopen("transcribesorted_m.txt","w");

for(ii=0;ii<Nreplicate;ii++){

for(jj=0;jj<Ngen;jj++){

fscanf(fsort_transcribe_m,"%f %f %f %f %f %f %f", &TrendUn[ii][jj], &TrendR2[ii][jj], &TrendR1[ii][jj], &Trenddb[ii][jj], &TrendActiveR2[ii][jj], &TrendActiveR1[ii][jj], &Trendsick[ii][jj]);

}

}

for(jj=0;jj<Ngen;jj++){

SumUn=0, SumR2=0, SumR1=0, Sumdb=0, SumActiveR2=0, SumActiveR1=0, Sumsick=0;

for(ii=0;ii<Nreplicate;ii++){

SumUn+=TrendUn[ii][jj];

SumR2+=TrendR2[ii][jj];

SumR1+=TrendR1[ii][jj];

Sumdb+=Trenddb[ii][jj];

SumActiveR2+=TrendActiveR2[ii][jj];

SumActiveR1+=TrendActiveR1[ii][jj];

Sumsick+=Trendsick[ii][jj];

}

fprintf(ftranscribesorted_m,"%.3f\t%.3f\t%.3f\t%.3f\t%.3f\t%.3f\t%.3f\n", SumUn/Nreplicate, SumR2/Nreplicate, SumR1/Nreplicate, Sumdb/Nreplicate, SumActiveR2/Nreplicate, SumActiveR1/Nreplicate, Sumsick/Nreplicate);

}

fclose(fsort_transcribe_m); fclose(ftranscribesorted_m);

printf("."); fflush(stdout);

// Distribution of population_X.txt

FILE *fpop_X=fopen("population_X.txt","r");

FILE *fdistribution_X=fopen("distribution_X.txt","w");

FILE *fr1frac_dist_X=fopen("R1frac_distribution_X.txt","w");

FILE *fr2frac_dist_X=fopen("R2frac_distribution_X.txt","w");

for(kk=0;kk<size_max+1;kk++){

dist_ls[kk]=0;

dist_un[kk]=0;

dist_r1[kk]=0;

dist_r2[kk]=0;

dist_db[kk]=0;

}

for(kk=0;kk<1001;kk++){

dist_r1_frac[kk]=0;

dist_r2_frac[kk]=0;

}

for(kk=0;kk<(Nreplicate*(Npop*3/2));kk++){

fscanf(fpop_X,"%d %d %d %d %d", &locus_size_count, &locus_un_count, &locus_r2_count, &locus_r1_count, &locus_db_count);

dist_ls[(locus_size_count)]++;

dist_un[locus_un_count]++;

dist_r1[locus_r1_count]++;

dist_r2[locus_r2_count]++;

dist_db[locus_db_count]++;

dist_r1_frac[1000*locus_r1_count/locus_size_count]++;

dist_r2_frac[1000*locus_r2_count/locus_size_count]++;

}

for(kk=0;kk<size_max+1;kk++){

fprintf(fdistribution_X,"%d\t%d\t%d\t%d\t%d\n",dist_ls[kk],dist_un[kk],dist_r2[kk],dist_r1[kk],dist_db[kk]);

}

for(kk=0;kk<1001;kk++){

fprintf(fr1frac_dist_X,"%d\n",dist_r1_frac[kk]);

fprintf(fr2frac_dist_X,"%d\n",dist_r2_frac[kk]);

}

fclose(fpop_X); fclose(fdistribution_X);

fclose(fr1frac_dist_X);

fclose(fr2frac_dist_X);

printf("."); fflush(stdout);

// Distribution of population_X_act.txt

FILE *fpop_X_act=fopen("population_X_act.txt","r");

FILE *fdistribution_X_act=fopen("distribution_X_act.txt","w");

FILE *fr1frac_dist_X_act=fopen("R1frac_distribution_X_act.txt","w");

FILE *fr2frac_dist_X_act=fopen("R2frac_distribution_X_act.txt","w");

for(kk=0;kk<size_max+1;kk++){

dist_ls[kk]=0;

dist_un[kk]=0;

dist_r1[kk]=0;

dist_r2[kk]=0;

dist_db[kk]=0;

}

for(kk=0;kk<1001;kk++){

dist_r1_frac[kk]=0;

dist_r2_frac[kk]=0;

}

for(kk=0;kk<total_active_X;kk++){

fscanf(fpop_X_act,"%d %d %d %d %d", &locus_size_count, &locus_un_count, &locus_r2_count, &locus_r1_count, &locus_db_count);

dist_ls[(locus_size_count)]++;

dist_un[locus_un_count]++;

dist_r1[locus_r1_count]++;

dist_r2[locus_r2_count]++;

dist_db[locus_db_count]++;

dist_r1_frac[1000*locus_r1_count/locus_size_count]++;

dist_r2_frac[1000*locus_r2_count/locus_size_count]++;

}

for(kk=0;kk<size_max+1;kk++){

fprintf(fdistribution_X_act,"%d\t%d\t%d\t%d\t%d\n",dist_ls[kk],dist_un[kk],dist_r2[kk],dist_r1[kk],dist_db[kk]);

}

for(kk=0;kk<1001;kk++){

fprintf(fr1frac_dist_X_act,"%d\n",dist_r1_frac[kk]);

fprintf(fr2frac_dist_X_act,"%d\n",dist_r2_frac[kk]);

}

fclose(fpop_X_act); fclose(fdistribution_X_act);

fclose(fr1frac_dist_X_act);

fclose(fr2frac_dist_X_act);

printf("."); fflush(stdout);

// Distribution of population_X_inact.txt

FILE *fpop_X_inact=fopen("population_X_inact.txt","r");

FILE *fdistribution_X_inact=fopen("distribution_X_inact.txt","w");

FILE *fr1frac_dist_X_inact=fopen("R1frac_distribution_X_inact.txt","w");

FILE *fr2frac_dist_X_inact=fopen("R2frac_distribution_X_inact.txt","w");

for(kk=0;kk<size_max+1;kk++){

dist_ls[kk]=0;

dist_un[kk]=0;

dist_r1[kk]=0;

dist_r2[kk]=0;

dist_db[kk]=0;

}

for(kk=0;kk<1001;kk++){

dist_r1_frac[kk]=0;

dist_r2_frac[kk]=0;

}

for(kk=0;kk<total_inactive_X;kk++){

fscanf(fpop_X_inact,"%d %d %d %d %d", &locus_size_count, &locus_un_count, &locus_r2_count, &locus_r1_count, &locus_db_count);

dist_ls[(locus_size_count)]++;

dist_un[locus_un_count]++;

dist_r1[locus_r1_count]++;

dist_r2[locus_r2_count]++;

dist_db[locus_db_count]++;

dist_r1_frac[1000*locus_r1_count/locus_size_count]++;

dist_r2_frac[1000*locus_r2_count/locus_size_count]++;

}

for(kk=0;kk<size_max+1;kk++){

fprintf(fdistribution_X_inact,"%d\t%d\t%d\t%d\t%d\n",dist_ls[kk],dist_un[kk],dist_r2[kk],dist_r1[kk],dist_db[kk]);

}

for(kk=0;kk<1001;kk++){

fprintf(fr1frac_dist_X_inact,"%d\n",dist_r1_frac[kk]);

fprintf(fr2frac_dist_X_inact,"%d\n",dist_r2_frac[kk]);

}

fclose(fpop_X_inact); fclose(fdistribution_X_inact);

fclose(fr1frac_dist_X_inact);

fclose(fr2frac_dist_X_inact);

printf("."); fflush(stdout);

// Distribution of population_Y.txt

FILE *fpop_Y=fopen("population_Y.txt","r");

FILE *fdistribution_Y=fopen("distribution_Y.txt","w");

FILE *fr1frac_dist_Y=fopen("R1frac_distribution_Y.txt","w");

FILE *fr2frac_dist_Y=fopen("R2frac_distribution_Y.txt","w");

if(Y_present){

for(kk=0;kk<size_max+1;kk++){

dist_ls[kk]=0;

dist_un[kk]=0;

dist_r1[kk]=0;

dist_r2[kk]=0;

dist_db[kk]=0;

}

for(kk=0;kk<1001;kk++){

dist_r1_frac[kk]=0;

dist_r2_frac[kk]=0;

}

for(kk=0;kk<(Nreplicate*(Npop*3/2));kk++){

fscanf(fpop_Y,"%d %d %d %d %d", &locus_size_count, &locus_un_count, &locus_r2_count, &locus_r1_count, &locus_db_count);

dist_ls[(locus_size_count)]++;

dist_un[locus_un_count]++;

dist_r1[locus_r1_count]++;

dist_r2[locus_r2_count]++;

dist_db[locus_db_count]++;

dist_r1_frac[1000*locus_r1_count/locus_size_count]++;

dist_r2_frac[1000*locus_r2_count/locus_size_count]++;

}

for(kk=0;kk<size_max+1;kk++){

fprintf(fdistribution_Y,"%d\t%d\t%d\t%d\t%d\n",dist_ls[kk],dist_un[kk],dist_r2[kk],dist_r1[kk],dist_db[kk]);

}

for(kk=0;kk<1001;kk++){

fprintf(fr1frac_dist_Y,"%d\n",dist_r1_frac[kk]);

fprintf(fr2frac_dist_Y,"%d\n",dist_r2_frac[kk]);

}

}

fclose(fpop_Y); fclose(fdistribution_Y);

fclose(fr1frac_dist_Y);

fclose(fr2frac_dist_Y);

printf("."); fflush(stdout);

// Distribution of population_Y_act.txt

FILE *fpop_Y_act=fopen("population_Y_act.txt","r");

FILE *fdistribution_Y_act=fopen("distribution_Y_act.txt","w");

FILE *fr1frac_dist_Y_act=fopen("R1frac_distribution_Y_act.txt","w");

FILE *fr2frac_dist_Y_act=fopen("R2frac_distribution_Y_act.txt","w");

if(Y_present){

for(kk=0;kk<size_max+1;kk++){

dist_ls[kk]=0;

dist_un[kk]=0;

dist_r1[kk]=0;

dist_r2[kk]=0;

dist_db[kk]=0;

}

for(kk=0;kk<1001;kk++){

dist_r1_frac[kk]=0;

dist_r2_frac[kk]=0;

}

for(kk=0;kk<total_active_Y;kk++){

fscanf(fpop_Y_act,"%d %d %d %d %d", &locus_size_count, &locus_un_count, &locus_r2_count, &locus_r1_count, &locus_db_count);

dist_ls[(locus_size_count)]++;

dist_un[locus_un_count]++;

dist_r1[locus_r1_count]++;

dist_r2[locus_r2_count]++;

dist_db[locus_db_count]++;

dist_r1_frac[1000*locus_r1_count/locus_size_count]++;

dist_r2_frac[1000*locus_r2_count/locus_size_count]++;

}

for(kk=0;kk<size_max+1;kk++){

fprintf(fdistribution_Y_act,"%d\t%d\t%d\t%d\t%d\n",dist_ls[kk],dist_un[kk],dist_r2[kk],dist_r1[kk],dist_db[kk]);

}

for(kk=0;kk<1001;kk++){

fprintf(fr1frac_dist_Y_act,"%d\n",dist_r1_frac[kk]);

fprintf(fr2frac_dist_Y_act,"%d\n",dist_r2_frac[kk]);

}

}

fclose(fpop_Y_act); fclose(fdistribution_Y_act);

fclose(fr1frac_dist_Y_act);

fclose(fr2frac_dist_Y_act);

printf("."); fflush(stdout);

// Distribution of population_Y_inact.txt

FILE *fpop_Y_inact=fopen("population_Y_inact.txt","r");

FILE *fdistribution_Y_inact=fopen("distribution_Y_inact.txt","w");

FILE *fr1frac_dist_Y_inact=fopen("R1frac_distribution_Y_inact.txt","w");

FILE *fr2frac_dist_Y_inact=fopen("R2frac_distribution_Y_inact.txt","w");

if(Y_present){

for(kk=0;kk<size_max+1;kk++){

dist_ls[kk]=0;

dist_un[kk]=0;

dist_r1[kk]=0;

dist_r2[kk]=0;

dist_db[kk]=0;

}

for(kk=0;kk<1001;kk++){

dist_r1_frac[kk]=0;

dist_r2_frac[kk]=0;

}

for(kk=0;kk<total_inactive_Y;kk++){

fscanf(fpop_Y_inact,"%d %d %d %d %d", &locus_size_count, &locus_un_count, &locus_r2_count, &locus_r1_count, &locus_db_count);

dist_ls[(locus_size_count)]++;

dist_un[locus_un_count]++;

dist_r1[locus_r1_count]++;

dist_r2[locus_r2_count]++;

dist_db[locus_db_count]++;

dist_r1_frac[1000*locus_r1_count/locus_size_count]++;

dist_r2_frac[1000*locus_r2_count/locus_size_count]++;

}

for(kk=0;kk<size_max+1;kk++){

fprintf(fdistribution_Y_inact,"%d\t%d\t%d\t%d\t%d\n",dist_ls[kk],dist_un[kk],dist_r2[kk],dist_r1[kk],dist_db[kk]);

}

for(kk=0;kk<1001;kk++){

fprintf(fr1frac_dist_Y_inact,"%d\n",dist_r1_frac[kk]);

fprintf(fr2frac_dist_Y_inact,"%d\n",dist_r2_frac[kk]);

}

}

fclose(fpop_Y_inact); fclose(fdistribution_Y_inact);

fclose(fr1frac_dist_Y_inact);

fclose(fr2frac_dist_Y_inact);

printf("."); fflush(stdout);

//Distribution of population_transcribe_f.txt

FILE *fpop_tr_f=fopen("population_transcribe_f.txt","r");

FILE *fdistribution_tr_f=fopen("distribution_transcribe_f.txt","w");

for(kk=0;kk<transcription_size+1;kk++){

dist_un[kk]=0;

dist_r1[kk]=0;

dist_r2[kk]=0;

dist_db[kk]=0;

}

for(kk=0;kk<(Nreplicate*(Npop/2));kk++){

fscanf(fpop_tr_f,"%d %d %d %d", &locus_un_count, &locus_r2_count, &locus_r1_count, &locus_db_count);

dist_un[locus_un_count]++;

dist_r1[locus_r1_count]++;

dist_r2[locus_r2_count]++;

dist_db[locus_db_count]++;

}

for(kk=0;kk<transcription_size+1;kk++){

fprintf(fdistribution_tr_f,"%d\t%d\t%d\t%d\n",dist_un[kk],dist_r2[kk],dist_r1[kk],dist_db[kk]);

}

fclose(fpop_tr_f); fclose(fdistribution_tr_f);

printf("."); fflush(stdout);

//Distribution of population_transcribe_m.txt

FILE *fpop_tr_m=fopen("population_transcribe_m.txt","r");

FILE *fdistribution_tr_m=fopen("distribution_transcribe_m.txt","w");

for(kk=0;kk<transcription_size+1;kk++){

dist_un[kk]=0;

dist_r1[kk]=0;

dist_r2[kk]=0;

dist_db[kk]=0;

}

for(kk=0;kk<(Nreplicate*(Npop/2));kk++){

fscanf(fpop_tr_m,"%d %d %d %d", &locus_un_count, &locus_r2_count, &locus_r1_count, &locus_db_count);

dist_un[locus_un_count]++;

dist_r1[locus_r1_count]++;

dist_r2[locus_r2_count]++;

dist_db[locus_db_count]++;

}

for(kk=0;kk<transcription_size+1;kk++){

fprintf(fdistribution_tr_m,"%d\t%d\t%d\t%d\n",dist_un[kk],dist_r2[kk],dist_r1[kk],dist_db[kk]);

}

fclose(fpop_tr_m); fclose(fdistribution_tr_m);

printf("."); fflush(stdout);

for(ii=0;ii<Nreplicate;ii++){

free(Trendls[ii]);

free(TrendUn[ii]);

free(TrendR2[ii]);

free(TrendR1[ii]);

free(Trenddb[ii]);

free(TrendNoR2[ii]);

free(TrendNoR1[ii]);

free(TrendActiveR2[ii]);

free(TrendActiveR1[ii]);

free(Trendsick[ii]);

}

//Distribution of R2frag_X.txt

FILE *fR2_frag_X=fopen("R2frag_X.txt","r");

FILE *fdist_R2frag_X=fopen("distribution_R2frag_X.txt","w");

for(kk=0;kk<size_max+1;kk++){

dist_fragment[kk][0] = 0;

dist_fragment[kk][1] = 0;

dist_fragment[kk][2] = 0;

dist_fragment[kk][3] = 0;

dist_fragment[kk][4] = 0;

}

for(kk=0;kk<(Nreplicate*(Npop*3/2));kk++){

fscanf(fR2_frag_X,"%d %d %d %d %d", &R2_frag_size[0], &R2_frag_size[1], &R2_frag_size[2], &R2_frag_size[3], &R2_frag_size[4]);

dist_fragment[R2_frag_size[0]][0]++;

dist_fragment[R2_frag_size[1]][1]++;

dist_fragment[R2_frag_size[2]][2]++;

dist_fragment[R2_frag_size[3]][3]++;

dist_fragment[R2_frag_size[4]][4]++;

}

for(kk=0;kk<size_max+1;kk++){

fprintf(fdist_R2frag_X,"%d\t%d\t%d\t%d\t%d\n", dist_fragment[kk][0], dist_fragment[kk][1], dist_fragment[kk][2], dist_fragment[kk][3], dist_fragment[kk][4]);

}

fclose(fR2_frag_X); fclose(fdist_R2frag_X);

printf("."); fflush(stdout);

//Distribution of R2frag_X_m.txt

FILE *fR2_frag_X_m=fopen("R2frag_X_m.txt","r");

FILE *fdist_R2frag_X_m=fopen("distribution_R2frag_X_m.txt","w");

for(kk=0;kk<size_max+1;kk++){

dist_fragment[kk][0] = 0;

dist_fragment[kk][1] = 0;

dist_fragment[kk][2] = 0;

dist_fragment[kk][3] = 0;

dist_fragment[kk][4] = 0;

}

for(kk=0;kk<(Nreplicate*(Npop/2));kk++){

fscanf(fR2_frag_X_m,"%d %d %d %d %d", &R2_frag_size[0], &R2_frag_size[1], &R2_frag_size[2], &R2_frag_size[3], &R2_frag_size[4]);

dist_fragment[R2_frag_size[0]][0]++;

dist_fragment[R2_frag_size[1]][1]++;

dist_fragment[R2_frag_size[2]][2]++;

dist_fragment[R2_frag_size[3]][3]++;

dist_fragment[R2_frag_size[4]][4]++;

}

for(kk=0;kk<size_max+1;kk++){

fprintf(fdist_R2frag_X_m,"%d\t%d\t%d\t%d\t%d\n", dist_fragment[kk][0], dist_fragment[kk][1], dist_fragment[kk][2], dist_fragment[kk][3], dist_fragment[kk][4]);

}

fclose(fR2_frag_X_m); fclose(fdist_R2frag_X_m);

printf("."); fflush(stdout);

//Distribution of R2frag_X_act.txt

FILE *fR2_frag_X_act=fopen("R2frag_X_act.txt","r");

FILE *fdist_R2frag_X_act=fopen("distribution_R2frag_X_act.txt","w");

for(kk=0;kk<size_max+1;kk++){

dist_fragment[kk][0] = 0;

dist_fragment[kk][1] = 0;

dist_fragment[kk][2] = 0;

dist_fragment[kk][3] = 0;

dist_fragment[kk][4] = 0;

}

for(kk=0;kk<total_R2_active_X;kk++){

fscanf(fR2_frag_X_act,"%d %d %d %d %d", &R2_frag_size[0], &R2_frag_size[1], &R2_frag_size[2], &R2_frag_size[3], &R2_frag_size[4]);

dist_fragment[R2_frag_size[0]][0]++;

dist_fragment[R2_frag_size[1]][1]++;

dist_fragment[R2_frag_size[2]][2]++;

dist_fragment[R2_frag_size[3]][3]++;

dist_fragment[R2_frag_size[4]][4]++;

}

for(kk=0;kk<size_max+1;kk++){

fprintf(fdist_R2frag_X_act,"%d\t%d\t%d\t%d\t%d\n", dist_fragment[kk][0], dist_fragment[kk][1], dist_fragment[kk][2], dist_fragment[kk][3], dist_fragment[kk][4]);

}

fclose(fR2_frag_X_act); fclose(fdist_R2frag_X_act);

printf("."); fflush(stdout);

//Distribution of R2frag_X_act_m.txt

FILE *fR2_frag_X_act_m=fopen("R2frag_X_act_m.txt","r");

FILE *fdist_R2frag_X_act_m=fopen("distribution_R2frag_X_act_m.txt","w");

for(kk=0;kk<size_max+1;kk++){

dist_fragment[kk][0] = 0;

dist_fragment[kk][1] = 0;

dist_fragment[kk][2] = 0;

dist_fragment[kk][3] = 0;

dist_fragment[kk][4] = 0;

}

for(kk=0;kk<total_R2_active_X_m;kk++){

fscanf(fR2_frag_X_act_m,"%d %d %d %d %d", &R2_frag_size[0], &R2_frag_size[1], &R2_frag_size[2], &R2_frag_size[3], &R2_frag_size[4]);

dist_fragment[R2_frag_size[0]][0]++;

dist_fragment[R2_frag_size[1]][1]++;

dist_fragment[R2_frag_size[2]][2]++;

dist_fragment[R2_frag_size[3]][3]++;

dist_fragment[R2_frag_size[4]][4]++;

}

for(kk=0;kk<size_max+1;kk++){

fprintf(fdist_R2frag_X_act_m,"%d\t%d\t%d\t%d\t%d\n", dist_fragment[kk][0], dist_fragment[kk][1], dist_fragment[kk][2], dist_fragment[kk][3], dist_fragment[kk][4]);

}

fclose(fR2_frag_X_act_m); fclose(fdist_R2frag_X_act_m);

printf("."); fflush(stdout);

//Distribution of R2frag_X_inact.txt

FILE *fR2_frag_X_inact=fopen("R2frag_X_inact.txt","r");

FILE *fdist_R2frag_X_inact=fopen("distribution_R2frag_X_inact.txt","w");

for(kk=0;kk<size_max+1;kk++){

dist_fragment[kk][0] = 0;

dist_fragment[kk][1] = 0;

dist_fragment[kk][2] = 0;

dist_fragment[kk][3] = 0;

dist_fragment[kk][4] = 0;

}

for(kk=0;kk<total_R2_inactive_X;kk++){

fscanf(fR2_frag_X_inact,"%d %d %d %d %d", &R2_frag_size[0], &R2_frag_size[1], &R2_frag_size[2], &R2_frag_size[3], &R2_frag_size[4]);

dist_fragment[R2_frag_size[0]][0]++;

dist_fragment[R2_frag_size[1]][1]++;

dist_fragment[R2_frag_size[2]][2]++;

dist_fragment[R2_frag_size[3]][3]++;

dist_fragment[R2_frag_size[4]][4]++;

}

for(kk=0;kk<size_max+1;kk++){

fprintf(fdist_R2frag_X_inact,"%d\t%d\t%d\t%d\t%d\n", dist_fragment[kk][0], dist_fragment[kk][1], dist_fragment[kk][2], dist_fragment[kk][3], dist_fragment[kk][4]);

}

fclose(fR2_frag_X_inact); fclose(fdist_R2frag_X_inact);

printf("."); fflush(stdout);

//Distribution of R2frag_X_inact_m.txt

FILE *fR2_frag_X_inact_m=fopen("R2frag_X_inact_m.txt","r");

FILE *fdist_R2frag_X_inact_m=fopen("distribution_R2frag_X_inact_m.txt","w");

for(kk=0;kk<size_max+1;kk++){

dist_fragment[kk][0] = 0;

dist_fragment[kk][1] = 0;

dist_fragment[kk][2] = 0;

dist_fragment[kk][3] = 0;

dist_fragment[kk][4] = 0;

}

for(kk=0;kk<total_R2_inactive_X_m;kk++){

fscanf(fR2_frag_X_inact_m,"%d %d %d %d %d", &R2_frag_size[0], &R2_frag_size[1], &R2_frag_size[2], &R2_frag_size[3], &R2_frag_size[4]);

dist_fragment[R2_frag_size[0]][0]++;

dist_fragment[R2_frag_size[1]][1]++;

dist_fragment[R2_frag_size[2]][2]++;

dist_fragment[R2_frag_size[3]][3]++;

dist_fragment[R2_frag_size[4]][4]++;

}

for(kk=0;kk<size_max+1;kk++){

fprintf(fdist_R2frag_X_inact_m,"%d\t%d\t%d\t%d\t%d\n", dist_fragment[kk][0], dist_fragment[kk][1], dist_fragment[kk][2], dist_fragment[kk][3], dist_fragment[kk][4]);

}

fclose(fR2_frag_X_inact_m); fclose(fdist_R2frag_X_inact_m);

printf("."); fflush(stdout);

//Distribution of R1frag_X.txt

FILE *fR1_frag_X=fopen("R1frag_X.txt","r");

FILE *fdist_R1frag_X=fopen("distribution_R1frag_X.txt","w");

for(kk=0;kk<size_max+1;kk++){

dist_fragment[kk][0] = 0;

dist_fragment[kk][1] = 0;

dist_fragment[kk][2] = 0;

dist_fragment[kk][3] = 0;

dist_fragment[kk][4] = 0;

}

for(kk=0;kk<(Nreplicate*(Npop*3/2));kk++){

fscanf(fR1_frag_X,"%d %d %d %d %d", &R1_frag_size[0], &R1_frag_size[1], &R1_frag_size[2], &R1_frag_size[3], &R1_frag_size[4]);

dist_fragment[R1_frag_size[0]][0]++;

dist_fragment[R1_frag_size[1]][1]++;

dist_fragment[R1_frag_size[2]][2]++;

dist_fragment[R1_frag_size[3]][3]++;

dist_fragment[R1_frag_size[4]][4]++;

}

for(kk=0;kk<size_max+1;kk++){

fprintf(fdist_R1frag_X,"%d\t%d\t%d\t%d\t%d\n", dist_fragment[kk][0], dist_fragment[kk][1], dist_fragment[kk][2], dist_fragment[kk][3], dist_fragment[kk][4]);

}

fclose(fR1_frag_X); fclose(fdist_R1frag_X);

printf("."); fflush(stdout);

//Distribution of R1frag_X_m.txt

FILE *fR1_frag_X_m=fopen("R1frag_X_m.txt","r");

FILE *fdist_R1frag_X_m=fopen("distribution_R1frag_X_m.txt","w");

for(kk=0;kk<size_max+1;kk++){

dist_fragment[kk][0] = 0;

dist_fragment[kk][1] = 0;

dist_fragment[kk][2] = 0;

dist_fragment[kk][3] = 0;

dist_fragment[kk][4] = 0;

}

for(kk=0;kk<(Nreplicate*(Npop/2));kk++){

fscanf(fR1_frag_X_m,"%d %d %d %d %d", &R1_frag_size[0], &R1_frag_size[1], &R1_frag_size[2], &R1_frag_size[3], &R1_frag_size[4]);

dist_fragment[R1_frag_size[0]][0]++;

dist_fragment[R1_frag_size[1]][1]++;

dist_fragment[R1_frag_size[2]][2]++;

dist_fragment[R1_frag_size[3]][3]++;

dist_fragment[R1_frag_size[4]][4]++;

}

for(kk=0;kk<size_max+1;kk++){

fprintf(fdist_R1frag_X_m,"%d\t%d\t%d\t%d\t%d\n", dist_fragment[kk][0], dist_fragment[kk][1], dist_fragment[kk][2], dist_fragment[kk][3], dist_fragment[kk][4]);

}

fclose(fR1_frag_X_m); fclose(fdist_R1frag_X_m);

printf("."); fflush(stdout);

//Distribution of R1frag_X_act.txt

FILE *fR1_frag_X_act=fopen("R1frag_X_act.txt","r");

FILE *fdist_R1frag_X_act=fopen("distribution_R1frag_X_act.txt","w");

for(kk=0;kk<size_max+1;kk++){

dist_fragment[kk][0] = 0;

dist_fragment[kk][1] = 0;

dist_fragment[kk][2] = 0;

dist_fragment[kk][3] = 0;

dist_fragment[kk][4] = 0;

}

for(kk=0;kk<total_R1_active_X;kk++){

fscanf(fR1_frag_X_act,"%d %d %d %d %d", &R1_frag_size[0], &R1_frag_size[1], &R1_frag_size[2], &R1_frag_size[3], &R1_frag_size[4]);

dist_fragment[R1_frag_size[0]][0]++;

dist_fragment[R1_frag_size[1]][1]++;

dist_fragment[R1_frag_size[2]][2]++;

dist_fragment[R1_frag_size[3]][3]++;

dist_fragment[R1_frag_size[4]][4]++;

}

for(kk=0;kk<size_max+1;kk++){

fprintf(fdist_R1frag_X_act,"%d\t%d\t%d\t%d\t%d\n", dist_fragment[kk][0], dist_fragment[kk][1], dist_fragment[kk][2], dist_fragment[kk][3], dist_fragment[kk][4]);

}

fclose(fR1_frag_X_act); fclose(fdist_R1frag_X_act);

printf("."); fflush(stdout);

//Distribution of R1frag_X_act_m.txt

FILE *fR1_frag_X_act_m=fopen("R1frag_X_act_m.txt","r");

FILE *fdist_R1frag_X_act_m=fopen("distribution_R1frag_X_act_m.txt","w");

for(kk=0;kk<size_max+1;kk++){

dist_fragment[kk][0] = 0;

dist_fragment[kk][1] = 0;

dist_fragment[kk][2] = 0;

dist_fragment[kk][3] = 0;

dist_fragment[kk][4] = 0;

}

for(kk=0;kk<total_R1_active_X_m;kk++){

fscanf(fR1_frag_X_act,"%d %d %d %d %d", &R1_frag_size[0], &R1_frag_size[1], &R1_frag_size[2], &R1_frag_size[3], &R1_frag_size[4]);

dist_fragment[R1_frag_size[0]][0]++;

dist_fragment[R1_frag_size[1]][1]++;

dist_fragment[R1_frag_size[2]][2]++;

dist_fragment[R1_frag_size[3]][3]++;

dist_fragment[R1_frag_size[4]][4]++;

}

for(kk=0;kk<size_max+1;kk++){

fprintf(fdist_R1frag_X_act_m,"%d\t%d\t%d\t%d\t%d\n", dist_fragment[kk][0], dist_fragment[kk][1], dist_fragment[kk][2], dist_fragment[kk][3], dist_fragment[kk][4]);

}

fclose(fR1_frag_X_act_m); fclose(fdist_R1frag_X_act_m);

printf("."); fflush(stdout);

//Distribution of R1frag_X_inact.txt

FILE *fR1_frag_X_inact=fopen("R1frag_X_inact.txt","r");

FILE *fdist_R1frag_X_inact=fopen("distribution_R1frag_X_inact.txt","w");

for(kk=0;kk<size_max+1;kk++){

dist_fragment[kk][0] = 0;

dist_fragment[kk][1] = 0;

dist_fragment[kk][2] = 0;

dist_fragment[kk][3] = 0;

dist_fragment[kk][4] = 0;

}

for(kk=0;kk<total_R1_inactive_X;kk++){

fscanf(fR1_frag_X_inact,"%d %d %d %d %d", &R1_frag_size[0], &R1_frag_size[1], &R1_frag_size[2], &R1_frag_size[3], &R1_frag_size[4]);

dist_fragment[R1_frag_size[0]][0]++;

dist_fragment[R1_frag_size[1]][1]++;

dist_fragment[R1_frag_size[2]][2]++;

dist_fragment[R1_frag_size[3]][3]++;

dist_fragment[R1_frag_size[4]][4]++;

}

for(kk=0;kk<size_max+1;kk++){

fprintf(fdist_R1frag_X_inact,"%d\t%d\t%d\t%d\t%d\n", dist_fragment[kk][0], dist_fragment[kk][1], dist_fragment[kk][2], dist_fragment[kk][3], dist_fragment[kk][4]);

}

fclose(fR1_frag_X_inact); fclose(fdist_R1frag_X_inact);

printf("."); fflush(stdout);

//Distribution of R1frag_X_inact_m.txt

FILE *fR1_frag_X_inact_m=fopen("R1frag_X_inact_m.txt","r");

FILE *fdist_R1frag_X_inact_m=fopen("distribution_R1frag_X_inact_m.txt","w");

for(kk=0;kk<size_max+1;kk++){

dist_fragment[kk][0] = 0;

dist_fragment[kk][1] = 0;

dist_fragment[kk][2] = 0;

dist_fragment[kk][3] = 0;

dist_fragment[kk][4] = 0;

}

for(kk=0;kk<total_R1_inactive_X_m;kk++){

fscanf(fR1_frag_X_inact_m,"%d %d %d %d %d", &R1_frag_size[0], &R1_frag_size[1], &R1_frag_size[2], &R1_frag_size[3], &R1_frag_size[4]);

dist_fragment[R1_frag_size[0]][0]++;

dist_fragment[R1_frag_size[1]][1]++;

dist_fragment[R1_frag_size[2]][2]++;

dist_fragment[R1_frag_size[3]][3]++;

dist_fragment[R1_frag_size[4]][4]++;

}

for(kk=0;kk<size_max+1;kk++){

fprintf(fdist_R1frag_X_inact_m,"%d\t%d\t%d\t%d\t%d\n", dist_fragment[kk][0], dist_fragment[kk][1], dist_fragment[kk][2], dist_fragment[kk][3], dist_fragment[kk][4]);

}

fclose(fR1_frag_X_inact_m); fclose(fdist_R1frag_X_inact_m);

printf("."); fflush(stdout);

//Distribution of R2frag_Y.txt

FILE *fR2_frag_Y=fopen("R2frag_Y.txt","r");

FILE *fdist_R2frag_Y=fopen("distribution_R2frag_Y.txt","w");

for(kk=0;kk<size_max+1;kk++){

dist_fragment[kk][0] = 0;

dist_fragment[kk][1] = 0;

dist_fragment[kk][2] = 0;

dist_fragment[kk][3] = 0;

dist_fragment[kk][4] = 0;

}

for(kk=0;kk<(Nreplicate*(Npop/2));kk++){

fscanf(fR2_frag_Y,"%d %d %d %d %d", &R2_frag_size[0], &R2_frag_size[1], &R2_frag_size[2], &R2_frag_size[3], &R2_frag_size[4]);

dist_fragment[R2_frag_size[0]][0]++;

dist_fragment[R2_frag_size[1]][1]++;

dist_fragment[R2_frag_size[2]][2]++;

dist_fragment[R2_frag_size[3]][3]++;

dist_fragment[R2_frag_size[4]][4]++;

}

for(kk=0;kk<size_max+1;kk++){

fprintf(fdist_R2frag_Y,"%d\t%d\t%d\t%d\t%d\n", dist_fragment[kk][0], dist_fragment[kk][1], dist_fragment[kk][2], dist_fragment[kk][3], dist_fragment[kk][4]);

}

fclose(fR2_frag_Y); fclose(fdist_R2frag_Y);

printf("."); fflush(stdout);

//Distribution of R2frag_Y_act.txt

FILE *fR2_frag_Y_act=fopen("R2frag_Y_act.txt","r");

FILE *fdist_R2frag_Y_act=fopen("distribution_R2frag_Y_act.txt","w");

for(kk=0;kk<size_max+1;kk++){

dist_fragment[kk][0] = 0;

dist_fragment[kk][1] = 0;

dist_fragment[kk][2] = 0;

dist_fragment[kk][3] = 0;

dist_fragment[kk][4] = 0;

}

for(kk=0;kk<total_R2_active_Y;kk++){

fscanf(fR2_frag_Y_act,"%d %d %d %d %d", &R2_frag_size[0], &R2_frag_size[1], &R2_frag_size[2], &R2_frag_size[3], &R2_frag_size[4]);

dist_fragment[R2_frag_size[0]][0]++;

dist_fragment[R2_frag_size[1]][1]++;

dist_fragment[R2_frag_size[2]][2]++;

dist_fragment[R2_frag_size[3]][3]++;

dist_fragment[R2_frag_size[4]][4]++;

}

for(kk=0;kk<size_max+1;kk++){

fprintf(fdist_R2frag_Y_act,"%d\t%d\t%d\t%d\t%d\n", dist_fragment[kk][0], dist_fragment[kk][1], dist_fragment[kk][2], dist_fragment[kk][3], dist_fragment[kk][4]);

}

fclose(fR2_frag_Y_act); fclose(fdist_R2frag_Y_act);

printf("."); fflush(stdout);

//Distribution of R2frag_Y_inact.txt

FILE *fR2_frag_Y_inact=fopen("R2frag_Y_inact.txt","r");

FILE *fdist_R2frag_Y_inact=fopen("distribution_R2frag_Y_inact.txt","w");

for(kk=0;kk<size_max+1;kk++){

dist_fragment[kk][0] = 0;

dist_fragment[kk][1] = 0;

dist_fragment[kk][2] = 0;

dist_fragment[kk][3] = 0;

dist_fragment[kk][4] = 0;

}

for(kk=0;kk<total_R2_inactive_Y;kk++){

fscanf(fR2_frag_Y_inact,"%d %d %d %d %d", &R2_frag_size[0], &R2_frag_size[1], &R2_frag_size[2], &R2_frag_size[3], &R2_frag_size[4]);

dist_fragment[R2_frag_size[0]][0]++;

dist_fragment[R2_frag_size[1]][1]++;

dist_fragment[R2_frag_size[2]][2]++;

dist_fragment[R2_frag_size[3]][3]++;

dist_fragment[R2_frag_size[4]][4]++;

}

for(kk=0;kk<size_max+1;kk++){

fprintf(fdist_R2frag_Y_inact,"%d\t%d\t%d\t%d\t%d\n", dist_fragment[kk][0], dist_fragment[kk][1], dist_fragment[kk][2], dist_fragment[kk][3], dist_fragment[kk][4]);

}

fclose(fR2_frag_Y_inact); fclose(fdist_R2frag_Y_inact);

printf("."); fflush(stdout);

//Distribution of R1frag_Y.txt

FILE *fR1_frag_Y=fopen("R1frag_Y.txt","r");

FILE *fdist_R1frag_Y=fopen("distribution_R1frag_Y.txt","w");

for(kk=0;kk<size_max+1;kk++){

dist_fragment[kk][0] = 0;

dist_fragment[kk][1] = 0;

dist_fragment[kk][2] = 0;

dist_fragment[kk][3] = 0;

dist_fragment[kk][4] = 0;

}

for(kk=0;kk<(Nreplicate*(Npop/2));kk++){

fscanf(fR1_frag_Y,"%d %d %d %d %d", &R1_frag_size[0], &R1_frag_size[1], &R1_frag_size[2], &R1_frag_size[3], &R1_frag_size[4]);

dist_fragment[R1_frag_size[0]][0]++;

dist_fragment[R1_frag_size[1]][1]++;

dist_fragment[R1_frag_size[2]][2]++;

dist_fragment[R1_frag_size[3]][3]++;

dist_fragment[R1_frag_size[4]][4]++;

}

for(kk=0;kk<size_max+1;kk++){

fprintf(fdist_R1frag_Y,"%d\t%d\t%d\t%d\t%d\n", dist_fragment[kk][0], dist_fragment[kk][1], dist_fragment[kk][2], dist_fragment[kk][3], dist_fragment[kk][4]);

}

fclose(fR1_frag_Y); fclose(fdist_R1frag_Y);

printf("."); fflush(stdout);

//Distribution of R1frag_Y_act.txt

FILE *fR1_frag_Y_act=fopen("R1frag_Y_act.txt","r");

FILE *fdist_R1frag_Y_act=fopen("distribution_R1frag_Y_act.txt","w");

for(kk=0;kk<size_max+1;kk++){

dist_fragment[kk][0] = 0;

dist_fragment[kk][1] = 0;

dist_fragment[kk][2] = 0;

dist_fragment[kk][3] = 0;

dist_fragment[kk][4] = 0;

}

for(kk=0;kk<total_R1_active_Y;kk++){

fscanf(fR1_frag_Y_act,"%d %d %d %d %d", &R1_frag_size[0], &R1_frag_size[1], &R1_frag_size[2], &R1_frag_size[3], &R1_frag_size[4]);

dist_fragment[R1_frag_size[0]][0]++;

dist_fragment[R1_frag_size[1]][1]++;

dist_fragment[R1_frag_size[2]][2]++;

dist_fragment[R1_frag_size[3]][3]++;

dist_fragment[R1_frag_size[4]][4]++;

}

for(kk=0;kk<size_max+1;kk++){

fprintf(fdist_R1frag_Y_act,"%d\t%d\t%d\t%d\t%d\n", dist_fragment[kk][0], dist_fragment[kk][1], dist_fragment[kk][2], dist_fragment[kk][3], dist_fragment[kk][4]);

}

fclose(fR1_frag_Y_act); fclose(fdist_R1frag_Y_act);

printf("."); fflush(stdout);

//Distribution of R1frag_Y_inact.txt

FILE *fR1_frag_Y_inact=fopen("R1frag_Y_inact.txt","r");

FILE *fdist_R1frag_Y_inact=fopen("distribution_R1frag_Y_inact.txt","w");

for(kk=0;kk<size_max+1;kk++){

dist_fragment[kk][0] = 0;

dist_fragment[kk][1] = 0;

dist_fragment[kk][2] = 0;

dist_fragment[kk][3] = 0;

dist_fragment[kk][4] = 0;

}

for(kk=0;kk<total_R1_inactive_Y;kk++){

fscanf(fR1_frag_Y_inact,"%d %d %d %d %d", &R1_frag_size[0], &R1_frag_size[1], &R1_frag_size[2], &R1_frag_size[3], &R1_frag_size[4]);

dist_fragment[R1_frag_size[0]][0]++;

dist_fragment[R1_frag_size[1]][1]++;

dist_fragment[R1_frag_size[2]][2]++;

dist_fragment[R1_frag_size[3]][3]++;

dist_fragment[R1_frag_size[4]][4]++;

}

for(kk=0;kk<size_max+1;kk++){

fprintf(fdist_R1frag_Y_inact,"%d\t%d\t%d\t%d\t%d\n", dist_fragment[kk][0], dist_fragment[kk][1], dist_fragment[kk][2], dist_fragment[kk][3], dist_fragment[kk][4]);

}

fclose(fR1_frag_Y_inact); fclose(fdist_R1frag_Y_inact);

printf("."); fflush(stdout);

printf(" Finished\n");

t3 = time(NULL);

printf("Simulation finished at %s", ctime(&t3));

printf("Total run time: ");

time_diff = (int)(difftime(t3, t1));

if(time_diff/3600 > 0){

printf(" %dhr",time_diff/3600);

}

printf(" %dmin %dsec\n\n", (time_diff%3600)/60, time_diff%60);

return 0;

}

/* Name: Locus Generator

* Input: s - char array that will hold the new locus

* size - the size, in units, that the new locus will be

* R2_level - the fraction of units that will be R2-inserted

* R1_level - the fraction of units that will be R1-inserted

*

* Output: n/a

* Function: used to fill a char array with a new locus

*

*/

void newLocus(char *s, int size, double R2_level, double R1_level){

int cc;

int window[20];

for(cc=0;cc<20;cc++){ window[cc]=size/20; }

for(cc=0;cc<size%20;cc++){ window[(int)(prob()*20)]++; }

char new_locus[unitSize*size_max];

char temp[unitSize*size_max/20];

strcpy(new_locus, "\0");

strcpy(temp, "\0");

int cc1, cc2;

double numR1, numR2;

for(cc1=0;cc1<20;cc1++){

strcpy(temp,"\0");

for(cc2=0;cc2<window[cc1];cc2++){

strcat(temp,"1");

for(cc=1;cc<unitSize;cc++){ strcat(temp,"-"); }

}

cc2 = 0;

if(ancester_random){

numR1 = size * R1_level * 0.05 * R1_present;

numR2 = size * R2_level * 0.05 * R2_present;

}

else{

numR1 = size * R1_level * R1dist[cc1] * R1_present;

numR2 = size * R2_level * R2dist[cc1] * R2_present;

}

while((numR1-cc2)>0 || (numR2-cc2)>0){

if(prob() < (numR1-cc2)){ R1transposition(temp); }

if(prob() < (numR2-cc2)){ R2transposition(temp); }

cc2++;

}

strcat(new_locus, temp);

}

strcat(new_locus, "\0");

transcribeNone(new_locus);

strcpy(s, new_locus);

}

/* Name: Uninserted Unit Counter

* Input: s - char array that represents the locus to be counted

* trans - flag for what part of the locus will be counted

* 0 = all units

* 1 = only transcribe units

*

* Output: number of uninserted units

* Function: used to count the number of uninserted units in the locus or transcribed region

*

*/

int countUn(char *s, int trans){

int length = strlen(s);int ii, counter = 0;

if(!R2_present && !R1_present){ return length/unitSize; }

for(ii=0;ii<length;ii+=unitSize){

if(!trans || s[ii]=='1'){

counter += uninsertedUnit(s, ii);

}

}

return counter;

}

/* Name: R2-inserted Unit Counter

* Input: s - char array that represents the locus to be counted

* trans - flag for what part of the locus will be counted

* 0 = all units

* 1 = only transcribe units

* trunc_code - determines if a truncated element is counted or not

* 0 - count all truncations

* 1 - count only truncations that will cut

* 2 - count only full-length elements

*

* Output: number of R2-inserted units (includes both single and double inserted units)

* Function: used to count the number of R2-inserted units in the locus or transcribed region

*

*/

int countR2(char *s, int trans, int trunc_code){

int length = strlen(s);

int ii, counter = 0;

if(!R2_present){ return 0; }

for(ii=0;ii<length;ii+=unitSize){

if(!trans || s[ii]=='1'){

counter += R2insertedUnit(s, ii, trunc_code);

}

}

return counter;

}

/* Name: R1-inserted Unit Counter

* Input: s - char array that represents the locus to be counted

* trans - flag for what part of the locus will be counted

* 0 = all units

* 1 = only transcribe units

* trunc_code - determines if a truncated element is counted or not

* 0 - count all truncations

* 1 - count only truncations that will cut

* 2 - count only full-length elements

*

* Output: number of R1-inserted units (includes both single and double inserted units)

* Function: used to count the number of R1-inserted units in the locus or transcribed region

*

*/

int countR1(char *s, int trans, int trunc_code){

int length = strlen(s);

int ii, counter = 0;

if(!R1_present){ return 0; }

for(ii=0;ii<length;ii+=unitSize){

if(!trans || s[ii]=='1'){

counter += R1insertedUnit(s, ii, trunc_code);

}

}

return counter;

}

/* Name: Double-inserted Unit Counter

* Input: s - char array that represents the locus to be counted

* trans - flag for what part of the locus will be counted

* 0 = all units

* 1 = only transcribe units

*

* Output: number of double-inserted units

* Function: used to count the number of double-inserted units in the locus or transcribed region

*

*/

int countDouble(char *s, int trans){

int length = strlen(s);

int ii, counter = 0;

if(!R2_present && !R1_present){ return 0; }

for(ii=0;ii<length;ii+=unitSize){

if(!trans || s[ii]=='1'){

counter += doubleInsertedUnit(s, ii);

}

}

return counter;

}

/* Name: R2-retrotransposition Event

* Input: s - char array that represents the locus that will have the R2-retrotransposition event

*

* Output: n/a

* Function: used to insert a new R2-element into a locus

*

*/

int R2transposition(char *s){

if(!R2_present){ return; }

int length = strlen(s);

int transcribe_length = length/unitSize;

int transcribe_start = 0;

if(transcribed_R2_retro){

transcribe_start = transcriptionStart(s, 0);

if(transcribe_start == -1){ return 0; }

transcribe_length = transcriptionLength(s, transcribe_start);

}

int ii, ii_temp, trunc, counter=0;

double stoc;

do{

ii = (int)(prob()*transcribe_length) * unitSize + transcribe_start;

if(R2_retro_stoc){

do{

stoc=Normal(R2_retro_stoc_normal_s);

ii_temp = (ii/unitSize + ((int)(stoc * (length/unitSize*2) + 0.5) - (length/unitSize))) * unitSize;

}while(ii_temp<0 || ii_temp>=length);

ii = ii_temp;

}

counter++;

if (counter>10*transcribe_length){

canceledR2retro++;

return 0;

}

}while((R2_picky && R1insertedUnit(s, ii, 0)) || R2insertedUnit(s, ii, 0) || (transcribed_R2_retro && !R2_retro_stoc && s[ii]=='0') || ii>=strlen(s));

s[ii+1] = '2';

s[ii+2] = '2';

if(prob()<R2_trunc_prob){

trunc = (int)(prob() * R2_trunc_num);

s[ii+1] = alphabet[trunc/strlen(alphabet)];

s[ii+2] = alphabet[trunc%strlen(alphabet)];

}

return 0;

}

/* Name: R1-retrotransposition Event

* Input: s - char array that represents the locus that will have the R1-retrotransposition event

*

* Output: n/a

* Function: used to insert a new R1-element into a locus

*

*/

int R1transposition(char *s){

if(!R1_present){ return; }

int length = strlen(s);

int transcribe_length = length/unitSize;

int transcribe_start = 0;

if(transcribed_R1_retro){

transcribe_start = transcriptionStart(s, 0);

if(transcribe_start == -1){ return 0; }

transcribe_length = transcriptionLength(s, transcribe_start);

}

int ii, ii_temp, trunc, counter=0;

double stoc;

do{

ii = (int)(prob()*transcribe_length) * unitSize + transcribe_start;

if(R1_retro_stoc){

do{

stoc=Normal(R1_retro_stoc_normal_s);

ii_temp = (ii/unitSize + ((int)(stoc * (length/unitSize*2) + 0.5) - (length/unitSize))) * unitSize;

}while(ii_temp<0 || ii_temp>=length);

ii = ii_temp;

}

counter++;

if (counter>10*transcribe_length){

canceledR1retro++;

return 0;

}

}while((R1_picky && R2insertedUnit(s, ii, 0)) || R1insertedUnit(s, ii, 0) || (transcribed_R1_retro && !R1_retro_stoc && s[ii]=='0') || ii>=strlen(s));

s[ii+1+(2*R2_present)] = '1';

s[ii+2+(2*R2_present)] = '1';

if(prob()<R1_trunc_prob){

trunc = (int)(prob() * R1_trunc_num);

s[ii+1+(2*R2_present)] = alphabet[trunc/strlen(alphabet)];

s[ii+2+(2*R2_present)] = alphabet[trunc%strlen(alphabet)];

}

return 0;

}

/* Name: Loop-deletion Event

* Input: s - char array that represents the locus that will have a loop-deletion event

*

* Output: n/a

* Function: deletes a section of the locus to simulate loop-deletion

*

*/

void loopDeletion(char *s){

int length = strlen(s);

int transcribe_length = length/unitSize;

int transcribe_start = 0;

if(transcribed_loop){

transcribe_start = transcriptionStart(s, 0);

if(transcribe_start == -1){ return; }

transcribe_length = transcriptionLength(s, transcribe_start);

}

if(transcribe_length < 2){ return; }

char loop_temp[unitSize*size_max];

strcpy(loop_temp,"\0");

int position, cc, temp_position;

double stoc;

int loop;

if (loop_size >= transcribe_length && !loop_stoc){

loop = (int)(1+prob()*(transcribe_length-1));

}else if(loop_size >= length/unitSize){

loop = (int)(1+prob()*(length/unitSize - 1));

}else{

loop = (int)(1+prob()*loop_size);

}

if(transcribe_length - loop <= 0){

position = transcribe_start - ((loop - transcribe_length)/2 * unitSize);

if(prob() < 0.5){ position-=unitSize;}

if(position < 0){ position = 0; }

if(position+(loop*unitSize) > length){ position = length-(loop*unitSize); }

}else{

position = (int)(prob()*(transcribe_length - loop + 1)) * unitSize + transcribe_start;

}

if(loop_stoc){

do{

stoc=Normal(loop_stoc_normal_s);

temp_position = (position/unitSize + ((int)(stoc * (length/unitSize*2) + 0.5) - (length/unitSize))) * unitSize;

}while(temp_position<0 || temp_position+(loop*unitSize)>=length);

position = temp_position;

}

for (cc=0;cc<position;cc++) {loop_temp[cc]=s[cc];}

for (cc=position;cc<length-(loop*unitSize);cc++) {loop_temp[cc]=s[cc+(loop*unitSize)];}

loop_temp[cc]='\0';

strcpy(s,loop_temp);

}

/* Name: SCE Recombination Event

* Input: s - char array that represents the locus that will undergo SCE recombination

*

* Output: n/a

* Function: simulates an SCE event in a locus

*

*/

void sischr(char *s){

int length = strlen(s);

int transcribe_length = length/unitSize;

int transcribe_start = 0;

if(transcribed_SCE_recomb){

transcribe_start = transcriptionStart(s, 0);

if(transcribe_start == -1){ return; }

transcribe_length = transcriptionLength(s, transcribe_start);

}

if(transcribe_length < 2){ return; }

char l1[unitSize*size_max];

char l2[unitSize*size_max];

strcpy(l1, "\0");

strcpy(l2, "\0");

int cc, stagger = 0;

if(SCE_stagger >= transcribe_length){

stagger = rStagger(transcribe_length - 1);

}

else{

stagger = rStagger(SCE_stagger);

}

int positions[size_max];

int position = -1, temp_position, Nmatch = 0, test, pos, counter;

double stoc;

double k = -1;

if (transcribe_length<(stagger/unitSize)){

canceledSCE++;

}else{

if(matching_SCE_recomb){

/*

* Nmatch=0;

* for(cc=(required_matching_SCE*unitSize);cc<length-stagger-(required_matching_SCE*unitSize);cc+=unitSize){

* test = 1;

* pos = cc - (unitSize * required_matching_SCE);

* while(pos < (cc + ((required_matching_SCE+1)*unitSize)) && test){

* if(s[cc] != s[cc + stagger]){ test = 0; }

* pos++;

* }

* if(test){

* positions[Nmatch]=cc;

* Nmatch++;

* }

* }

* if(Nmatch > 0){

* position = positions[(int)(prob()*Nmatch)];

* if(prob() < 0.5){ position+=unitSize; }

* }

*/

}

else{

if(SCE_random){

k = prob();

}

else{

k = Normal(SCE_normal_s);

}

position = ((int)(k*((transcribe_length) - (stagger/unitSize) + 1)) * unitSize) + transcribe_start;

if(SCE_stoc){

do{

stoc=Normal(SCE_stoc_normal_s);

temp_position = (position/unitSize + ((int)(stoc * (length/unitSize*2) + 0.5) - (length/unitSize))) * unitSize;

}while(temp_position<0 || temp_position+stagger>length);

position = temp_position;

}

}

if(position != -1){

counter = 0;

for (cc=0;cc<position;cc++){ l1[counter] = s[cc]; counter++;}

for (cc=position+stagger;cc<length;cc++){

if(counter>=(size_max*unitSize)){

canceledSCE++;

return;

}

l1[counter] = s[cc];

counter++;

}

l1[counter]='\0';

counter = 0;

for (cc=0;cc<position+stagger;cc++){ l2[counter] = s[cc]; counter++;}

for (cc=position;cc<length;cc++){

if(counter>=(size_max*unitSize)){

canceledSCE++;

return;

}

l2[counter] = s[cc];

counter++;

}

l2[counter]='\0';

if(prob()<0.5){strcpy(s,l1);}

else{strcpy(s,l2);}

}

}

}

/* Name: ICE Recombination Event

* Input: s1 - first locus that will be involved in the ICE recombination

* s2 - second locus involved

*

* Output: n/a

* Function: simulates an ICE event in a pair of loci

*

*/

void interce(char *s1,char *s2){

int length1=strlen(s1), length2=strlen(s2);

int transcribe_length1 = length1/unitSize;

int transcribe_length2 = length2/unitSize;

int transcribe_start1 = 0;

int transcribe_start2 = 0;

if(transcribed_ICE_recomb){

transcribe_start1 = transcriptionStart(s1, 0);

if(transcribe_start1 == -1){ return; }

transcribe_length1 = transcriptionLength(s1, transcribe_start1);

transcribe_start2 = transcriptionStart(s2, 0);

if(transcribe_start2 == -1){ return; }

transcribe_length2 = transcriptionLength(s2, transcribe_start2);

}

if(transcribe_length1 < 2){ return; }

if(transcribe_length2 < 2){ return; }

char l1[unitSize*size_max];

char l2[unitSize*size_max];

strcpy(l1, "\0");

strcpy(l2, "\0");

double k;

int positions[size_max];

int stagger, diff, rdiff, left, right, test, Nmatch = 0, position, pos1 = -1, pos2 = -1, cc, counter, temp_pos1, temp_pos2;

double stoc;

if(transcribe_length1 <= transcribe_length2){

diff = ((transcribe_length2 - transcribe_length1) / 2) * unitSize;

rdiff = diff;

if((transcribe_length2-transcribe_length1)%2 == 1){

if(prob()<0.5){ diff+=unitSize; }

else{ rdiff+=unitSize;}

}

if(ICE_stagger >= (transcribe_length1 + ((transcribe_length2-transcribe_length1)/2))){

stagger = rStagger(transcribe_length1 + ((transcribe_length2-transcribe_length1)/2) - 1);

}

else{

stagger = rStagger(ICE_stagger);

}

if(prob()<0.5){ stagger = stagger * -1; }

if(stagger + diff < 0){

left = transcribe_start1 - (diff + stagger);

right = transcribe_start1 + (transcribe_length1 * unitSize);

}else if (stagger > rdiff){

left = transcribe_start1;

right = transcribe_start1 + (transcribe_length1 * unitSize) - (stagger - rdiff);

}else{

left = transcribe_start1;

right = transcribe_start1 + (transcribe_length1 * unitSize);

}

if(matching_ICE_recomb){

/*

* Nmatch=0;

* for(cc=left+(required_matching_ICE*unitSize);cc<right-(required_matching_ICE*unitSize);cc+=unitSize){

* test = 1;

* position = cc - (unitSize * required_matching_ICE);

* while(position < (cc + ((required_matching_ICE+1)*unitSize)) && test){

* if(s1[cc] != s2[cc + diff + stagger]){ test = 0; }

* position++;

* }

* if(test){

* positions[Nmatch]=cc;

* Nmatch++;

* }

* }

* if(Nmatch > 0){

* pos1 = positions[(int)(prob()*Nmatch)];

* if(prob() < 0.5){ pos1+=unitSize; }

* }

*/

}

else{

if(ICE_random){

k = prob();

}

else{

k = Normal(ICE_normal_s);

}

pos1 = ((int)(k*((right - left)/unitSize + 1)) * unitSize) + left;

}

pos2 = pos1 + diff + stagger + transcribe_start2 - transcribe_start1;

}

else{

diff = ((transcribe_length1 - transcribe_length2) / 2) * unitSize;

rdiff = diff;

if((transcribe_length1-transcribe_length2)%2 == 1){

if(prob()<0.5){ diff+=unitSize; }

else{ rdiff+=unitSize;}

}

if(ICE_stagger >= (transcribe_length2 + ((transcribe_length1-transcribe_length2)/2))){

stagger = rStagger(transcribe_length2 + ((transcribe_length1-transcribe_length2)/2) - 1);

}

else{

stagger = rStagger(ICE_stagger);

}

if(prob()<0.5){ stagger = stagger * -1; }

if(stagger + diff < 0){

left = transcribe_start2 - (diff + stagger);

right = transcribe_start2 + (transcribe_length2 * unitSize);

}else if (stagger > rdiff){

left = transcribe_start2;

right = transcribe_start2 + (transcribe_length2 * unitSize) - (stagger - rdiff);

}else{

left = transcribe_start2;

right = transcribe_start2 + (transcribe_length2 * unitSize);

}

if(matching_ICE_recomb){

/*

* Nmatch=0;

* for(cc=left+(required_matching_ICE*unitSize);cc<right-(required_matching_ICE*unitSize);cc+=unitSize){

* test = 1;

* position = cc - (unitSize * required_matching_ICE);

* while(position < (cc + ((required_matching_ICE+1)*unitSize)) && test){

* if(s2[cc] != s1[cc + diff + stagger]){ test = 0; }

* position++;

* }

* if(test){

* positions[Nmatch]=cc;

* Nmatch++;

* }

* }

* if(Nmatch > 0){

* pos2 = positions[(int)(prob()*Nmatch)];

* if(prob() < 0.5){ pos1+=unitSize; }

* }

*/

}

else{

if(ICE_random){

k = prob();

}

else{

k = Normal(ICE_normal_s);

}

pos2 = ((int)(k*((right - left)/unitSize + 1)) * unitSize) + left;

}

pos1 = pos2 + diff + stagger + transcribe_start1 - transcribe_start2;

}

if(ICE_stoc){

if(length1 <= length2){

do{

stoc=Normal(ICE_stoc_normal_s);

temp_pos1 = (pos1/unitSize + ((int)(stoc * (length1/unitSize*2) + 0.5) - (length1/unitSize))) * unitSize;

temp_pos2 = pos2 + (pos1 - temp_pos1);

}while(temp_pos1<0 || temp_pos1>length1 || temp_pos2<0 || temp_pos2>length2);

pos1 = temp_pos1;

pos2 = temp_pos2;

}

else{

do{

stoc=Normal(ICE_stoc_normal_s);

temp_pos2 = (pos2/unitSize + ((int)(stoc * (length2/unitSize*2) + 0.5) - (length2/unitSize))) * unitSize;

temp_pos1 = pos1 + (pos2 - temp_pos2);

}while(temp_pos1<0 || temp_pos1>length1 || temp_pos2<0 || temp_pos2>length2);

pos1 = temp_pos1;

pos2 = temp_pos2;

}

}

if(pos1 != -1 && pos2 != -1){

counter = 0;

for(cc=0;cc<pos1;cc++){ l1[counter]=s1[cc]; counter++; }

for(cc=pos2;cc<length2;cc++){

if(counter>=(size_max*unitSize)){

canceledICE++;

return;

}

l1[counter]=s2[cc];

counter++;

}

l1[counter] = '\0';

counter = 0;

for(cc=0;cc<pos2;cc++){ l2[counter]=s2[cc]; counter++; }

for(cc=pos1;cc<length1;cc++){

if(counter>=(size_max*unitSize)){

canceledICE++;

return;

}

l2[counter]=s1[cc];

counter++;

}

l2[counter] = '\0';

strcpy(s1,l1);strcpy(s2,l2);

}

}

/* Name: Transcribe None

* Input: s - char array that will be changed

*

* Output: n/a

* Function: changes the locus so that none of the units will be transcribed

*

*/

void transcribeNone(char *s){

int ii, length = strlen(s);

for(ii=0;ii<length;ii+=unitSize){

s[ii] = '0';

}

}

/* Name: Transcribe All

* Input: s - char array that will be changed

*

* Output: n/a

* Function: changes the locus so that all of the units will be transcribed

*

*/

void transcribeAll(char *s){

int ii, length = strlen(s);

for(ii=0;ii<length;ii+=unitSize){

s[ii] = '1';

}

}

/* Name: Transcribe Area

* Input: s - char array that will be changed

* size - the number of units to transcribe

* R2memory - determines whether the locus can recognize the R2-element (positive = yes, 0 or negative = no)

* R1memory - determines whether the locus can recognize the R2-element (positive = yes, 0 or negative = no)

*

* Output: n/a

* Function: changes the locus so that an area of the locus centered on the largest continuous stretch of uninserted units will be transcribed

*

*/

void transcribeArea(char *s, int size, int R2memory, int R1memory){

int length = strlen(s);

if((length/unitSize) <= size){

transcribeAll(s);

return;

}

int ii, pos = -1, max = 0;

int numPos = 1;

int count = 0, curr_pos = -1;

for(ii = 0; ii < length; ii+=unitSize){

if((!R2_present || R2memory<=0 || !R2insertedUnit(s,ii,0)) && (!R1_present || R1memory<=0 || !R1insertedUnit(s,ii,0))){

if(count == 0){

curr_pos = ii;

}

count++;

}

else{

if(curr_pos != -1 && (count > max || (count == max && prob() < (float)(1.0/numPos)))){

pos = curr_pos;

max = count;

if(count == max){numPos++;}

else{numPos=1;}

}

count = 0;

curr_pos = -1;

}

}

if(curr_pos != -1 && (count > max || (count == max && prob() < (float)(1.0/numPos)))){

pos = curr_pos;

max = count;

}

if(pos==-1){

pos = (int)(prob()*((length/unitSize) - size + 1))*unitSize;

}

if(max > size){

pos = (pos + ((int)(prob()*(max - size + 1))*unitSize));

}

else if(max < size){

pos = pos - (((size - max) / 2)*unitSize);

if((size - max) % 2 != 0 && prob() < 0.5){pos = pos - unitSize;}

if(pos + (size * unitSize) > length){pos = length - (size * unitSize);}

if(pos < 0){pos=0;}

}

transcribeNone(s);

if(transcription_stoc){

int new_pos;

double stoc;

do{

stoc=Normal(trStoc_normal_s);

new_pos = (pos/unitSize + ((int)(stoc * (length/unitSize*2) + 0.5) - (length/unitSize))) * unitSize;

}while((new_pos + (size * unitSize) > length) || (new_pos < 0));

pos = new_pos;

}

for(ii=pos;ii<(pos+(size*unitSize));ii+=unitSize){ s[ii]='1'; }

}

/* Name: Nuclear Dominance

* Input: s1 - first locus to be involved

* s2 - second locus involved

*

* Output: n/a

* Function: changes the size of the transcribed regions of the loci to minimize the number of transcribed elements

* 1) if both transcribed regions are either inserted or uninserted, then nothing is changed

* 2) if one region is uninserted and the other is inserted, then the size of the inserted region decreases by one (favoring inserted units),

* and the size of the uninserted region increases by one (favoring uninserted units)

* 3) this continues until both regions are either inserted or uninserted or the size of one region is reduced to zero

*

*/

void nuclearDominance(char *s1, char *s2){

int length1=strlen(s1), length2=strlen(s2);

int transcribe_start1 = transcriptionStart(s1, 0);

if(transcribe_start1 == -1){ return; }

int transcribe_length1 = transcriptionLength(s1, transcribe_start1);

int transcribe_start2 = transcriptionStart(s2, 0);

if(transcribe_start2 == -1){ return; }

int transcribe_length2 = transcriptionLength(s2, transcribe_start2);

if(countUn(s1,1)==transcribe_length1 && countUn(s2,1)<transcribe_length2 && transcribe_length1<length1/unitSize){

do{

if(!uninsertedUnit(s2,transcribe_start2) && uninsertedUnit(s2,transcribe_start2+((transcribe_length2-1)*unitSize))){

s2[transcribe_start2]='0';

transcribe_start2+=unitSize;

transcribe_length2--;

}

else if(uninsertedUnit(s2,transcribe_start2) && !uninsertedUnit(s2,transcribe_start2+((transcribe_length2-1)*unitSize))){

s2[transcribe_start2+((transcribe_length2-1)*unitSize)]='0';

transcribe_length2--;

}

else if(prob()<0.5){

s2[transcribe_start2]='0';

transcribe_start2+=unitSize;

transcribe_length2--;

}

else{

s2[transcribe_start2+((transcribe_length2-1)*unitSize)]='0';

transcribe_length2--;

}

if(!uninsertedUnit(s1,transcribe_start1-unitSize) && uninsertedUnit(s1,transcribe_start1+(transcribe_length1*unitSize)) && transcribe_start1+(transcribe_length1*unitSize)<length1){

s1[transcribe_start1+(transcribe_length1*unitSize)]='1';

transcribe_length1++;

}

else if(uninsertedUnit(s1,transcribe_start1-unitSize) && !uninsertedUnit(s1,transcribe_start1+(transcribe_length1*unitSize)) && transcribe_start1-unitSize>=0){

s1[transcribe_start1-unitSize]='1';

transcribe_start1-=unitSize;

transcribe_length1++;

}

else if((prob()<0.5 && transcribe_start1+(transcribe_length1*unitSize)<length1) || transcribe_start1-unitSize<0){

s1[transcribe_start1+(transcribe_length1*unitSize)]='1';

transcribe_length1++;

}

else{

s1[transcribe_start1-unitSize]='1';

transcribe_start1-=unitSize;

transcribe_length1++;

}

}while(countUn(s1,1)==transcribe_length1 && countUn(s2,1)<transcribe_length2 && transcribe_length1<length1/unitSize);

}

else if(countUn(s1,1)<transcribe_length1 && countUn(s2,1)==transcribe_length2 && transcribe_length2<length2/unitSize){

do{

if(!uninsertedUnit(s1,transcribe_start1) && uninsertedUnit(s1,transcribe_start1+((transcribe_length1-1)*unitSize))){

s1[transcribe_start1]='0';

transcribe_start1+=unitSize;

transcribe_length1--;

}

else if(uninsertedUnit(s1,transcribe_start1) && !uninsertedUnit(s1,transcribe_start1+((transcribe_length1-1)*unitSize))){

s1[transcribe_start1+((transcribe_length1-1)*unitSize)]='0';

transcribe_length1--;

}

else if(prob()<0.5){

s1[transcribe_start1]='0';

transcribe_start1+=unitSize;

transcribe_length1--;

}

else{

s1[transcribe_start1+((transcribe_length1-1)*unitSize)]='0';

transcribe_length1--;

}

if(!uninsertedUnit(s2,transcribe_start2-unitSize) && uninsertedUnit(s2,transcribe_start2+(transcribe_length2*unitSize)) && transcribe_start2+(transcribe_length2*unitSize)<length2){

s2[transcribe_start2+(transcribe_length2*unitSize)]='1';

transcribe_length2++;

}

else if(uninsertedUnit(s2,transcribe_start2-unitSize) && !uninsertedUnit(s2,transcribe_start2+(transcribe_length2*unitSize)) && transcribe_start2-unitSize>=0){

s2[transcribe_start2-unitSize]='1';

transcribe_start2-=unitSize;

transcribe_length2++;

}

else if((prob()<0.5 && transcribe_start2+(transcribe_length2*unitSize)<length2) || transcribe_start2-unitSize<0){

s2[transcribe_start2+(transcribe_length2*unitSize)]='1';

transcribe_length2++;

}

else{

s2[transcribe_start2-unitSize]='1';

transcribe_start2-=unitSize;

transcribe_length2++;

}

}while(countUn(s1,1)<transcribe_length1 && countUn(s2,1)==transcribe_length2 && transcribe_length2<length2/unitSize);

}

}

/* Name: Find Transcription Start

* Input: s - char array to be evaluated

* first - the first unit to start searching from

*

* Output: the position in the char array that represents the first transcribed unit the function finds

* Function: searches from the first unit indicated to the end of the locus for the first transcribed region

*

*/

int transcriptionStart(char *s, int first){

int ii, length = strlen(s);

for(ii=first; ii<length; ii+=unitSize){

if(s[ii]=='1'){ return ii; }

}

return -1;

}

/* Name: Transcription Length Determiner

* Input: s - char array to be evaluated

* start - the position in the char array that represents in the first unit in the transcribed region

*

* Output: the number of units in the transcribed region

* Function: determines the size of the transcribed region

*

*/

int transcriptionLength(char *s, int start){

int ii, length = strlen(s), counter = 0;

for(ii=start; ii<length; ii+=unitSize){

if(s[ii]=='1'){ counter++; }

else{ return counter; }

}

return counter;

}

/* Name: Uninserted Unit Determiner

* Input: s - char array to be evaluated

* start - the position in the char array that represents the unit to be evaluated

*

* Output: returns 0 fn the unit is inserted or 1 if the unit is uninserted

* Function: determines whether the specified unit is uninserted or not

*

*/

int uninsertedUnit(char *s, int unit){

if((R2_present && s[unit+(R2_present)]!='-' && s[unit+(R2_present*2)]!='-') || (R1_present && s[unit+(R2_present*2)+(R1_present)]!='-' && s[unit+(R2_present*2)+(R1_present*2)]!='-')){

return 0;

}

return 1;

}

/* Name: R2-inserted Unit Determiner

* Input: s - char array to be evaluated

* start - the position in the char array that represents the unit to be evaluated

* trunc_code - determines if a truncated element is counted or not

* 0 - count all truncations

* 1 - count only truncations that will cut

* 2 - count only full-length elements

*

* Output: returns 1 in the unit is R2-inserted or 0 if the unit has no R2

* Function: determines whether the specified unit is R2-inserted or not

*

*/

int R2insertedUnit(char *s, int unit, int trunc_code){

if(trunc_code == 0){

if(R2_present && s[unit+(R2_present)]!='-' && s[unit+(R2_present*2)]!='-'){

return 1;

}

}

else if(trunc_code == 1){

if(R2_present && s[unit+(R2_present)]!='-' && s[unit+(R2_present*2)]!='-' && trunc_num(s[unit+(R2_present)],s[unit+(R2_present*2)])<last_R2_cut){

return 1;

}

}

else{

if(R2_present && s[unit+(R2_present)]=='2' && s[unit+(R2_present*2)]=='2'){

return 1;

}

}

return 0;

}

/* Name: R1-inserted Unit Determiner

* Input: s - char array to be evaluated

* start - the position in the char array that represents the unit to be evaluated

* trunc_code - determines if a truncated element is counted or not

* 0 - count all truncations

* 1 - count only truncations that will cut

* 2 - count only full-length elements

*

* Output: returns 1 in the unit is R1-inserted or 0 if the unit has no R1

* Function: determines whether the specified unit is R1-inserted or not

*

*/

int R1insertedUnit(char *s, int unit, int trunc_code){

if(trunc_code == 0){

if(R1_present && s[unit+(R2_present*2)+(R1_present)]!='-' && s[unit+(R2_present*2)+(R1_present*2)]!='-'){

return 1;

}

}

else if (trunc_code == 1){

if(R1_present && s[unit+(R2_present*2)+(R1_present)]!='-' && s[unit+(R2_present*2)+(R1_present*2)]!='-' && trunc_num(s[unit+(R2_present*2)+(R1_present)],s[unit+(R2_present*2)+(R1_present*2)])<last_R1_cut){

return 1;

}

}

else{

if(R1_present && s[unit+(R2_present*2)+(R1_present)]=='1' && s[unit+(R2_present*2)+(R1_present*2)]=='1'){

return 1;

}

}

return 0;

}

/* Name: Double-inserted Unit Determiner

* Input: s - char array to be evaluated

* start - the position in the char array that represents the unit to be evaluated

*

* Output: returns 1 in the unit is R2-inserted and R1-inserted or 0 if the unit is uninserted

* Function: determines whether the specified unit is double-inserted or not

*

*/

int doubleInsertedUnit(char *s, int unit){

if((R2_present && s[unit+(R2_present)]!='-' && s[unit+(R2_present*2)]!='-') && (R1_present && s[unit+(R2_present*2)+(R1_present)]!='-' && s[unit+(R2_present*2)+(R1_present*2)]!='-')){

return 1;

}

return 0;

}

/* Name: Female Offspring Calculator

* Input: unCountX1 - number of transcribed uninserted units in the first X locus

* unCountX2 - number of transcribed uninserted units in the second X locus

* activity - determines if fly is active (0 = inactive, >0 = active)

*

* Output: returns the number of offspring loci

* Function: determines the number of offspring loci a female fly will produce

*

*/

int numOffspringF(int unCountX1, int unCountX2, int activity){

int temp = unCountX1 + unCountX2;

double fitness;

if(!flag_selection){ fitness=1.0; }

if(temp>=w2_f){ fitness=1.0; }

else if(temp<w1_f){ fitness=0.0; }

else{ fitness=((temp-w1_f)/((float)(w2_f-w1_f))); }

if(activity > 0){ fitness = fitness - act_fitPen; }

if(fitness < 0){ fitness = 0.0; }

int numberOfOffspring = (int)(fecundity * fitness);

if(prob() < (fecundity*fitness-numberOfOffspring) && numberOfOffspring<fecundity){ numberOfOffspring++; }

return numberOfOffspring;

}

/* Name: Male Offspring Calculator

* Input: unCountX - number of transcribed uninserted units in the X locus

* unCountY - number of transcribed uninserted units in the Y locus

* activity - determines if fly is active (0 = inactive, >0 = active)

*

* Output: returns the number of offspring loci

* Function: determines the number of offspring loci a male fly will produce

*

*/

int numOffspringM(int unCountX, int unCountY, int activity){

int temp;

if(Y_dominance == -1){ temp=unCountX; }

if(Y_dominance == 1){ temp=unCountY; }

else{ temp=unCountX+unCountY; }

double fitness;

if(!flag_selection){ fitness=1.0; }

if(temp>=w2_m){ fitness=1.0; }

else if(temp<w1_m){ fitness=0.0; }

else{ fitness=((temp-w1_m)/((float)(w2_m-w1_m))); }

if(activity > 0){ fitness = fitness - act_fitPen; }

if(fitness < 0){ fitness = 0.0; }

int numberOfOffspring = (int)(fecundity * fitness);

if(prob() < (fecundity*fitness-numberOfOffspring) && numberOfOffspring<fecundity){ numberOfOffspring++; }

return numberOfOffspring;

}

/* Name: Sliding Window Analyzer

* Input: s - the locus to be analyzed

* XYflag - flag for whether the locus is on the X or Y chromosome

* 'X' = X chromosome

* 'Y' = Y chromosome

* transcribed_R2 - the number of transcribed R2 elements

* transcribed_R1 - the number of transcribed R1 elements

*

* Output: n/a

* Function: preforms a sliding window analysis of the specified locus

*

*/

void sldwin(char *s, char XYflag, int transcribed_R2, int transcribed_R1){

int length = strlen(s)/unitSize;

int window[20], cc1, cc2;

int CurrPos=0;

for(cc1=0;cc1<20;cc1++){ window[cc1]=length/20; }

for(cc1=0;cc1<length%20;cc1++){

window[(int)(prob()*20)]++;

}

if(XYflag=='X'){

for(cc1=0;cc1<20;cc1++){

for(cc2=0;cc2<window[cc1];cc2++){

if(uninsertedUnit(s, CurrPos)){

UNsldwin_X[cc1]++;

if(transcribed_R2 > 0 || transcribed_R1 > 0){

UNsldwin_X_act[cc1]++;

}

else{

UNsldwin_X_inact[cc1]++;

}

}

if(R2insertedUnit(s, CurrPos, 0)){

R2sldwin_X[cc1]++;

if(transcribed_R2 > 0 || transcribed_R1 > 0){

R2sldwin_X_act[cc1]++;

}

else{

R2sldwin_X_inact[cc1]++;

}

}

if(R1insertedUnit(s, CurrPos, 0)){

R1sldwin_X[cc1]++;

if(transcribed_R2 > 0 || transcribed_R1 > 0){

R1sldwin_X_act[cc1]++;

}

else{

R1sldwin_X_inact[cc1]++;

}

}

if(s[CurrPos]=='1'){

TRsldwin_X[cc1]++;

if(transcribed_R2 > 0 || transcribed_R1 > 0){

TRsldwin_X_act[cc1]++;

}

else{

TRsldwin_X_inact[cc1]++;

}

}

CurrPos+=unitSize;

}

}

}

else if(XYflag=='Y'){

for(cc1=0;cc1<20;cc1++){

for(cc2=0;cc2<window[cc1];cc2++){

if(uninsertedUnit(s, CurrPos)){

UNsldwin_Y[cc1]++;

if(transcribed_R2 > 0 || transcribed_R1 > 0){

UNsldwin_Y_act[cc1]++;

}

else{

UNsldwin_Y_inact[cc1]++;

}

}

if(R2insertedUnit(s, CurrPos, 0)){

R2sldwin_Y[cc1]++;

if(transcribed_R2 > 0 || transcribed_R1 > 0){

R2sldwin_Y_act[cc1]++;

}

else{

R2sldwin_Y_inact[cc1]++;

}

}

if(R1insertedUnit(s, CurrPos, 0)){

R1sldwin_Y[cc1]++;

if(transcribed_R2 > 0 || transcribed_R1 > 0){

R1sldwin_Y_act[cc1]++;

}

else{

R1sldwin_Y_inact[cc1]++;

}

}

if(s[CurrPos]=='1'){

TRsldwin_Y[cc1]++;

if(transcribed_R2 > 0 || transcribed_R1 > 0){

TRsldwin_Y_act[cc1]++;

}

else{

TRsldwin_Y_inact[cc1]++;

}

}

CurrPos+=unitSize;

}

}

}

}

/* Name: Truncated Element Abundance Analyzer

* Input: s - the locus to be analyzed

* XYflag - flag for whether the locus is on the X or Y chromosome

* 'X' = X chromosome

* 'Y' = Y chromosome

* transcribed_R2 - the number of transcribed R2 elements

* transcribed_R1 - the number of transcribed R1 elements

*

* Output: n/a

* Function: calculates the abundance of every truncated element in the locus

*

*/

void trunc_abundance(char *s, char XYflag, int transcribed_R2, int transcribed_R1){

int R1_trunc[R1_trunc_num];

int R2_trunc[R2_trunc_num];

int cc, cc1, cc2;

for(cc=0;cc<R1_trunc_num;cc++) {R1_trunc[cc]=0;}

for(cc=0;cc<R2_trunc_num;cc++) {R2_trunc[cc]=0;}

for(cc=0;cc<strlen(s);cc+=unitSize){

if(R1insertedUnit(s, cc, 0)){

if(s[cc+(R2_present*2)+(R1_present)]!='1' || s[cc+(R2_present*2)+(R1_present*2)]!='1'){

for(cc1=0;cc1<26;cc1++){

for(cc2=0;cc2<26;cc2++){

if(s[cc+(R2_present*2)+(R1_present)]==alphabet[cc1] && s[cc+(R2_present*2)+(R1_present*2)]==alphabet[cc2]){

R1_trunc[(cc1*26)+cc2]++;

}

}

}

}

}

if(R2insertedUnit(s, cc, 0)){

if(s[cc+(R2_present)]!='2' || s[cc+(R2_present*2)]!='2'){

for(cc1=0;cc1<26;cc1++){

for(cc2=0;cc2<26;cc2++){

if(s[cc+(R2_present)]==alphabet[cc1] && s[cc+(R2_present*2)]==alphabet[cc2]){

R2_trunc[(cc1*26)+cc2]++;

}

}

}

}

}

}

if(XYflag=='X'){

for(cc=0;cc<R1_trunc_num;cc++){

R1_trunc_copy_X[R1_trunc[cc]]++;

if(transcribed_R2>0 || transcribed_R1>0){

R1_trunc_copy_X_act[R1_trunc[cc]]++;

}

else{

R1_trunc_copy_X_inact[R1_trunc[cc]]++;

}

if(R1_trunc[cc] > R1_trunc_max_X){ R1_trunc_max_X=R1_trunc[cc]; }

}

for(cc=0;cc<R2_trunc_num;cc++){

R2_trunc_copy_X[R2_trunc[cc]]++;

if(transcribed_R2>0 || transcribed_R1>0){

R2_trunc_copy_X_act[R2_trunc[cc]]++;

}

else{

R2_trunc_copy_X_inact[R2_trunc[cc]]++;

}

if(R2_trunc[cc] > R2_trunc_max_X){ R2_trunc_max_X=R2_trunc[cc]; }

}

}

else if(XYflag=='Y'){

for(cc=0;cc<R1_trunc_num;cc++){

R1_trunc_copy_Y[R1_trunc[cc]]++;

if(transcribed_R2>0 || transcribed_R1>0){

R1_trunc_copy_Y_act[R1_trunc[cc]]++;

}

else{

R1_trunc_copy_Y_inact[R1_trunc[cc]]++;

}

if(R1_trunc[cc] > R1_trunc_max_Y){ R1_trunc_max_Y=R1_trunc[cc]; }

}

for(cc=0;cc<R2_trunc_num;cc++){

R2_trunc_copy_Y[R2_trunc[cc]]++;

if(transcribed_R2>0 || transcribed_R1>0){

R2_trunc_copy_Y_act[R2_trunc[cc]]++;

}

else{

R2_trunc_copy_Y_inact[R2_trunc[cc]]++;

}

if(R2_trunc[cc] > R2_trunc_max_Y){ R2_trunc_max_Y=R2_trunc[cc]; }

}

}

}

/* Name: Fragment size analyzer

* Input: s - the locus to be analyzed

*

* Output: a double in the range of 0≤X<0

* Function: determines the distance between elements of the largest fragment

*

*/

void fragment_size(char *s){

int length = strlen(s)/unitSize;

int R2_dist=1, R1_dist=1;

int cc;

for(cc=0;cc<5;cc++){

R2_frag_size[cc] = 0;

R1_frag_size[cc] = 0;

}

for(cc=0;cc<strlen(s);cc+=unitSize){

if(R2insertedUnit(s, cc, 1)){

if(R2_dist >= R2_frag_size[0]){

R2_frag_size[4] = R2_frag_size[3];

R2_frag_size[3] = R2_frag_size[2];

R2_frag_size[2] = R2_frag_size[1];

R2_frag_size[1] = R2_frag_size[0];

R2_frag_size[0] = R2_dist;

}

else if(R2_dist >= R2_frag_size[1]){

R2_frag_size[4] = R2_frag_size[3];

R2_frag_size[3] = R2_frag_size[2];

R2_frag_size[2] = R2_frag_size[1];

R2_frag_size[1] = R2_dist;

}

else if(R2_dist >= R2_frag_size[2]){

R2_frag_size[4] = R2_frag_size[3];

R2_frag_size[3] = R2_frag_size[2];

R2_frag_size[2] = R2_dist;

}

else if(R2_dist >= R2_frag_size[3]){

R2_frag_size[4] = R2_frag_size[3];

R2_frag_size[3] = R2_dist;

}

R2_dist = 1;

}

else{ R2_dist++; }

if(R1insertedUnit(s, cc, 1)){

if(R1_dist >= R1_frag_size[0]){

R1_frag_size[4] = R1_frag_size[3];

R1_frag_size[3] = R1_frag_size[2];

R1_frag_size[2] = R1_frag_size[1];

R1_frag_size[1] = R1_frag_size[0];

R1_frag_size[0] = R1_dist;

}

else if(R1_dist >= R1_frag_size[1]){

R1_frag_size[4] = R1_frag_size[3];

R1_frag_size[3] = R1_frag_size[2];

R1_frag_size[2] = R1_frag_size[1];

R1_frag_size[1] = R1_dist;

}

else if(R1_dist >= R1_frag_size[2]){

R1_frag_size[4] = R1_frag_size[3];

R1_frag_size[3] = R1_frag_size[2];

R1_frag_size[2] = R1_dist;

}

else if(R1_dist >= R2_frag_size[3]){

R1_frag_size[4] = R1_frag_size[3];

R1_frag_size[3] = R1_dist;

}

R1_dist = 1;

}

else{ R1_dist++; }

}

if(R2_dist >= R2_frag_size[0]){

R2_frag_size[4] = R2_frag_size[3];

R2_frag_size[3] = R2_frag_size[2];

R2_frag_size[2] = R2_frag_size[1];

R2_frag_size[1] = R2_frag_size[0];

R2_frag_size[0] = R2_dist;

}

else if(R2_dist >= R2_frag_size[1]){

R2_frag_size[4] = R2_frag_size[3];

R2_frag_size[3] = R2_frag_size[2];

R2_frag_size[2] = R2_frag_size[1];

R2_frag_size[1] = R2_dist;

}

else if(R2_dist >= R2_frag_size[2]){

R2_frag_size[4] = R2_frag_size[3];

R2_frag_size[3] = R2_frag_size[2];

R2_frag_size[2] = R2_dist;

}

else if(R2_dist >= R2_frag_size[3]){

R2_frag_size[4] = R2_frag_size[3];

R2_frag_size[3] = R2_dist;

}

if(R1_dist >= R1_frag_size[0]){

R1_frag_size[4] = R1_frag_size[3];

R1_frag_size[3] = R1_frag_size[2];

R1_frag_size[2] = R1_frag_size[1];

R1_frag_size[1] = R1_frag_size[0];

R1_frag_size[0] = R1_dist;

}

else if(R1_dist >= R1_frag_size[1]){

R1_frag_size[4] = R1_frag_size[3];

R1_frag_size[3] = R1_frag_size[2];

R1_frag_size[2] = R1_frag_size[1];

R1_frag_size[1] = R1_dist;

}

else if(R1_dist >= R1_frag_size[2]){

R1_frag_size[4] = R1_frag_size[3];

R1_frag_size[3] = R1_frag_size[2];

R1_frag_size[2] = R1_dist;

}

else if(R1_dist >= R2_frag_size[3]){

R1_frag_size[4] = R1_frag_size[3];

R1_frag_size[3] = R1_dist;

}

}

/* Name: Random Number Generator

* Input: n/a

*

* Output: a double in the range of 0≤X<0

* Function: genrate a random number

*

*/

double prob(void){

double random;

do{

random = rd_uniform(0,1);

}while(random >= 1);

return random;

}

/* Name: Clustered Number Generator

* Input: s - determines how clustered the numbers generated are (lower numbers = more clustered)

*

* Output: a double in the range of 0≤X<0 biased towards 0.5

* Function: genrate a random number following a normal distribution

*

*/

double Normal(double s){

double random;

do{

random = rd_normal(1.5,s);

}while((random/3.0) < 0 || (random/3.0) >= 1);

return random/3.0;

}

/* Name: Random Stagger Generator

* Input: max - the maximum size the stagger can be

*

* Output: an int in the range of 1≤X≤max

* Function: genrate a random number units to stagger the loci during recombination

*

*/

int rStagger(int max){

if(max<=0){ return 0;}

else{ return (1 + (int)(prob() * max)) * unitSize; }

}

/* Name: Truncation Number Convertor

* Input: id1 - first character used to identify the truncation

* id2 - second character used to identify the truncation

*

* Output: an int that can be used to identify the specific truncation

* Function: converts a truncation identifier from 2 chars to an int

*

*/

int trunc_num(char id1, char id2){

return (((int)(id1) - 65) * 26) + ((int)(id2) - 65);

}
